# Distinct Laboratory and Clinical Features of Metabolic and Alcohol-Related Liver Disease (MetALD): A Systematic Review and Meta-Analysis

**DOI:** 10.1007/s13679-026-00696-6

**Published:** 2026-03-06

**Authors:** Maria Tampaki, Vasileios Lekakis, Christos Chologkitas, Stergios Α. Polyzos, Evangelos Cholongitas

**Affiliations:** 1 First Academic Department of Gastroenterology, Medical School of National, Laiko General Hospital, Kapodistrian University of Athens, Athens, 11527 Greece; 2 First Department of Internal Medicine, Laiko General Hospital, Medical School of National, Kapodistrian University of Athens, 17 Agiou Thoma St., Athens, 11527 Greece; 3 First Laboratory of Pharmacology, School of Medicine, Aristotle University of Thessaloniki, Thessaloniki, Greece

**Keywords:** MetALD, MASLD, ALD, Steatotic liver disease, Liver enzymes, Metabolic dysfunction

## Abstract

**Purpose of review:**

We aimed to perform a systematic review and meta-analysis of observational studies, primarily to compare laboratory findings, including liver function tests, and metabolic parameters, between patients with Metabolic and alcohol-related/associated liver disease (MetALD) and those with metabolic dysfunction-associated steatotic liver disease (MASLD) or alcohol-related liver disease (ALD).

**Recent findings:**

MetALD is a newly recognized subtype of steatotic liver disease (SLD) characterized by the synergistic impact of metabolic dysfunction and alcohol consumption. Data on its clinical and laboratory profile remain limited.

**Summary:**

Thirty-three studies including 7,504,674 individuals were analyzed. Compared to MASLD, MetALD was associated with higher aspartate aminotransferase (AST), alanine aminotransferase (ALT), gamma glutamyl transferase (GGT), higher triglycerides (TG) and high density lipoprotein-cholesterol (HDL-C), lower low density lipoprotein-cholesterol (LDL-C), and higher systolic and diastolic blood pressure, but lower body mass index (BMI) and hemoglobin A1c (HbA1c). Compared to ALD, MetALD showed lower liver enzymes, while most metabolic parameters were similar.

**Conclusions:**

MetALD has distinct laboratory and metabolic features compared to MASLD, while differences with ALD are less pronounced. These findings favor MetALD as a distinct entity warranting further, more focused research.

**Supplementary Information:**

The online version contains supplementary material available at 10.1007/s13679-026-00696-6.

## Introduction

Following the Delphi Consensus of 2023, the entity of metabolic and alcohol-related/associated liver disease (MetALD) was introduced to differentiate patients with metabolic dysfunction-associated steatotic liver disease (MASLD) who consume increased amount of alcohol (140–350 g/week and 210–420 g/ week for women and men, respectively) [1]. Thus, MetALD lies between MASLD and alcohol-related liver disease (ALD), referring to the consumption of excessive amount of alcohol (≥ 350 g/week and ≥ 420 g/ week for women and men, respectively). According to the latest Guidelines and definitions, there is a continuum within the MetALD spectrum depending on the level of contribution of metabolic disorder and alcohol consumption [2].

Currently, there is evidence supporting that MASLD prevalence is similar to that of non-alcoholic fatty liver disease (NAFLD) [3], while data regarding the prevalence of MetALD are based on smaller studies that need further confirmation [2]. As a new entity, MetALD is not yet associated with specific patient characteristics in terms of metabolic profiles and alcohol habits, making its diagnosis and epidemiology challenging. It is possible that patients across steatotic liver disease (SLD) subtypes share common characteristics that are prominent depending on disease category [4]. On the other hand, patients with MetALD may have some distinct features based on sex, ethnic, and socioeconomic factors [4]. An emerging issue is whether this “newly discovered” clinical entity is also associated with different laboratory characteristics and severity of liver fibrosis compared to MASLD and ALD.

The key element that differentiates MetALD is the combined adverse effect of metabolic dysfunction and alcohol consumption [5]. This synergy undoubtedly affects liver disease and is associated with systemic clinical implications [5, 6]. MASLD and the preceding term of NAFLD have been associated with mildly elevated transaminases [alanine aminotransferase (ALT) > aspartate transaminase (AST)] and/or gamma-glutamyl transferase (GGT)] [7, 8]. However, a significant proportion of patients may have normal-range ALT levels, especially in cases of advanced fibrosis [9]. ALD has also some typical laboratory findings [10]. In terms of lipid and glycemic profile, MASLD diagnosis is strictly connected to abnormal values, while heavy alcohol consumption has been associated with elevated total cholesterol and triglyceride (TG) levels and increased risk for type 2 diabetes mellitus (T2DM) [11, 12]. Interestingly, there is still no evidence that MetALD has a distinct biochemical or metabolic pattern compared to other SLD subtypes that may facilitate patient differentiation. In our recent meta-analysis [4], there were no significant differences in terms of prevalence of metabolic components between patients with MetALD and other SLD subtypes. If these disease parameters are elucidated, the determination of MetALD prognosis regarding cardiovascular disease (CVD) risk, liver-related outcomes and overall survival may be facilitated.

Based on the above and in order to enhance the existing knowledge on this new entity of MetALD, we performed a systematic review and meta-analysis of observational studies, primarily aiming to compare laboratory findings, including liver function tests, and metabolic parameters, between patients with MetALD and those with MASLD or ALD.

## Methods

### Data Sources and Searches

Medline/PubMed, Embase, Scopus, and Cochrane databases were systematically searched for studies published up to May 2025, following the Meta-analysis of Observational Studies in Epidemiology (MOOSE) Checklist (PROSPERO ID: CRD420251160017). The database-specific search strings are described in detail in the Appendix. In addition, alerts were set up in all the databases to capture newly published relevant studies. An independent manual search was also conducted, covering the European Association for the Study of Liver (EASL) Liver Congress 2025, EASL Liver Congress 2024, American Association for the Study of Liver Diseases (AASLD) Liver Meeting 2024, Asian Pacific Association for the Study of the Liver (APASL) Conference 2024, and the reference lists of the included articles. When essential information could not be retrieved, the corresponding authors were contacted.

### Study Selection

Eligibility criteria were determined using the PICO framework: P: adult patients with MetALD; C: adult patients with MASLD or ALD; O: laboratory findings, including liver function tests, and metabolic parameters.

Studies published in English were considered eligible if they met the following criteria: (1) they were observational studies, including cohort, case-control, or cross-sectional; (2) they included adult patients (> 18 years); (3) they used the new nomenclature for SLD and its sub-types (MASLD, MetALD, ALD) according to the Delphi consensus of 2023; (4) they described the diagnostic method for SLD and its sub-types; and (5) they reported laboratory findings. All the included studies used the same diagnostic criteria for MASLD, MetALD and ALD based on the above mentioned Delphi consensus [1] and the joined EASL, European Association for the Study of Diabetes (EASD) and European Association for the Study of Obesity (EASO) Clinical Practice Guidelines on the management of MASLD [2]. Studies were excluded if they were case reports, review articles, interventional studies, animal or cell line research, editorials, guidelines, opinions, commentaries, hypotheses, or book chapters. In cases of overlapping study populations, only the largest study was included. Studies referring to special populations or studies based on mixed databases were also excluded. Two reviewers (MT, VL) independently screened the titles and abstracts to identify potentially eligible studies, with a third blinded reviewer (EC) resolving any disagreements. Full-text articles were then assessed by EC to ensure they met the eligibility criteria. Conference abstracts were eligible for inclusion, if they met al.l predefined inclusion and exclusion criteria and reported sufficient quantitative data relevant to the outcomes of interest. When both a conference abstract and a subsequent full-text publication referred to the same study, only the full manuscript was included to avoid duplicate data.

### Data Extraction and Quality Assessment

Two authors (MT, VL) independently extracted the following information from each study: first author, publication date, country of origin, study design, sample size and type of cohort, method used to evaluate SLD and its sub-types (MASLD, MetALD, ALD), tool to evaluate alcohol consumption and sex distribution. Clinical [age, systolic blood pressure (SBP), diastolic blood pressure (DBP), body mass index (BMI), waist circumference (WC)] and laboratory findings [platelet count (PLT), liver function tests (LFTs), estimated glomerular filtration rate (eGFR), lipid and glycemic profile] as well as non-invasive tests (NITs) for evaluation of severity of liver disease were also recorded for each SLD sub-type, when available. Study quality was assessed using the Newcastle-Ottawa Scale (NOS) [13]. Each study was independently evaluated on eight items grouped into three categories: selection of study groups, comparability of groups, and ascertainment of exposure or outcome for case-control or cohort studies, respectively. The NOS score ranges from 0 (lowest; poor quality) to 9 (highest; high quality). Studies scoring seven or more stars were considered of high quality, those with five to six stars were considered of medium quality, and those with fewer than five stars were considered of low quality. Only studies applying the new SLD nomenclature according to the above-mentioned Delphi consensus and the joined EASL–EASD–EASO guidelines were included, to ensure diagnostic consistency across studies.

### Study Outcomes

The primary outcome of our study was the comparison of laboratory findings, including LFTs, and metabolic parameters, between patients with MetALD and those with MASLD or ALD. The secondary outcome was the comparison of NITs, including Fatty Liver Index (FLI), Fibrosis-4 (FIB-4) score, NAFLD fibrosis score (NFS), liver stiffness measurements (LSM) and magnetic resonance elastography (MRE) between those patient groups.

### Data Synthesis and Statistical Analysis

For dichotomous outcomes a generalized linear mixed model (GLMM) [14] was used to estimate pooled proportions. For single-study proportions, 95% confidence intervals (CIs) were calculated using the Clopper–Pearson method [15]. For continuous outcomes, meta-analyses were conducted using study-level effect sizes. For each study, we extracted group-specific summary statistics [mean, standard deviation (SD) and sample size] for MetALD and the comparator group (MASLD or ALD). When outcomes were reported in different measurements units across studies, their values were converted to common units prior to synthesis using standard conversion factors. All laboratory and clinical outcomes were analyzed in their standard units (e.g., liver enzymes in IU/L, blood pressure in mmHg, BMI in kg/m², eGFR in mL/min/1.73 m², liver stiffness in kPa). The primary effect measure for continuous outcomes was the mean difference (MD). Study-specific MDs and corresponding SDs were pooled using the inverse-variance method under a random-effects model (DerSimonian–Laird approach), due to the expected moderate-to-high heterogeneity [16]. If mean and/or SD for specific outcome(s) could not be retrieved or calculated based on standard equations, the study was not included in the quantitative synthesis for the specific outcome(s). When studies reported medians and interquartile range (IQRs) or 25th-75th percentiles instead of means and SDs, we converted these to approximate means and SDs using established methods.

Between-study variance (τ²) was estimated using the maximum likelihood method. Heterogeneity was assessed using Cochran’s Q test, the I² statistic and τ² [17]. The computations for prediction intervals (PI) were also performed [18]. To explore potential sources of heterogeneity, we conducted subgroup analyses according to geographic region/ethnicity (Asian vs. non-Asian populations) and diagnostic modality for SLD (US-based diagnosis vs. serum-based indices/biomarkers). The subgroup analysis was conducted only when there were at least three available studies. Subgroup-specific pooled estimates and *p*-values are presented in Supplementary Tables. Furthermore, sensitivity analyses were conducted by excluding conference abstracts and studies with low methodological quality (NOS score ≤ 5). Due to the lack of harmonized multivariable-adjusted effect estimates, pooled analyses were based on unadjusted group-level summary statistics. Assessment of small-study effects and potential publication bias was performed for outcomes with at least 10 included studies, using visual inspection of funnel plots and Egger’s regression test. For outcomes with fewer than 10 studies, formal assessment was not performed, because statistical tests for small-study effects are underpowered in this setting. A two-sided P value < 0.05 was considered statistically significant. Analyses were conducted using R version 4.3.1 with the ‘meta’ and ‘metafor’ packages [19].

## Results

**Fig. 1:**
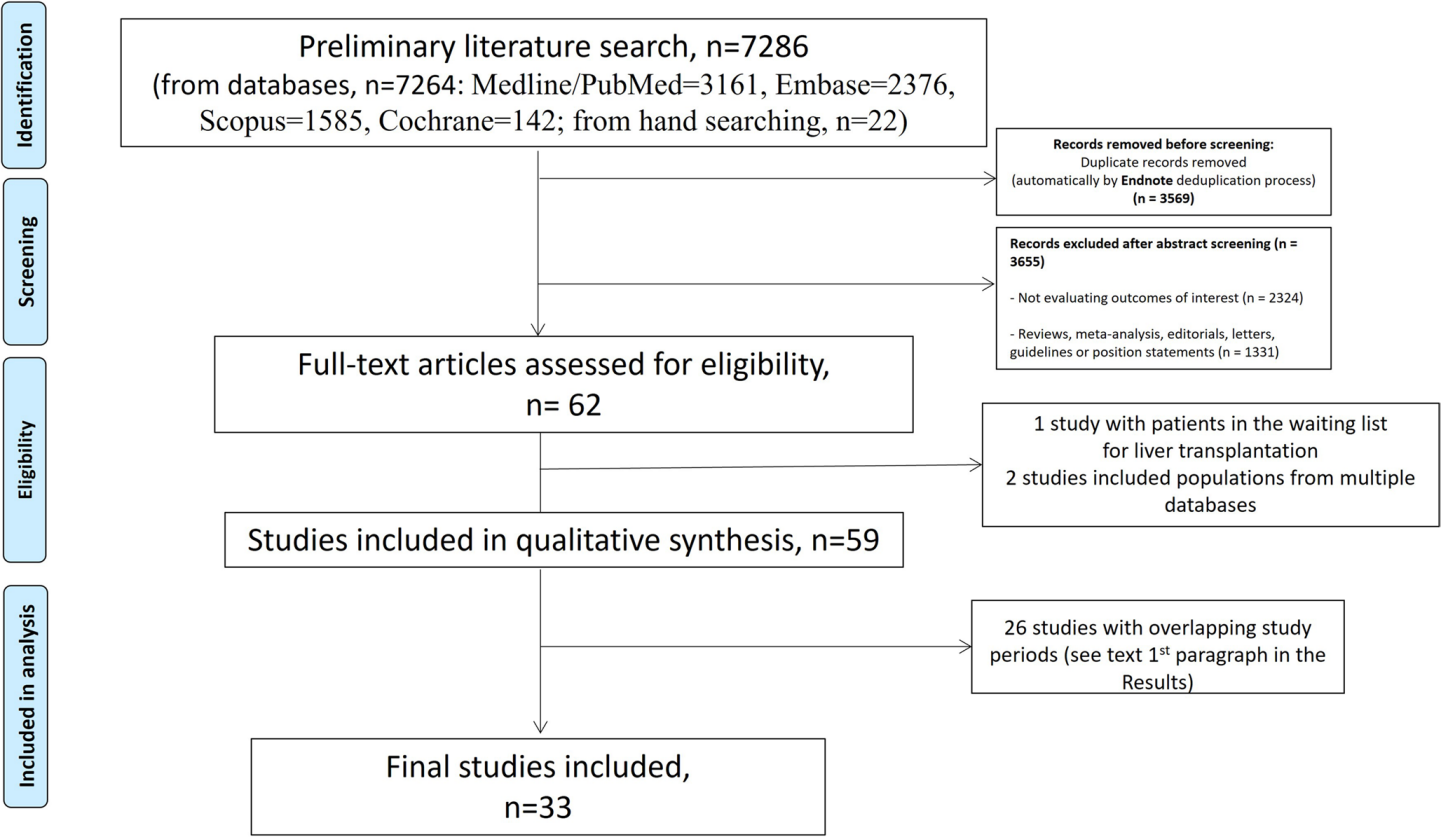
PRISMA flow diagram of study selection

In total, 7286 articles were initially identified from the literature search; 62 of them fulfilled the eligibility criteria and underwent further evaluation [20–81] (Fig. 1). Seventeen studies from the USA population [34, 50, 53–66, 77] had overlapping study periods and populations (NHANES 1988–1994, 2017–2020, 1999–2006 and 1999–2018), and therefore only the two largest studies with distinct study periods [34, 50] were included. Similarly, four studies [21, 67, 68, 70] from Korea and seven studies from the UK [27, 71–76] had overlapping populations (NHIS and UK Biobank, respectively), so the two largest studies were included [21, 27]. Finally, two studies [79, 80] were excluded since they included populations from multiple databases, while one study [81] was excluded since the epidemiological data were derived from the UNOS database and patients in the waiting list for liver transplantation. Thus, 33 studies [20–52] that evaluated the laboratory findings of patients with MetALD fulfilled the eligibility criteria and were included in the systematic review and meta-analysis. Thirteen studies were derived from Korea [21, 22, 24, 25, 28, 30, 37–41, 43, 46], four from Japan [26, 31, 32, 42], three from the USA [34, 45, 50], two from China [20, 33], Taiwan [29, 45], and Spain [36, 52], one from the UK [27], one from Denmark [23], Sweden [47], Finland [35], Chile [48], and Canada [49] and one international study [51] (Tables S1a–c).

### Comparison Between MetALD and MASLD

#### Age

**Fig. 2:**
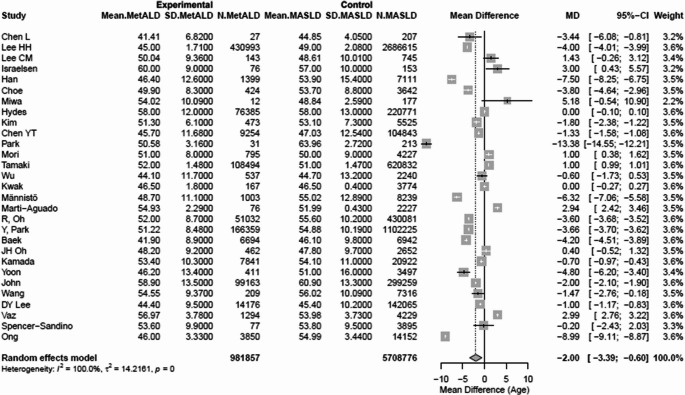
Forest plot of studies comparing the pooled mean age of MetALD patients and MASLD patients

Patients with MetALD were younger compared to those with MASLD [pooled mean: 49.7 vs 51.8 years, respectively; MD -2.00 (95%CI: -3.39; -0.60), *p *= 0.005; heterogeneity, τ^2^ = 14.21 *p *< 0.0001] (Fig. 2) (Table S2).

#### Liver Function Tests

**Fig. 3:**
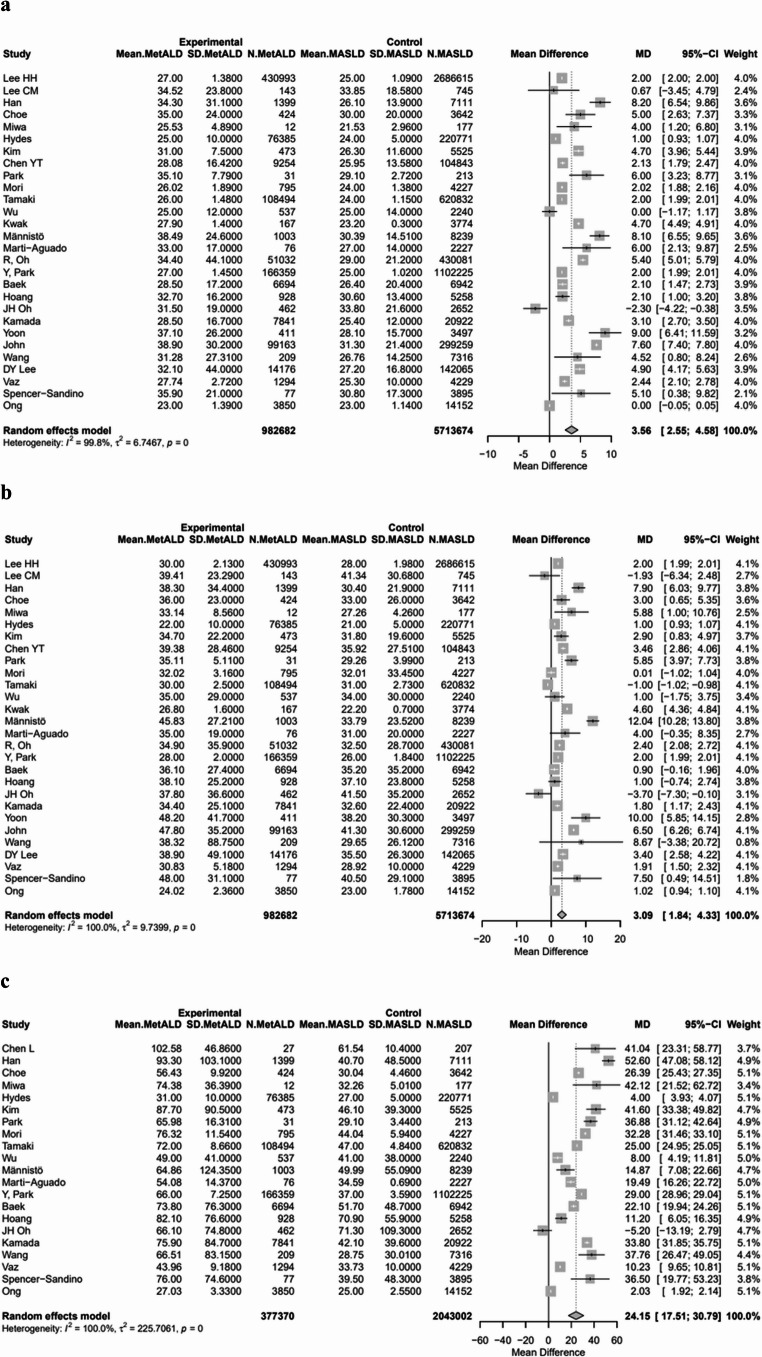
Forest plot of studies comparing the pooled mean levels of **a** aspartate transaminase (AST), **b** alanine transaminase (ALT), **c** gamma-glutamyl transferase (GGT) between MetALD and MASLD patients

In terms of LFTs, patients with MetALD had significantly higher pooled mean levels of AST [28.4 vs. 25.6 IU/L, MD 3.56 (95%CI: 2.55; 4.58), *p* < 0.0001; τ^2^ = 6.74, heterogeneity, *p* < 0.0001], ALT [31.4 vs. 29.1 IU/L, MD 3.09 (95%CI: 1.84; 4.33), *p* < 0.0001; τ^2^ = 9.73, heterogeneity, *p* < 0.0001] and GGT [60.7 vs. 39.2 ΙU/L, MD: 24.15 (95%CI: 17.51; 30.79), *p* < 0.0001; τ^2^ = 225.70, heterogeneity, *p* < 0.0001], compared to patients with MASLD (Figs. 3a–c) (Table S2).

#### Metabolic Parameters

**Fig. 4:**
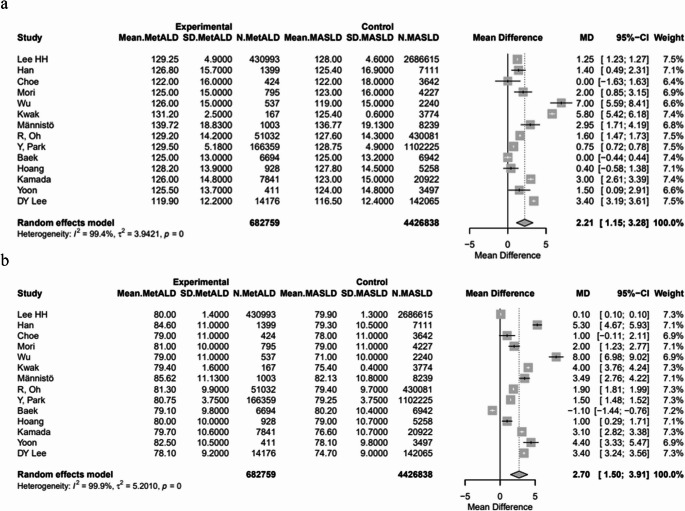
Forest plot of studies comparing the pooled mean **a** systolic (SBP) and **b** diastolic (DBP) blood pressure between MetALD and MASLD patients

**Fig. 5:**
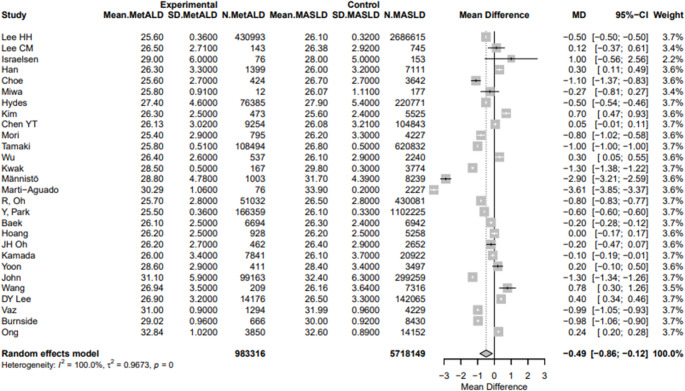
Forest plot of studies comparing the pooled mean of body mass index (BMI) between MetALD and MASLD patients

**Fig. 6:**
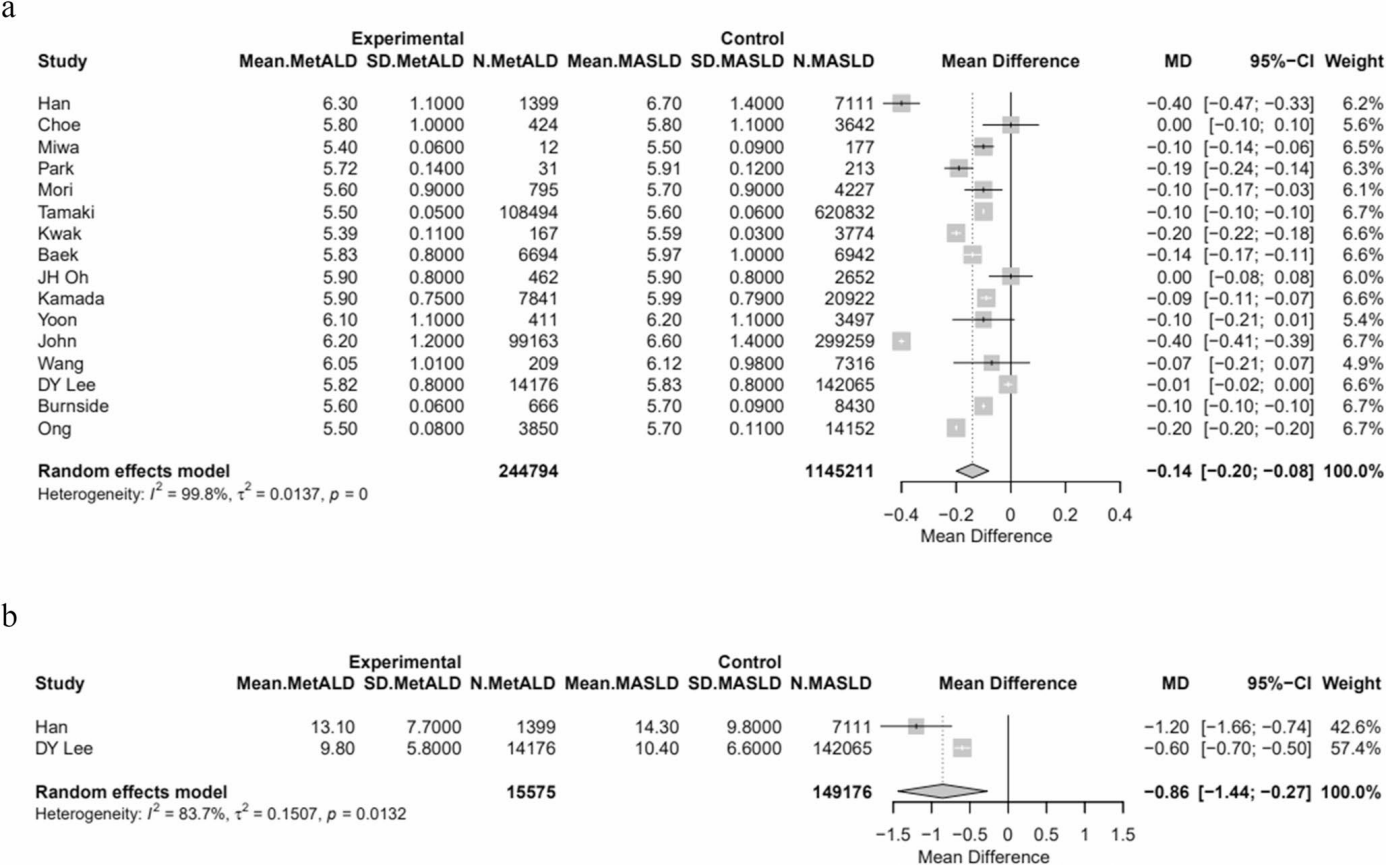
Forest plot of studies comparing the pooled mean **a** hemoglobin A1C (HbA1C) levels, **b** insulin levels between MetALD and MASLD patients

**Fig. 7:**
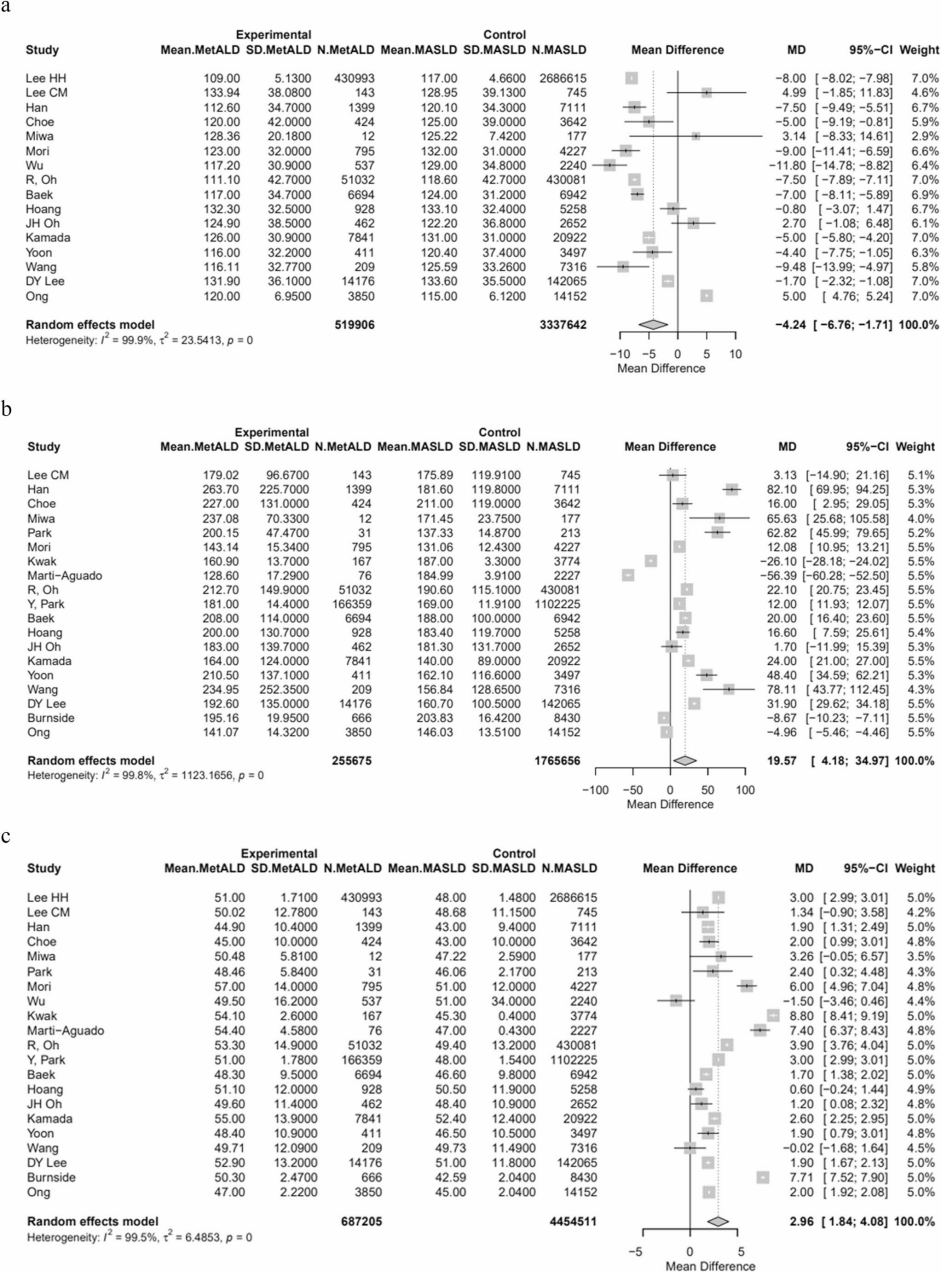
Forest plot of studies comparing the pooled mean levels of **a** low density lipoprotein cholesterol (LDL-C), **b** triglycerides (TG), and **c** high density lipoprotein cholesterol (HDL-C) between MetALD and MASLD patients

The pooled mean SBP and DBP were statistically higher in patients with MetALD [129.03 vs. 127.74 mmHg, MD 2.21 (95%CI: 1.15; 3.28) for SBP, *p* < 0.0001; τ^2^ = 3.94, heterogeneity, *p* < 0.0001; and 80.25 vs. 79.5 mmHg, MD 2.7 (95%CI: 1.50; 3.91) for DBP, *p* < 0.0001; τ^2^ = 5.20, heterogeneity, *p* < 0.0001, respectively] (Figs. 4a, b). On the other hand, MetALD patients, compared to patients with MASLD, had statistically lower pooled mean of BMI [26.3 vs. 26.7 kg/m^2^, MD -0.49 (95%CI: -0.86; -0.12), *p* = 0.0093; τ^2^ = 0.96, heterogeneity, *p* < 0.0001] (Fig. 5), glycated hemoglobin A1_C_ (HbA1_C_) [5.8 vs. 5.9%, MD -0.14 (95%CI: -0.20; -0.08), *p* < 0.0001; τ^2^ = 0.01, heterogeneity, *p* < 0.0001] (Fig. 6a) and insulin levels [10.1 vs. 10.6 mIU/L, MD -0.86 (95%CI: -1.44; -0.27), *p* = 0.0039; τ^2^ = 0.15, heterogeneity, *p* = 0.0132] (Fig. 6b). Regarding lipid profile, MetALD patients, as compared to MASLD patients, had lower pooled mean levels of low density lipoprotein-cholesterol (LDL-C) [110.4 vs. 118.1 mg/dL, MD -4.24 (95%CI: -6.76; -1.71), *p* = 0.001; τ^2^ = 23.54, heterogeneity, *p* < 0.0001] (Fig. 7a**)**, but higher pooled mean TG levels [188.1 vs. 173.4 mg/dL, MD 19.57 (95%CI: 4.18; 34.97), *p* = 0.0127, τ^2^ = 1123.16, heterogeneity, *p *< 0.0001] (Fig. 7b**)** and pooled mean high density lipoprotein-cholesterol (HDL-C) [pooled mean: 51.2 vs. 48.2 mg/dL, MD 2.96 (95%CI: 1.84; 4.08), *p* < 0.0001; τ^2^ = 6.48, heterogeneity, *p* < 0.0001] (Fig. 7c) (Table S2).

On the other hand, the pooled mean levels of total cholesterol [202.2 vs. 203.9 mg/dL, MD 1.98 (95%CI: -0.85; 4.80), *p* = 0.171; heterogeneity, *p* < 0.0001] (Fig. S1), fasting glucose [98.8 vs. 99.9 mg/dL, MD 0.35 (95%CI: -2.91; 3.60), *p* = 0.8347; heterogeneity, *p* < 0.0001] (Fig. S2), Homeostatic Model Assessment for Insulin Resistance (HOMA-IR) [2.3 vs. 2.69, MD -0.05 (95%CI: -0.24; 0.15), *p* = 0.6427; heterogeneity, *p* < 0.0001] (Fig. S3), and WC (87.4 vs. 88.3 cm, MD -0.79 (95%CI: -2.58; 1.00), *p* = 0.3879; heterogeneity, *p *< 0.0001] (Fig. S4) did not differ significantly between the two groups (Table S2).

#### Other Laboratory Findings

**Fig. 8:**
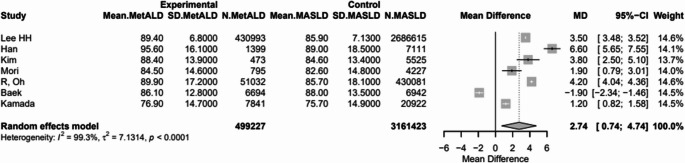
Forest plot of studies comparing the pooled mean lower platelet count (PLT) between MetALD and MASLD patients

**Fig. 9:**
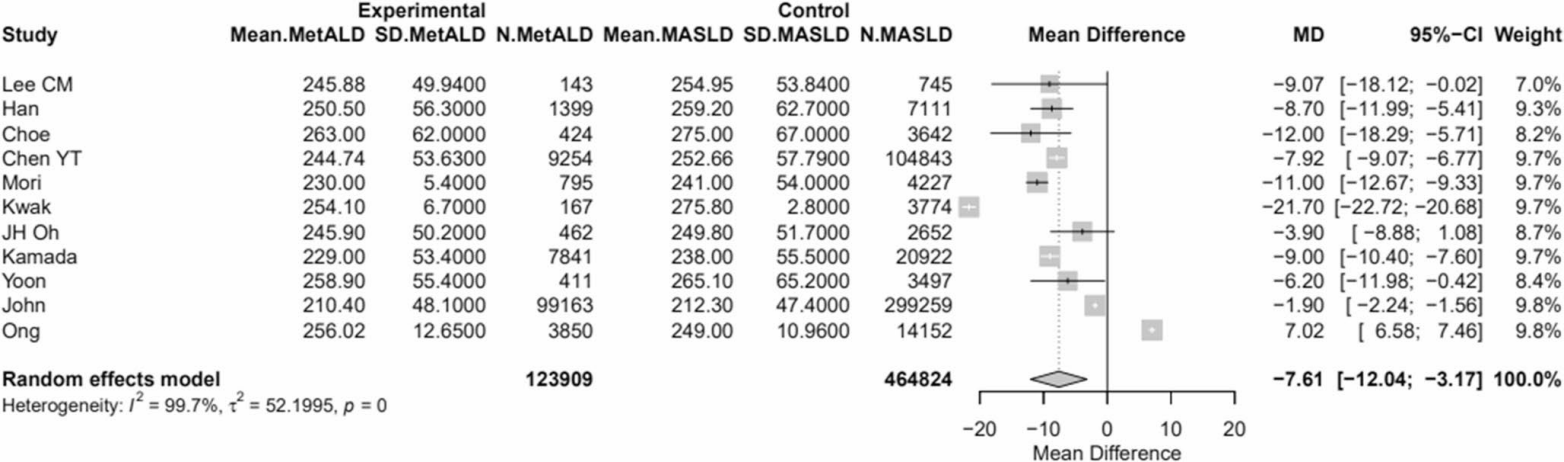
Forest plot of studies comparing the pooled mean levels of estimated glomerular filtration rate (eGFR) between MetALD and MASLD patients

MetALD, compared to MASLD, was associated with higher eGFR [89.2 vs. 85.8 mL/min/1.73 m², MD 2.74 (95%CI: 0.74; 4.74), *p* = 0.0072; τ^2^ = 7.13, heterogeneity, *p* < 0.0001] (Fig. 8), and lower PLT (216.7 vs. 226.3 10^9/L, MD -7.61 (95%CI: -12.4; -3.17), *p* = 0.0008; τ^2^ = 52.19 heterogeneity, *p* < 0.0001] (Fig. 9) (Table S2).

#### Non-Invasive Tests

**Fig. 10:**
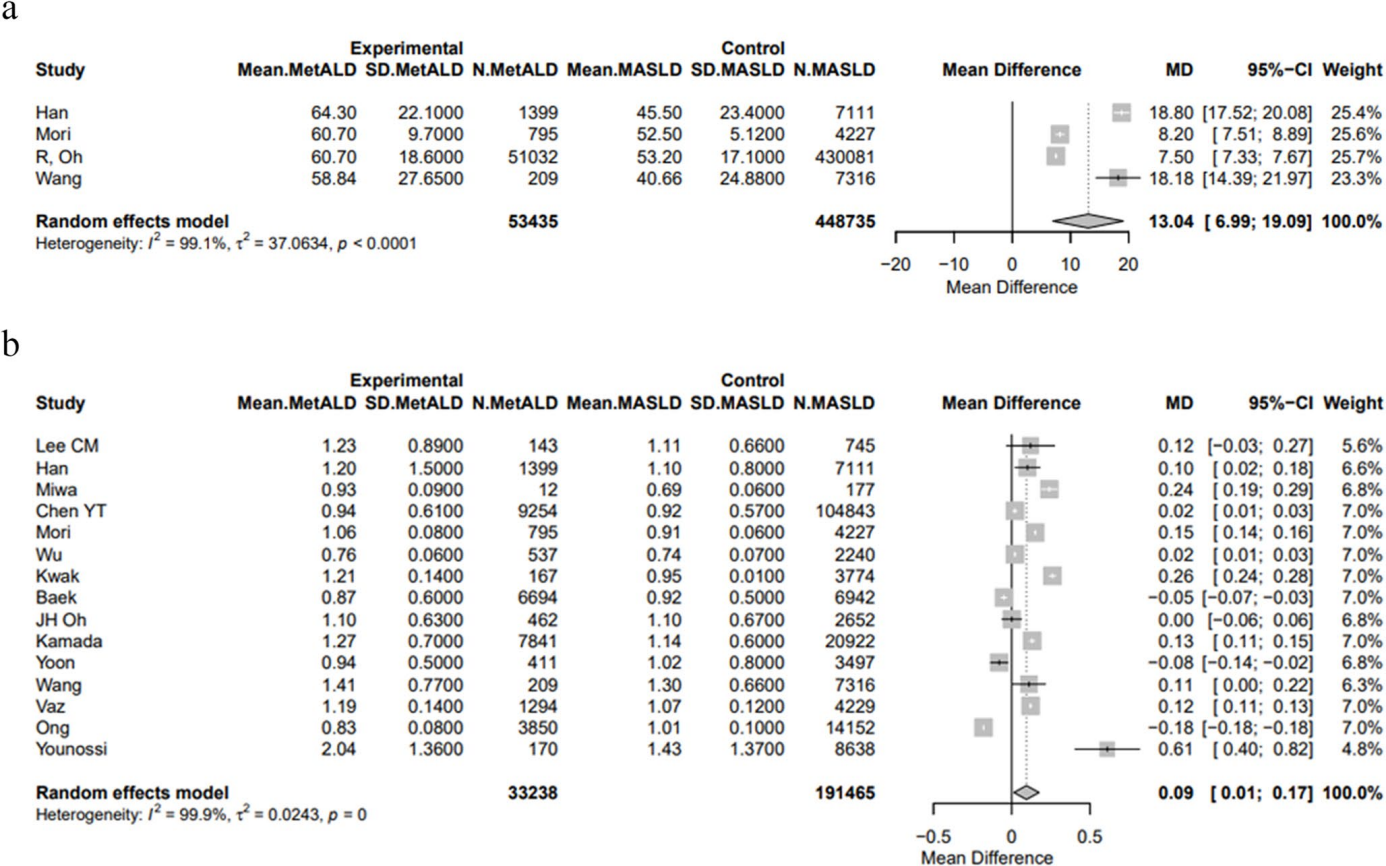
Forest plot of studies comparing the pooled mean levels of **a** fatty liver index (FLI) and **b** fibrosis-4 (FIB-4) score between MetALD and MASLD patients

FLI was higher in MetALD patients compared to those with MASLD [pooled mean: 60.79 vs. 52.87, MD 13.04 (95%CI: 6.99; 19.09), *p* < 0.0001; τ^2^ = 37.06, heterogeneity, *p* < 0.0001](Fig. 10a). The pooled mean of FIB-4 score was statistically higher in patients with MetALD compared to patients with MASLD [1.03 vs. 1.00, MD 0.09 (95%CI: 0.01; 0.17), *p* = 0.0235; τ^2^ = 0.02, heterogeneity, *p* < 0.0001**]** (Fig. 10b). On the contrary, the pooled mean of NFS was similar between MetALD and MASLD patients [-2.01 vs. -2.27, MD 0.03 (95%CI: -0.14; 0.20), *p* = 0.7022; heterogeneity, *p* < 0.0001], as well as LSM [7.19 vs. 9.22 kPa, MD 0.01 (95%CI: -1.24; 1.27), *p* = 0.9816; heterogeneity, *p* < 0.0001], and MRE measurements [2.66 vs. 2.68 kPa, MD -0.17 (95%CI: -0.71; 0.37), *p* = 0.5393; heterogeneity, *p* = 0.0002] (Figs. S5a–c; Table S3).

### Comparison Between MetALD and ALD

#### Age

Age did not differ significantly between patients with MetALD and those with ALD [pooled mean: 54.7 vs. 53.6 years, MD 0.61 (95%CI: -0.65; 1.88), *p* = 0.3429; heterogeneity, *p* < 0.0001] (Fig. S6; Table S4).

#### Liver Function Tests

**Fig. 11:**
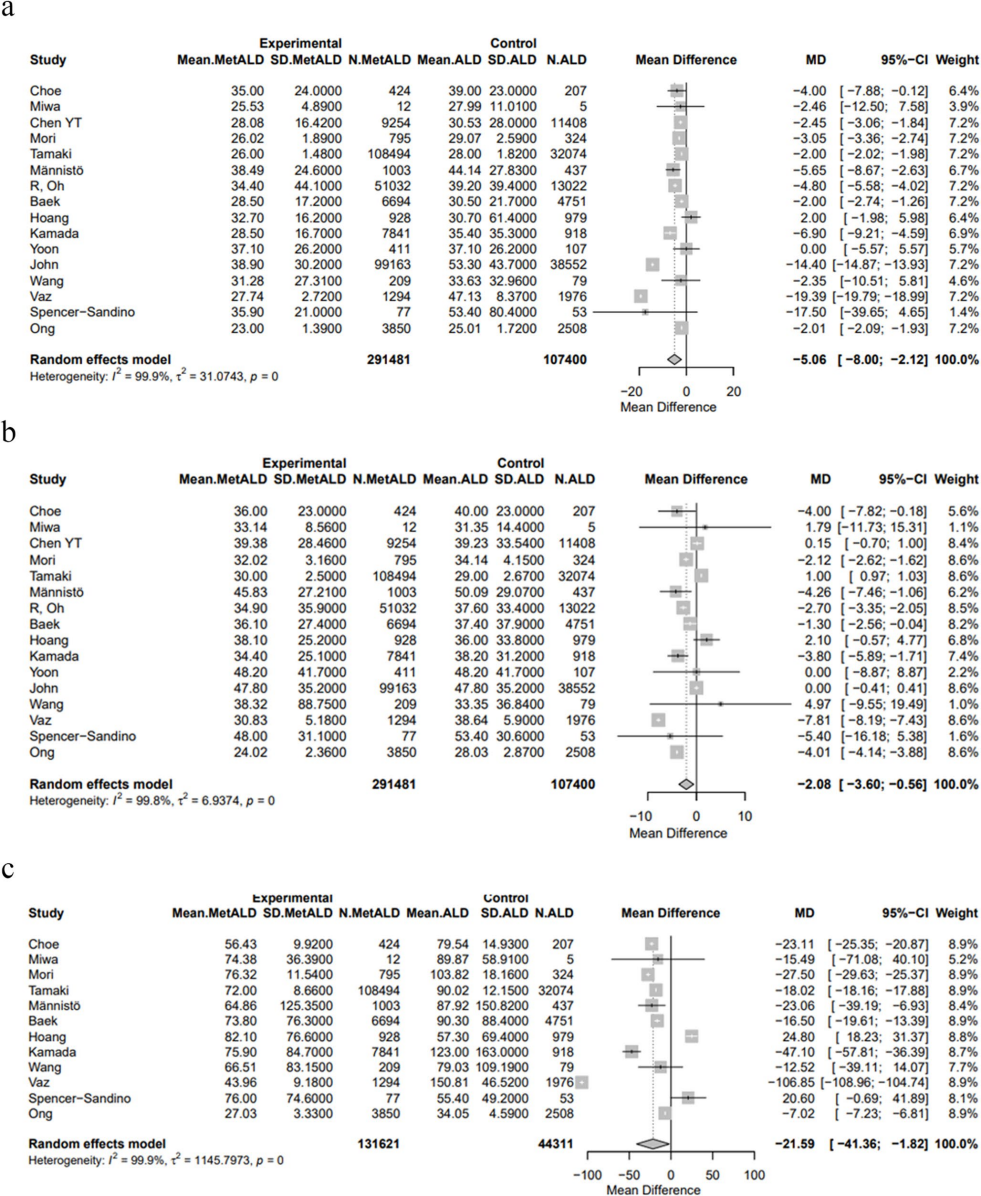
Forest plot of studies comparing the pooled mean levels of **a** aspartate transaminase (AST), **b** alanine transaminase (ALT), **c** gamma-glutamyl transferase (GGT) between MetALD and ALD patients

Patients with MetALD had significantly lower pooled mean levels of AST [32.1 vs. 39.3 IU/L, MD -5.06 (95%CI:-8; -2.12), *p* = 0.0007; τ^2^ = 31.07, heterogeneity, *p* < 0.0001], ALT [37.5 vs. 38.7 IU/L, MD -2.08 (95%CI: -3.60; -0.56), *p* = 0.0072; τ^2^ = 6.93, heterogeneity, *p* < 0.0001] and GGT [70.7 vs. 89.5 IU/L, MD: -21.59 (95%CI: -41.36; -1.82), *p* = 0.0323; τ^2^ = 1145.79 heterogeneity, *p* < 0.0001], compared to patients with ALD (Figs. 11a–c) (Table S4).

#### Metabolic Parameters

**Fig. 12:**
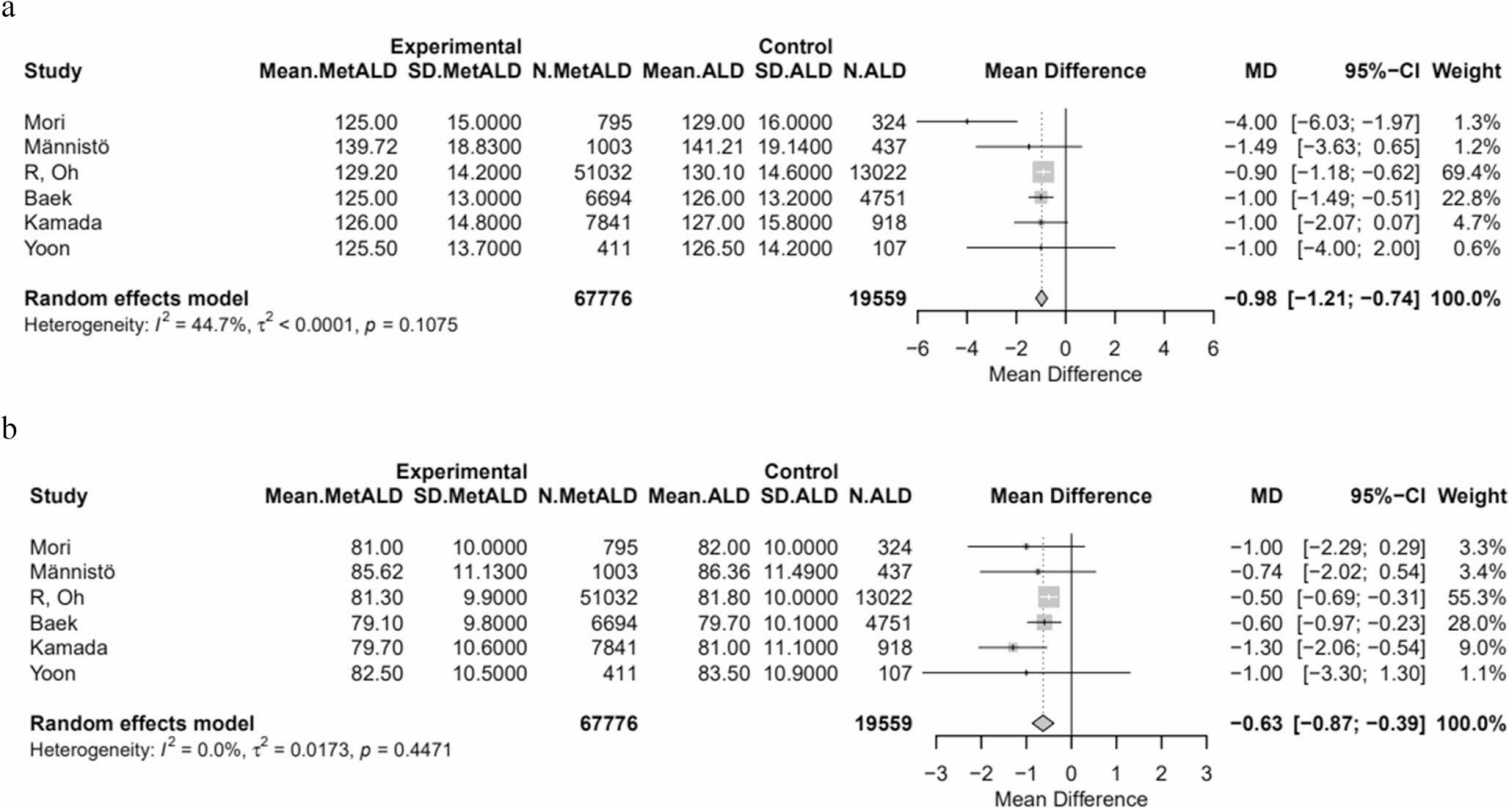
Forest plot of studies comparing the pooled mean **a** systolic (SBP) and **b** diastolic (DBP) blood pressure between MetALD and ALD patients

**Fig. 13:**
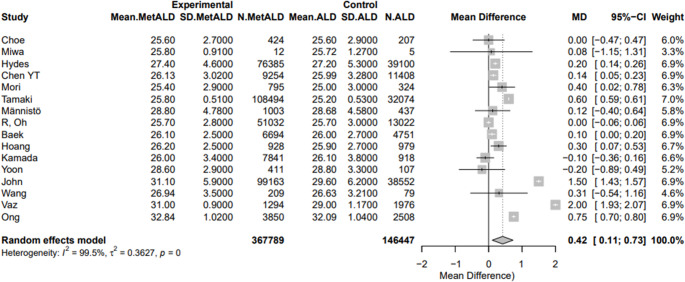
Forest plot of studies comparing the pooled mean of body mass index (BMI) between MetALD and ALD patients

**Fig. 14:**
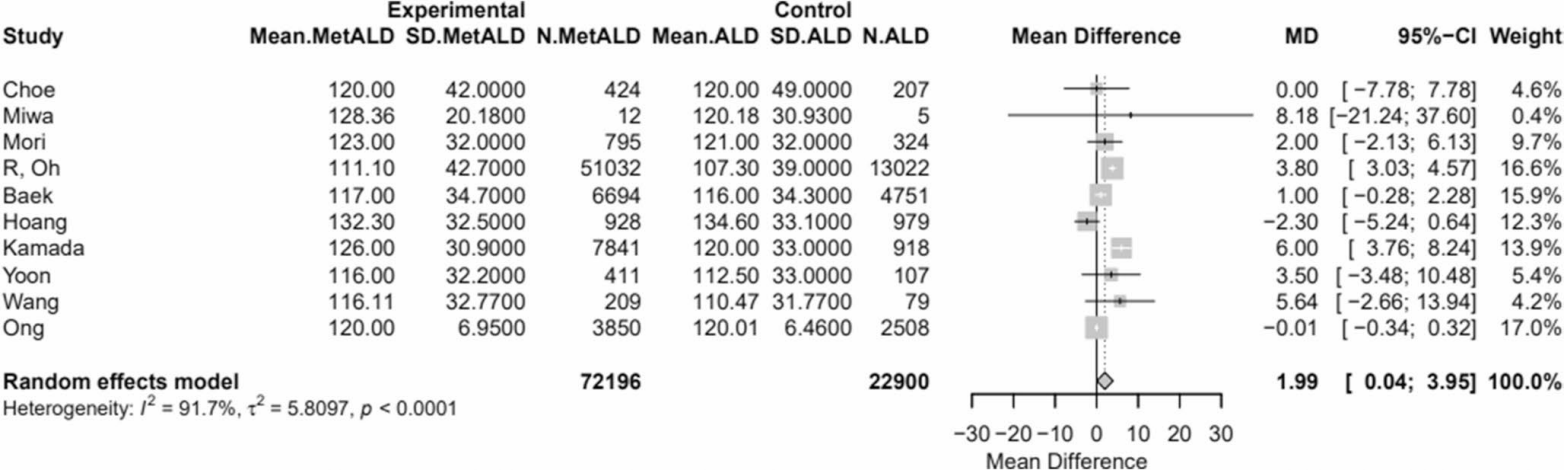
Forest plot of studies comparing the pooled mean levels of low-density lipoprotein cholesterol (LDL-C) between MetALD and ALD patients

SBP and DBP were statistically lower in patients with MetALD [pooled mean: 128.5 vs. 129.17 mmHg, MD -0.98 (95%CI: -1.21; -0.74), *p* < 0.0001; τ^2^ < 0.0001, heterogeneity, *p* = 0.1075; and 80.97 vs. 81.37 mmHg, MD -0.63 (95%CI: -0.87; -0.39), *p* < 0.0001; τ^2^ = 0.01 heterogeneity, *p* = 0.4471, respectively] (Figs. 12a, b). On the other hand, MetALD patients, compared to patients with ALD, had higher pooled mean of BMI [27.7 vs. 27.2 kg/m^2^, MD 0.42 (95%CI: 0.11; 0.73), *p* = 0.0084; τ^2^ = 0.36 heterogeneity, *p* < 0.0001] (Fig. 13) and LDL-C levels [114.2 vs. 112.5 mg/dL, MD 1.99 (95%CI: 0.04; 3.95; *p* = 0.0452; τ^2^ = 5.81, heterogeneity, *p* < 0.0001] (Fig. 14).

No significant differences were found regarding the pooled mean levels of HbA1_C_ [5.83 vs. 5.76%, MD 0.05 (95%CI: -0.01; 0.12), *p* = 0.1140; heterogeneity, *p* < 0.0001], fasting glucose levels [89.9 vs. 109.2 mg/dL, MD -6.22 (95%CI: -14.36; 1.91), *p* = 0.1338; heterogeneity, *p* < 0.0001] (Figs. S7a, b), total cholesterol [203.9 vs. 203.1 mg/dL, MD -0.03 (95%CI: -2.00; 1.94), *p* = 0.9790; heterogeneity, *p* < 0.0001], HDL-C [52.6 vs. 51.9 mg/dL, MD -1.08 (95%CI: -2.73; 0.57), *p* = 0.1983; heterogeneity, *p* < 0.0001], TGs [202.3 vs. 208.5 mg/dL, MD -8.68 (95%CI: -22.68; 5.33), *p* = 0.2245, heterogeneity, *p *< 0.0001] (Figs. S8a–c), HOMA-IR [1.37 vs. 1.34, MD 0.02 (95%CI: -0.03; 0.07), *p* = 0.4258; heterogeneity, *p* = 0.8446] (Figs. S9), and WC (89.8 vs. 91.3 cm, MD -0.22 (95%CI: -0.67; 023), *p* = 0.3350; heterogeneity, *p* < 0.0001] (Figs. S10) between the two groups (Table S4).

#### Other Laboratory Findings

MetALD and ALD did not differ regarding eGFR [pooled mean: 87.92 vs. 88.78 mL/min/1.73 m², MD -1.7 (95%CI: -3.58; 0.19), *p* = 0.0775; heterogeneity, *p* < 0.0001] (Figs. S11) and PLT [216.13 vs. 214.46 10^9/L, MD 1.69 (95%CI: -1.68; -5.06), *p* = 0.3255; heterogeneity, *p* < 0.0001 (Figs. S12; Table S4).

#### Non-Invasive Tests

FIB-4 score did not differ significantly between MetALD and ALD patients [pooled mean 1.01 vs. 1.14, MD -0.21 (95%CI: -0.49; 0.07), *p* = 0.1351; heterogeneity, *p* < 0.0001] (Figs. S13). An analysis of FLI, NFS and LSM/MRE values was not possible because there were no available studies comparing these parameters between MetALD and ALD population.

### Subgroup Analysis

In subgroup analyses comparing MetALD with MASLD, stratification by geographic region (Asian vs. non-Asian) and diagnostic modality (diagnosis of SLD based on serum or ultrasound) yielded findings consistent with the primary analysis (Figs. S14–S84; Tables S5–S12). Across Asian and non-Asian populations, MetALD generally exhibited modest, but statistically significant differences in liver enzymes and lipid parameters, while BMI and waist circumference showed minimal or inconsistent differences between groups. NITs remained largely comparable across subgroups.

Similarly, subgroup analyses comparing MetALD with ALD demonstrated a high degree of metabolic overlap across geographic regions (Asian and non-Asian), with most glycemic and lipid parameters remaining similar between groups (Figs. S85–S130; Tables S13–S16). Although minor differences in BMI and selected laboratory markers were observed in certain subgroups, these differences were generally modest and inconsistent.

#### Risk-of-Bias Assessment

Based on the Newcastle–Ottawa Scale, the majority of included studies were of moderate to high methodological quality. The distribution of NOS scores is presented in Table S1. Sensitivity analyses excluding low-quality studies (NOS ≤ 5) and conference abstracts yielded results consistent with the primary analyses (Figs. S131–S165; Tables S17–S20). The exclusion of individual studies did not meaningfully change the pooled estimates or their statistical significance.

#### Publication Bias Assessment

Funnel plots and Egger’s tests results are presented in the Supplementary Material (Figs. S166–S189). Based on the above analyses, no statistically significant evidence of small-study effects was observed.

### Age

Patients with MetALD were younger compared to those with MASLD [pooled mean: 49.7 vs. 51.8 years, respectively; MD -2.00 (95%CI: -3.39; -0.60), *p* = 0.005; heterogeneity, τ^2^ = 14.21 *p* < 0.0001] (Fig. 2) (Table S2).

## Discussion

This meta-analysis included 33 observational studies that evaluated patients with SLD. Regarding patient characteristics and laboratory findings, MetALD patients were younger, had higher LFTs levels (AST, ALT, GGT), higher eGFR, SBP, DBP, FIB-4 and FLI score, and lower PLT count compared to patients with MASLD. In terms of lipid profile, they had higher TGs and HDL-C, but lower LDL-C. Additionally, they had lower insulin, HbA1c and BMI than the MASLD population (Table 1). Compared to ALD, MetALD patients had lower AST, ALT, and GGT, SBP and DBP, higher LDL-C and BMI while other laboratory, metabolic and fibrosis parameters were similar (Table 2). These findings suggest that patients with MetALD may be characterized by different metabolic phenotypes with distinct CVD risk compared to those with MASLD, while the differences with the ALD population seem to be milder.

**Table 1:** Comparison of clinical and laboratory findings between patients with MetALD and those with MASLD

Higher in MASLD	Higher in ΜetALD
BMI	AST
HbA1c	ALT
Insulin	GGT
LDL-C	SBP
PLT	DBP
	HDL-C
	TG

MetALD: metabolic and alcohol related/associated liver disease; MASLD: metabolic dysfunction-associated steatotic liver disease, AST: aspartate transaminase; ALT: alanine aminotransferase; GGT: gamma-glutamyl transferase; eGFR: estimated glomerular filtration rate; PLT: platelet; SPB: systolic blood pressure; DBP: diastolic blood pressure; BMI: body mass index; HbA1c: hemoglobin A1C; LDL-C: low density lipoprotein cholesterol; HDL-C: high density lipoprotein cholesterol; TG: triglycerides

**Table 2:** Comparison of clinical and laboratory findings between patients with MetALD and those with ALD

Higher in ALD	Higher in ΜetALD
AST	BMI
ALT	LDL-C
GGT	
SBP	
DBP	

MetALD: metabolic and alcohol related/associated liver disease; ALD: alcohol-related liver disease; AST: aspartate transaminase; ALT: alanine aminotransferase; GGT: gamma-glutamyl transferase; SPB: systolic blood pressure; DBP: diastolic blood pressure; BMI: body mass index; LDL-C: low density lipoprotein cholesterol

There is still a lack of data regarding efficient MetALD patient identification, diagnosis and prognosis [82, 83]. In other words, we are not aware whether there is a considerable number of patients who fulfill the criteria for MetALD diagnosis, but we need to optimize our diagnostic tools and understand if they are characterized by different laboratory profiles that may affect its clinical outcomes compared to other SLD subtypes. Indeed, alcohol consumption tends to be underestimated in the relevant clinical studies, due to recall bias or the unwillingness of the patients to honestly self-report alcohol consumption. In this regard, we need indices to better estimate the alcohol consumption, such as phosphatidylethanol (PEth) [84]. Since PEth is a specific biomarker of recent alcohol consumption (up to 4 weeks), it has been proven to outperform indirect biomarkers like GGT, AST/ALT ratio, self-reported questionnaires and composite scores [85]. Recent data also suggest that PEth testing identified individuals originally classified as MASLD who may have been classified as MetALD or even ALD [86]. Finally, higher Peth levels were associated with increased risk of major adverse liver outcomes, even after adjusting for metabolic risk factors and fibrosis scores [87]. Consequently, its use may reduce significantly patient misclassification in clinical practice and may improve management and evaluation of prognosis in patients with SLD.

In this meta-analysis, several laboratory variables of MetALD showed statistical differences when compared with MASLD. Higher pooled mean levels of AST, ALT, and GGT in MetALD may imply more pronounced hepatocellular injury, potentially reflecting additive hepatic stress from alcohol exposure on top of metabolic dysfunction [8, 10]. Especially, elevated TGs and HDL-C and lower LDL-C in MetALD relative to MASLD patients may indicate distinct alterations in lipid metabolism related to increased alcohol intake [12]. Previous studies have shown that moderate alcohol consumption may increase HDL-C, but may raise TGs [11, 12]. These results contrast previous data reporting similar rates of dyslipidemia in patients with MASLD and MetALD [4]. On the contrary, in the subgroup analysis, among non-Asian patients, TGs were higher in MASLD compared to MetALD patients (Table S7). While higher TG levels in MetALD overall may reflect alcohol-induced hepatic de novo lipogenesis, the lower TG levels observed in non-Asian patients with MetALD could be attributed to different population characteristics such as BMI (higher obesity rates in non-Asian MASLD), dietary patterns or distinct genetic variants in alcohol-metabolizing enzymes that are associated with differences in circulating lipid profiles [88]. These subgroup findings should be interpreted cautiously, given the smaller number of studies and potential residual confounding. In any case, lipid parameters may have important clinical implications, since MetALD has been reportedly associated with higher CVD-related mortality, compared to the non- SLD population [4], but there is to date no data for a direct comparison between MASLD and MetALD regarding the risk of CVD morbidity and mortality. Thus, the distinct lipid equilibrium that characterizes MetALD patients and its association with CVD outcomes, compared to MASLD, needs further clarification. This is important, since higher HDL-C and lower LDL-C shown in MetALD (vs. MASLD) have been associated with lower CVD risk, whereas higher TGs also shown in MetALD (vs. MASLD) have been associated with higher CVD risk.

An important consideration when interpreting the observed differences in lipid profiles and blood pressure across SLD subtypes is the potential confounding effect of concomitant medication use. Across the studies included in this meta-analysis, reporting of medications use for concomitant metabolic diseases was inconsistent, and adjustment for pharmacologic treatment was performed only in a minority of these studies. Consequently, differences in lipid parameters—such as lower LDL-C and higher HDL-C in MetALD compared with MASLD—or modest differences in blood pressure may, at least in part, reflect variations in treatment intensity, healthcare access, or prescribing practices rather than pathophysiological differences alone.

Although BMI was modestly lower in MetALD compared with MASLD, this difference was small and may not possibly be clinically meaningful. Moreover, BMI does not accurately reflect abdominal adiposity and is affected by both subcutaneous adiposity and muscle mass; importantly, low muscle mass may also affect SLD and CVD risk [89, 90]. Visceral adiposity and sarcopenic obesity may be particularly relevant in MetALD, in which alcohol-related loss of muscle mass may coexist with excess ectopic fat deposition [91, 92]. Thus, obesity-related mechanisms remain highly relevant in MetALD, despite marginal differences in BMI, and may partly explain the adverse metabolic and cardiovascular profile observed in this population. Additionally, subgroup analyses demonstrated that non-Asian patients with MASLD had higher BMI values compared with those with MetALD, whereas BMI did not differ between MetALD and MASLD among Asian patients. This pattern aligns with evidence that MASLD in Asian populations occurs at lower BMI thresholds, likely reflecting differences in visceral adiposity and metabolic risk at lower absolute BMI levels [93]. As a result, BMI may underestimate metabolic burden in Asian compared with non-Asian populations. Obesity clinicians should systematically screen patients for SLD, including liver ultrasound, assessment of alcohol intake in addition to metabolic risk factors and consider incorporating liver enzymes and simple non-invasive fibrosis scores (e.g., FIB-4) into routine assessment. The potential role of objective alcohol biomarkers (PEth) appears promising [84] but requires further validation before its routine use in obesity clinical practice. From a therapeutic perspective, emerging pharmacologic strategies, such as glucagon-like peptide-1 (GLP-1) receptor agonists are emerging agents in patients with MetALD by improving weight, glycemic control, hepatic steatosis, inflammation and possibly fibrosis, although careful attention to the risk of sarcopenia and preservation of muscle mass is warranted, particularly in individuals with alcohol-related muscle loss [94, 95]. Finally, in patients with advanced liver fibrosis, a low threshold for referral to hepatology specialists should be maintained. Improved diagnostic tools beyond BMI, and targeted management strategies addressing both excess adiposity and alcohol intake are needed to optimize risk stratification and clinical outcomes in these patients.

It should be also mentioned that small changes in the levels of lipid parameters, such as a 10 mg/dL increase in TGs levels, have been associated with significant increase of the CVD risk [96], while higher TGs regardless of LDL-C levels are associated with increased atherosclerotic risk and endothelial dysfunction, even in cases of mild deviations from normal values [97]. Consequently, these lipid differences, although numerically small, may have a significant impact on CVD risk for these patients. Nevertheless, LDL-C, which appears to be higher in patients with MASLD (vs. MetALD) in our analysis, is regarded as the most robustly associated lipid parameter with cardiovascular morbidity and is regarded as main prognostic factor for CVD [98].

In terms of blood pressure, alcohol consumption is known to have a dose-dependent, linear association with it [99]; consequently, higher SBP and DBP values are expected in MetALD patients, compared to MASLD. Our analysis confirmed this hypothesis although the absolute differences were small. On the other hand, although the data regarding the effect of alcohol on BMI and HbA1c are mixed and affected by sex, amount/frequency of consumption and type of alcohol [100, 101], in this meta-analysis, patients with MetALD (vs. MASLD) had lower BMI and HbA1c. Similarly, higher eGFR and lower PLT in MetALD (vs. MASLD) may be associated with higher alcohol consumption in the former than the latter group; however, further studies are warranted to evaluate their clinical relevance.

Furthermore, in this meta-analysis, MetALD patients exhibited similar NFS and small differences in FIB-4 values compared to those with MASLD. Interestingly, in the subgroup analysis, FIB-4 values were higher in Asian patients with MetALD compared with MASLD, a pattern that was not observed in non-Asian populations. Although these findings should be interpreted cautiously, they may suggest greater susceptibility to alcohol-related liver injury among Asian individuals, potentially reflecting ethnic differences in alcohol metabolism or vulnerability to fibrosis [102]. It should be also mentioned that these observations were based on a limited number of studies. On the other hand, the reported higher FLI values in MetALD patients may be attributed to higher TG and GGT levels, which are both included in the equation of FLI [103]. In contrast, the absence of significant differences in LSM, and MRE suggests broadly comparable fibrosis burden between MetALD and MASLD, supporting the notion that alcohol-related mechanisms may initially cause biochemical alterations before translating into measurable differences in liver stiffness. Existing data indicate that in MetALD, synergistic metabolic–alcohol interactions initially promote hepatocellular injury, inflammation, and stellate cell activation, while progression to advanced fibrosis may depend on additional modifiers such as genetic susceptibility, gut–liver axis dysfunction, and adipose tissue inflammation [104]. From a clinical perspective, these findings highlight the need for cautious interpretation of composite NITs in MetALD, as alcohol-related effects on individual test components may increase estimates without reflecting true fibrosis severity. Based on the above, the optimal cut-off values for MetALD patients are still under investigation [105]. Perhaps sequential testing, i.e., using a combination of different tests may result in better accuracy in diagnosis and staging of MetALD, as is considered for MASLD [105].

When compared to patients with ALD, those with MetALD had lower AST, ALT and GGT levels, supporting the notion that the effect of alcohol on the liver is associated to the amount of alcohol consumption [106]. Consequently, heavier drinking seems to have a more profound effect, at least on LFTs. BMI and LDL-C levels were found to be statistically higher in MetALD patients, findings that warrant further research. Other metabolic parameters (e.g., HbA1c, HOMA-IR, total cholesterol) were similar, suggesting that perhaps these populations share common metabolic characteristics [107]. One possible explanation for the comparable metabolic profiles observed in MetALD and ALD is that, beyond a certain level of alcohol intake, the additional contribution of metabolic abnormalities may reach a “plateau”, with alcohol-related effects exerting a more dominant influence [108]. Excessive alcohol intake has been linked to a sustained rise in adiposity and intrahepatic accumulation of lipids, raising LDL-C and TG levels, thereby contributing to atherosclerosis, hypertension and increased CVD risk [109–110]. Recent data also show that patients with ALD are at increased risk for T2DM [111]. As a result, excessive alcohol consumption often coexists and is a risk factor for the development of the metabolic syndrome [4], which is usually underestimated in patients with ALD. However, it should be also considered that milder differences between the two groups in terms of metabolic characteristics could be attributed to misclassification of patients, when quantification of alcohol consumption is based on self-reported *questionnaires (recall bias).* Nevertheless, cardiometabolic parameters may require evaluation in patients with ALD in clinical practice, since they may contribute to the CVD risk of this population. Although this remains to be specifically shown, if validated, this may have important implications for the diagnosis and management of patients with ALD.

Conclusively, the observed laboratory and metabolic differences across MetALD, MASLD, and ALD support a continuum model of SLD in which the relative contributions of metabolic dysfunction and alcohol exposure shape distinct, yet overlapping, phenotypes. Compared with MASLD, MetALD is characterized by modestly higher transaminases and GGT alongside a lipid profile marked by higher TG and HDL-C but lower LDL-C, suggesting additive hepatocellular injury and alcohol-driven alterations in lipid metabolism. Experimental and clinical data indicate that alcohol exposure amplifies hepatic oxidative stress, mitochondrial dysfunction, inflammatory signaling in hepatic tissue and gut–liver and adipose–liver crosstalk, thereby accelerating steatohepatitis especially when metabolic dysfunction coexists, thereby causing liver injury beyond that observed in MASLD alone [5, 6, 11, 12, 104]. Conversely, the largely overlapping metabolic profile between MetALD and ALD, combined with lower liver enzyme levels in MetALD, suggests that once alcohol intake exceeds a critical threshold, alcohol-related hepatotoxic mechanisms may dominate [10, 106]. In this context, MetALD appears to occupy an intermediate pathophysiological position, reflecting both quantitatively lower alcohol-related injury than ALD and qualitatively distinct metabolic–alcohol interactions compared with MASLD.

### Limitations

Overall, the absence of statistically significant small-study effects and the consistency of the findings across sensitivity analyses support the robustness and reliability of our conclusions. However, this meta-analysis has several limitations. First, all included studies were observational, which may bear the risk of relevant biases. Several domains of bias inherent to observational studies may influence the interpretation of laboratory and metabolic outcomes; however, most of them characterize primarily limitations of the included studies and not methodological limitations of this meta-analysis. Selection bias may arise from the inclusion of health-checkup populations or referral-based cohorts, which may not be representative of the general SLD population. Recall bias is particularly relevant for alcohol consumption, as most studies relied on patient self-reporting consumption, which may be underreported, thus leading to misclassification. This may have partly distorted the results between MetALD and MASLD or ALD groups. Residual confounding is also likely, given inconsistent reporting of use of concomitant medications, lifestyle (diet and exercise), and relevant comorbidities, which may have affected lipid and glycemic profiles, blood pressure and renal function. Sensitivity analyses excluding studies being possibly at higher risk of bias yielded consistent results, thus partly supporting the robustness of the observed associations. Second, the diagnosis of MetALD, MASLD, and ALD varied across studies; in most of them, alcohol consumption was based on self-reported questionnaires, which are prone to recall and record biases. Third, substantial heterogeneity was observed in several analyses, likely reflecting differences in geographic regions, ethnic backgrounds, and diagnostic methods. To address potential sources of heterogeneity and reduce bias from confounding inherent in observational designs, we used a random-effects model for all pooled analyses, and we performed subgroup analyses by ethnicity and diagnostic modality. However, other important contributors, such as method of alcohol and fibrosis assessment, could not be reliably examined due to limited and inconsistent reporting. Consequently, part of the observed heterogeneity remains unexplained. Moreover, several differences in terms of laboratory findings, although statistically significant, need further research to show their clinical relevance. Additionally, residual confounding due to un-reported medication use represents another limitation. The outcomes of interest were not adjusted for treatment with lipid-lowering, antihypertensive, or anti-diabetic medications, which may have influenced the observed differences between groups. As such, some statistically significant differences may reflect treatment effects rather than pathophysiologic differences between SLD subtypes. Finally, another important limitation of the present study is the lack of availability of multivariable-adjusted effect estimates. In most cases, studies reported only unadjusted group-level summary statistics (means and standard deviations) rather than multivariable-adjusted effect estimates that could be pooled in a separate meta-analysis. Moreover, when adjusted models were reported, different covariates were included in the models of different studies (e.g. age, sex, BMI, diabetes, hypertension, medication use, fibrosis stage), precluding meaningful quantitative synthesis of adjusted estimates.

Future studies on MetALD should be prospectively designed with standardized assessment of alcohol intake, ideally combining validated questionnaires with objective biomarkers such as PEth to reduce misclassification. Systematic reporting and adjustment for concomitant metabolic medications are needed to limit residual confounding. In addition, characterization of metabolic burden should extend beyond BMI to include measures of visceral adiposity and body composition. The use of universal non-invasive fibrosis tools specifically adjusted for SLD would also facilitate more robust comparisons across studies. Finally, longitudinal studies directly comparing liver-related and cardiovascular outcomes across MASLD, MetALD, and ALD are essential to determine the clinical implications of each distinct SLD subtype.

## Conclusions

In conclusion, MetALD is a newly introduced clinical entity within the SLD spectrum, which may be characterized by different laboratory and metabolic features when compared to MASLD. On the contrary, apart from the LFTs, there is certain similarity between MetALD and ALD. These findings to date should be interpreted as associations rather than evidence of causation. Moreover, despite modest differences in BMI compared with MASLD, obesity-related mechanisms remain central to MetALD pathogenesis, underscoring the need to address excess adiposity alongside alcohol exposure in risk stratification and management. Further prospective studies, as well as diagnostic accuracy studies are needed to refine diagnostic criteria for MetALD and to possibly develop targeted management strategies that address both metabolic and alcohol-related risk factors.

## Key References

• Rinella ME et al. A multisociety Delphi consensus statement on new fatty liver disease nomenclature. Hepatology. 2023.
  - ◦ This consensus document established the new nomenclature for MASLD/MetALD, providing the definitions that are essential for interpreting and standardizing research on steatotic liver disease.
• EASL–EASD–EASO Clinical Practice Guidelines on MASLD. J Hepatol. 2024.
  - ◦ These multidisciplinary clinical guidelines outline evidence-based recommendations for the diagnosis, staging, and management of MASLD and related steatotic entities. They serve as the primary reference for clinicians treating patients with steatotic liver disease.
• Tampaki M et al. Prevalence, characteristics, and outcomes of MetALD: a systematic review & meta-analysis. Metabolism. 2025.
  - ◦ As one of the first comprehensive analyses focused specifically on MetALD, this study provides significant epidemiologic and clinical insights. It helped define the burden, phenotypic features and possible prognosis of MetALD.
• Marti-Aguado D et al. Low-to-moderate alcohol consumption and fibrosis in MASLD. J Hepatol. 2024.
  - ◦ This study demonstrates that even modest alcohol intake worsens hepatic fibrosis in MASLD, highlighting the synergistic risk in MetALD. It provides strong clinical evidence for reconsidering alcohol guidance among metabolic risk populations.
• Israelsen M et al. Validation of the new SLD nomenclature in individuals with excessive alcohol intake. Lancet Gastroenterol Hepatol. 2024.
  - ◦ This prospective cohort study validates the MASLD/MetALD/ALD categories in real-world patients with high alcohol intake. It supports the clinical utility of the new nomenclature and provides outcome-based justification for the updated classification.
• Vaz J et al. Phosphatidylethanol distinguishes SLD subgroups. J Hepatol. 2025.
  - ◦ This study shows that PEth—a highly specific alcohol biomarker—can accurately subclassify SLD phenotypes and predict liver outcomes. Its findings have major implications for improving diagnosis of MetALD and overcoming bias on self-reported alcohol intake.

## Supplementary Information

Below is the link to the electronic supplementary material.

Supplementary Material 1

Supplementary Material 2

## Data Availability

No datasets were generated or analysed during the current study.
