# Supplementary material for: Distinct Laboratory and Clinical Features of Metabolic and Alcohol-Related Liver Disease (MetALD): A Systematic Review and Meta-Analysis
Source: Curr Obes Rep. 2026 Mar 6;15(1):19. doi: 10.1007/s13679-026-00696-6 (PMC12966208; doi:10.1007/s13679-026-00696-6)
Supplement: Supplementary file 1 — Supplementary Material 1 [file 13679_2026_696_MOESM1_ESM.docx]

**Supplementary material**

**Table of contents**

| **Section/topic** | **Reported on page #** |
| --- | --- |
| Supplementary Figure S1 | 3 |
| Supplementary Figure S2 | 4 |
| Supplementary Figure S3 | 5 |
| Supplementary Figure S4 | 6 |
| Supplementary Figure S5 | 7 |
| Supplementary Figure S6 | 8 |
| Supplementary Figure S7 | 9 |
| Supplementary Figure S8 | 10 |
| Supplementary Figure S9 | 11 |
| Supplementary Figure S10 | 12 |
| Supplementary Figure S11 | 12 |
| Supplementary Figure S12 | 13 |
| Supplementary Figure S13 | 13 |
| Subgroup analysis Supplementary Figures S14-S130 | 14-130 |
| Sensitivity Analysis Supplementary Figures S131-S165 | 131-165 |
| Publication bias Supplementary Figures S166-S189 | 166-189 |
| Supplementary Table 1 | 190-206 |
| Supplementary Table 2 | 207 |
| Supplementary Table 3 | 208 |
| Supplementary Table 4 | 209 |

| Subgroup analysis Supplementary Tables 5-16 | 210-221 |
| --- | --- |
| Sensitivity analysis Supplementary Tables 17-20 | 222-225 |

**Supplementary Figure S1**. Forest plot of studies comparing the pooled mean total cholesterol levels between MetALD and MASLD patients.

.

*MetALD: metabolic and alcohol related/associated liver disease; MASLD: metabolic dysfunction-associated steatotic liver disease.*

**Supplementary Figure S2**. Forest plot of studies comparing the pooled mean fasting glucose levels between MetALD and MASLD patients.

*
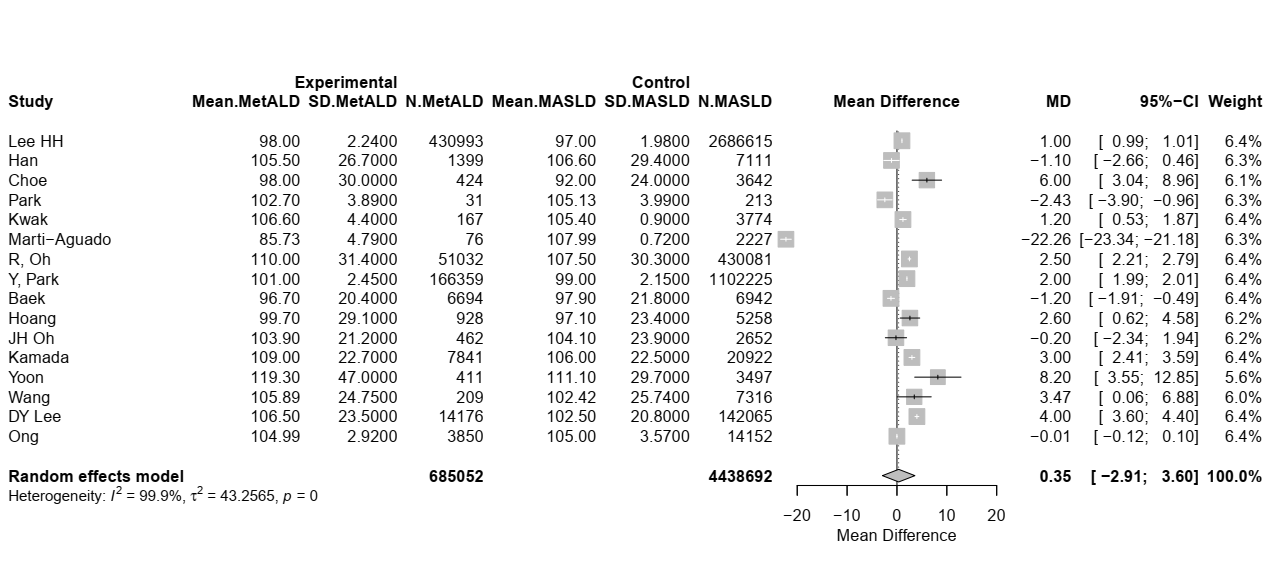
*

*MetALD: metabolic and alcohol related/associated liver disease; MASLD: metabolic dysfunction-associated steatotic liver disease.*

**Supplementary Figure S3**. Forest plot of studies comparing the pooled mean total Homeostatic Model Assessment for Insulin Resistance (HOMA-IR) between MetALD and MASLD patients.

*MetALD: metabolic and alcohol related/associated liver disease; MASLD: metabolic dysfunction-associated steatotic liver disease.*

**Supplementary Figure S4**. Forest plot of studies comparing the pooled mean waist circumference (WC) between MetALD and MASLD patients.


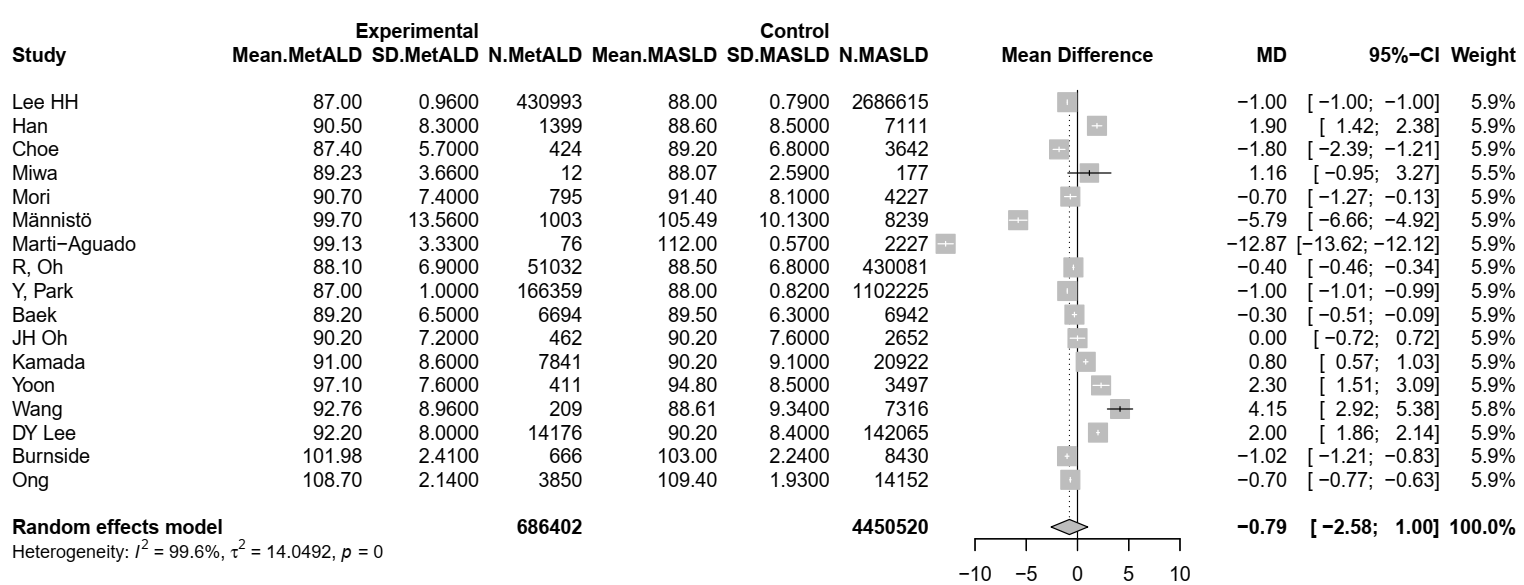


*MetALD: metabolic and alcohol related/associated liver disease; MASLD: metabolic dysfunction-associated steatotic liver disease.*

**Supplementary Figure S5**. Forest plot of studies comparing the pooled mean **a.** NAFLD fibrosis score (NFS), **b.** liver stiffness measurements (LSM), **c.** magnetic resonance elastography (MRE) measurements between MetALD and MASLD patients.


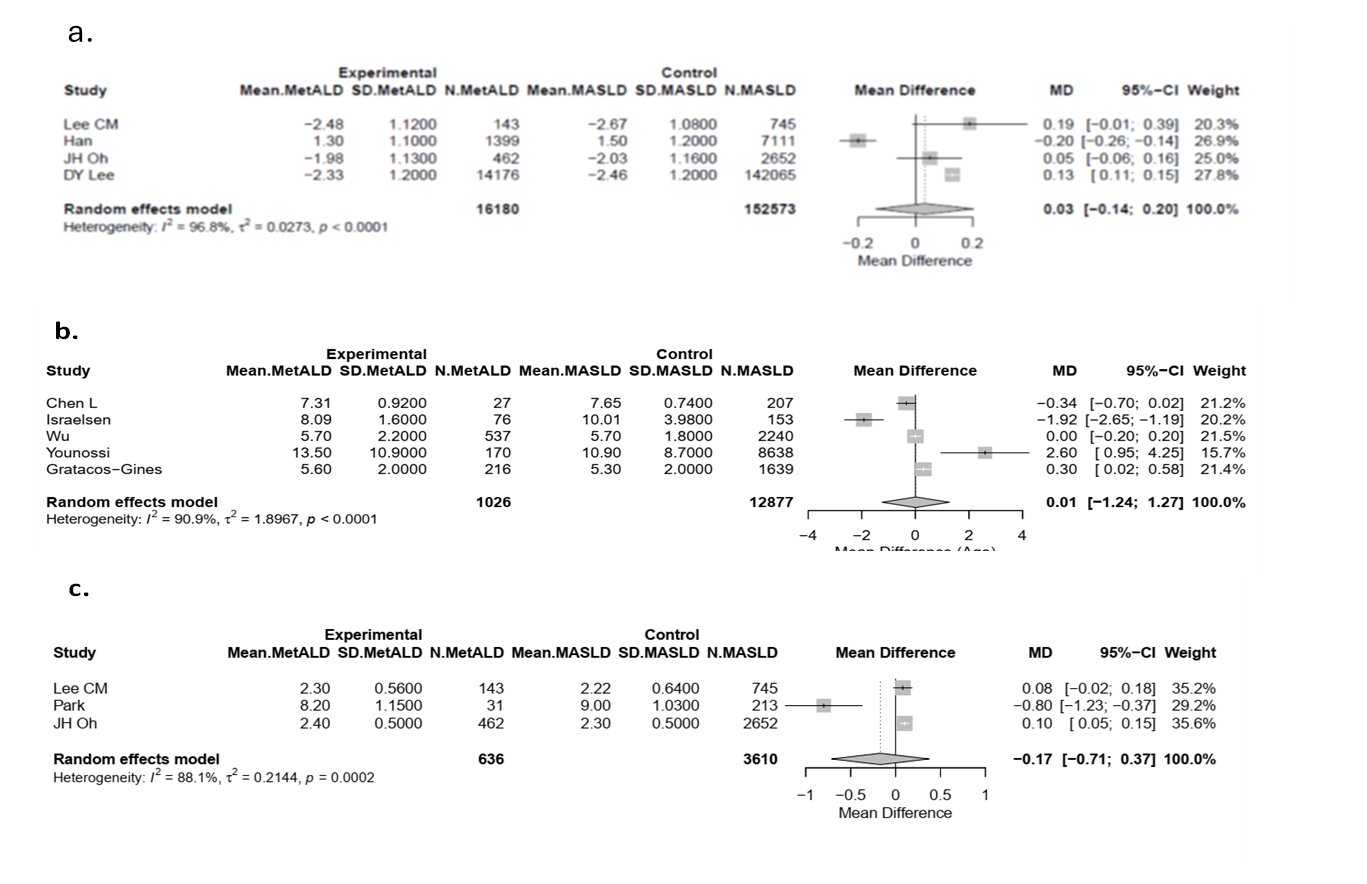


*MetALD: metabolic and alcohol related/associated liver disease; MASLD: metabolic dysfunction-associated steatotic liver disease.*

**Supplementary Figure S6**. Forest plot of studies comparing the pooled mean age between MetALD and ALD patients.


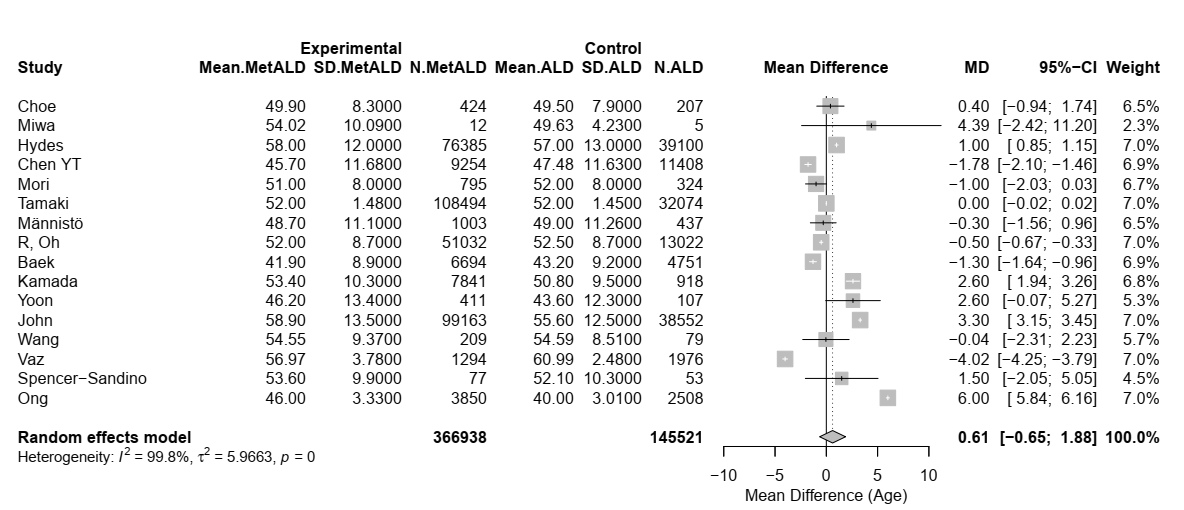


***Abbreviations****:* *MetALD: metabolic and alcohol related/associated liver disease; ALD: alcohol-related liver disease.*

**Supplementary Figure S7.** Forest plot of studies comparing the pooled mean levels of **a.** hemoglobin A1C (HbA1C) levels and **b.** fasting glucose levels between MetALD and ALD patients.


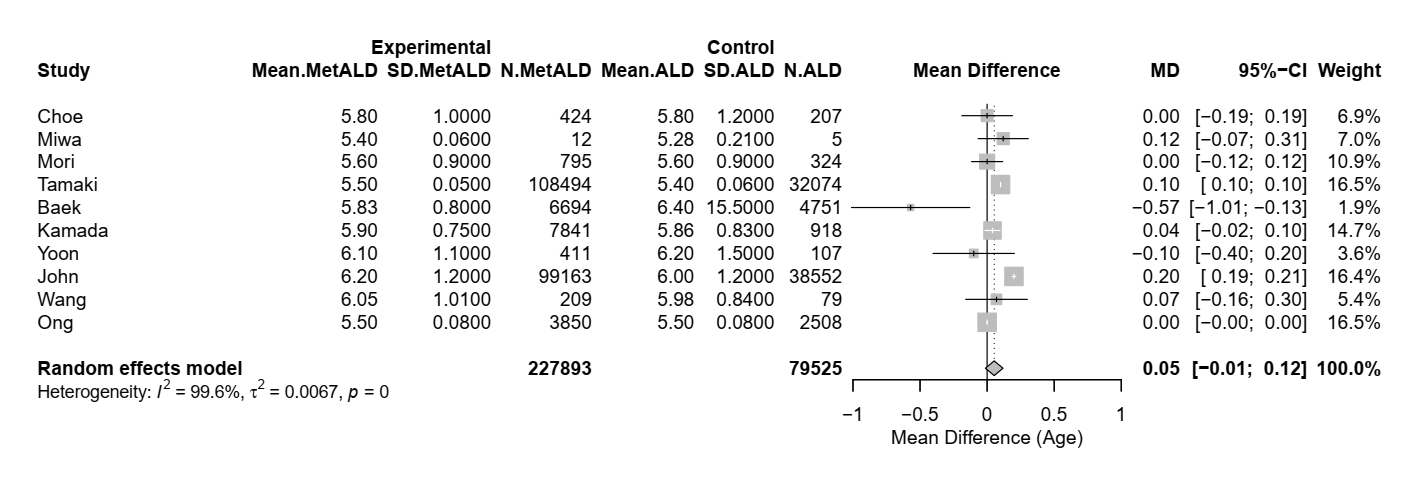


**a.**


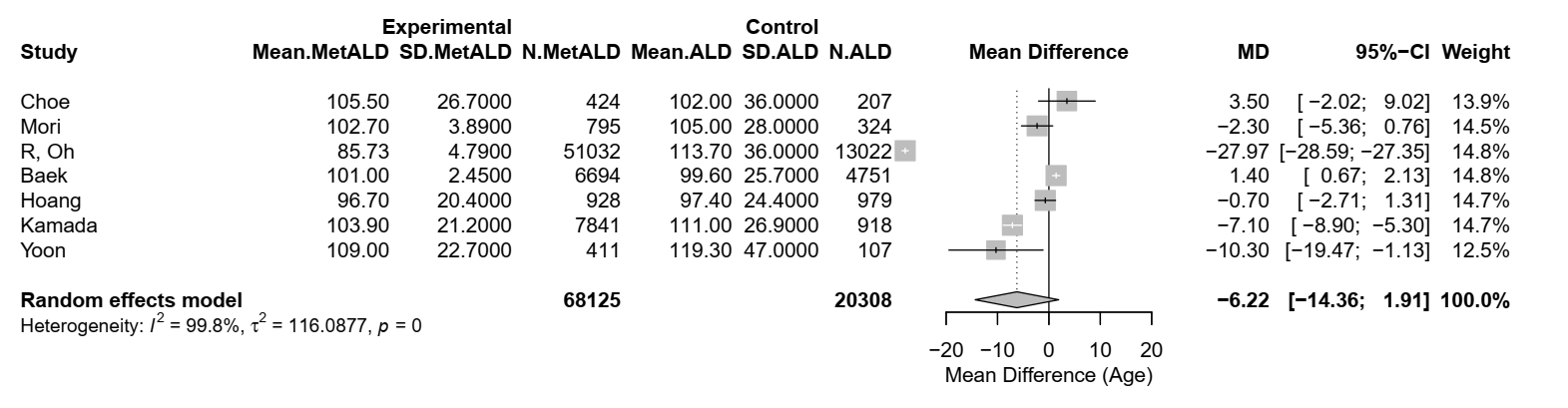


**b.**

***Abbreviations****: MetALD: metabolic and alcohol related/associated liver disease; ALD: alcohol-related liver disease.*

**Supplementary Figure S8.** Forest plot of studies comparing the pooled mean levels of **a.** total cholesterol **b.** high density lipoprotein (HDL) and **c.** triglycerides (TG) between MetALD and ALD patients.


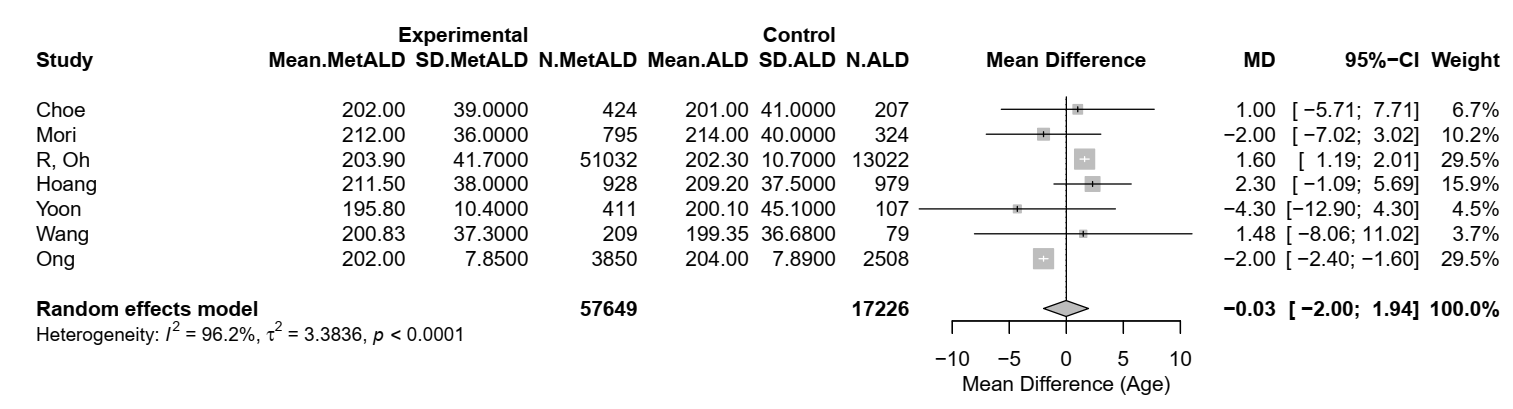

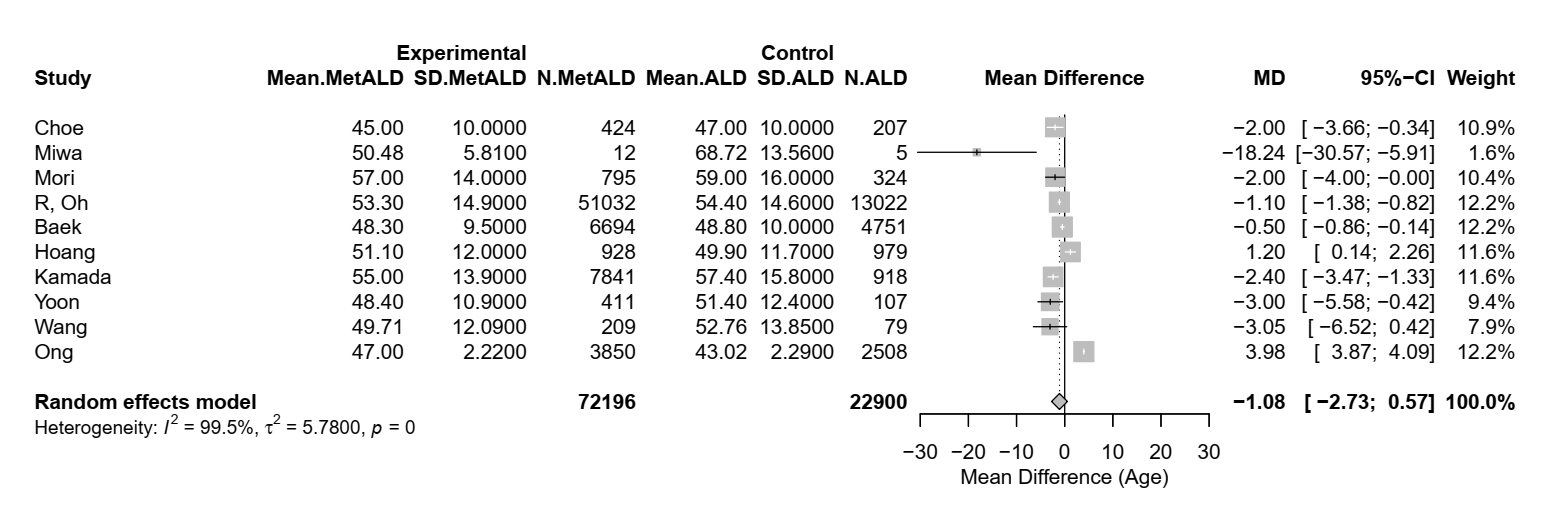

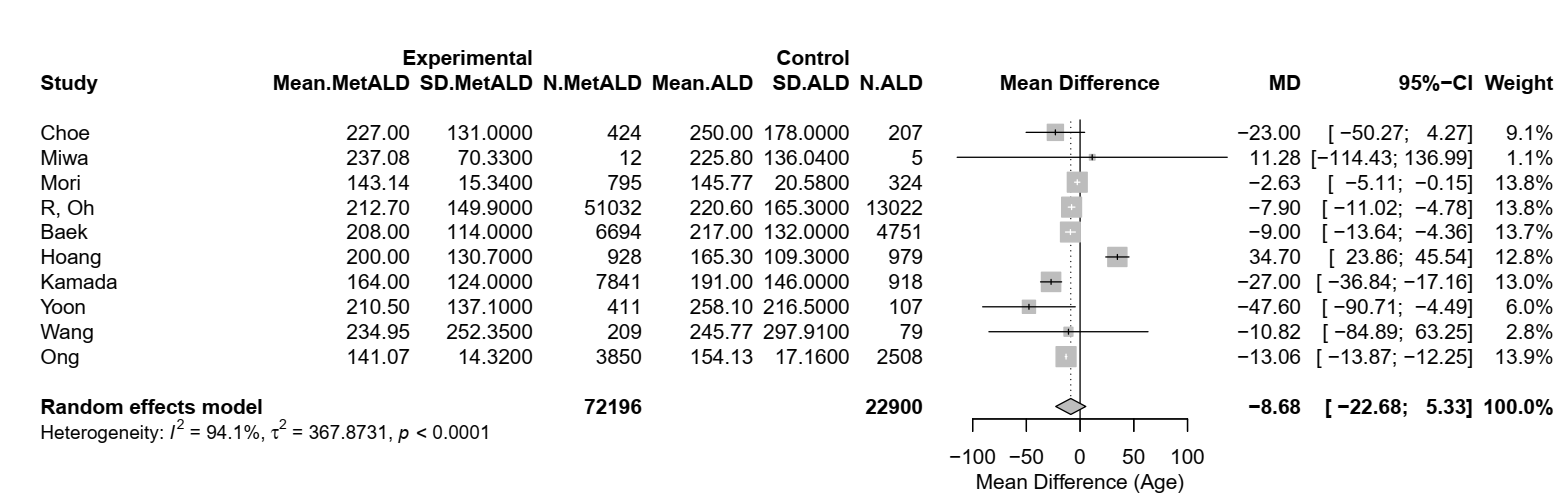


**b.**

**c.**

***Abbreviations****: MetALD: metabolic and alcohol related/associated liver disease; ALD: alcohol-related liver disease.*

**Supplementary Figure S9.** Forest plot of studies comparing the pooled mean total Homeostatic Model Assessment for Insulin Resistance (HOMA-IR) between MetALD and ALD patients.

***Abbreviations****: MetALD: metabolic and alcohol related/associated liver disease; ALD: alcohol-related liver disease.*

**Supplementary Figure S10.** Forest plot of studies comparing the pooled mean waist circumference (WC) between MetALD and ALD patients.

***Abbreviations****: MetALD: metabolic and alcohol related/associated liver disease; ALD: alcohol-related liver disease.*

**Supplementary Figure S11**. Forest plot of studies comparing the pooled mean levels of glomerular filtration rate (GFR) between MetALD and ALD patients.


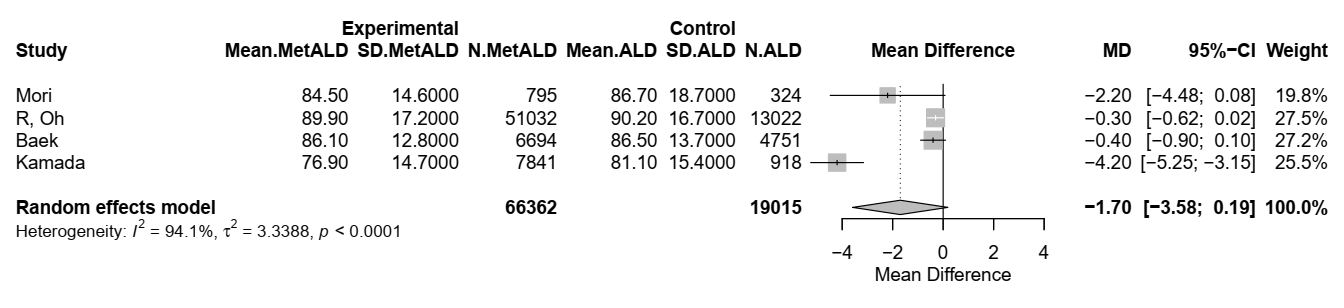


***Abbreviations****: MetALD: metabolic and alcohol related/associated liver disease; ALD:**alcohol-related liver disease.*

**Supplementary Figure S12.** Forest plot of studies comparing the pooled mean levels of platelet (PLT) count between MetALD and ALD patients.


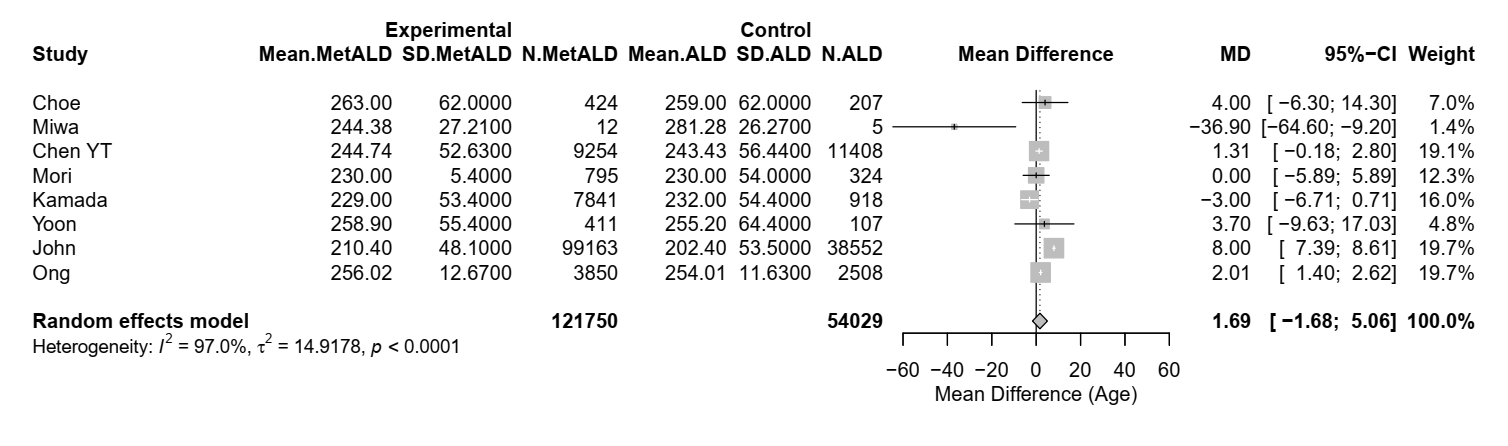


***Abbreviations****: MetALD: metabolic and alcohol related/associated liver disease; ALD:alcohol-related liver disease.*

**Supplementary Figure S13.** Forest plot of studies comparing the pooled mean Fibrosis-4 (FIB-4) score between MetALD and ALD patients.

***Abbreviations****: MetALD: metabolic and alcohol related/associated liver disease; ALD: alcohol-related liver disease.*

**Supplementary Figure S14**. Forest plot of studies comparing the age of Αsian MetALD patients compared to those with MASLD.


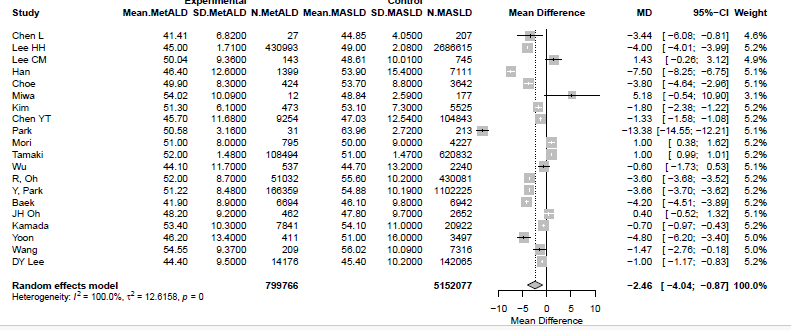


*MetALD: metabolic and alcohol related/associated liver disease; MASLD: metabolic dysfunction-associated steatotic liver disease.*

**Supplementary Figure S15**. Forest plot of studies comparing the pooled mean aspartate aminotransferase (AST) levels of Αsian MetALD patients compared to those with MASLD.


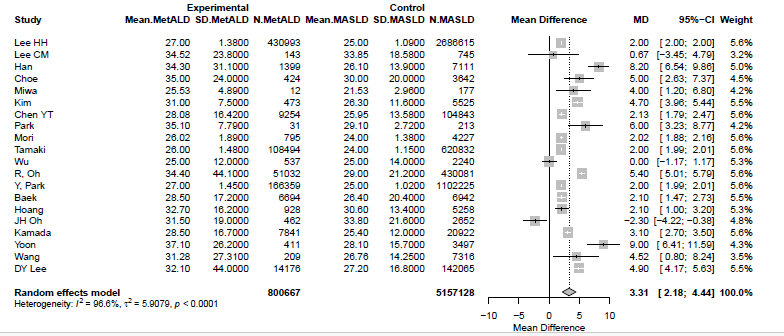


*MetALD: metabolic and alcohol related/associated liver disease; MASLD: metabolic dysfunction-associated steatotic liver disease.*

**Supplementary Figure S16**. Forest plot of studies comparing the pooled mean alanine aminotransferase (ALT) levels of Αsian MetALD patients compared to those with MASLD.


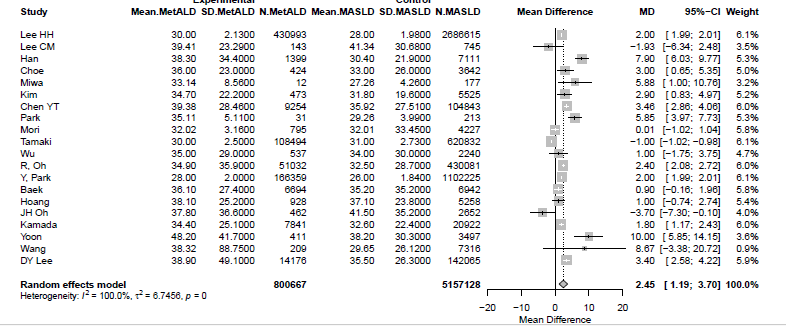


*MetALD: metabolic and alcohol related/associated liver disease; MASLD: metabolic dysfunction-associated steatotic liver disease.*

**Supplementary Figure S17**. Forest plot of studies comparing the pooled mean Gamma-Glutamyl Transferase (GGT) levels of Αsian MetALD patients compared to those with MASLD.


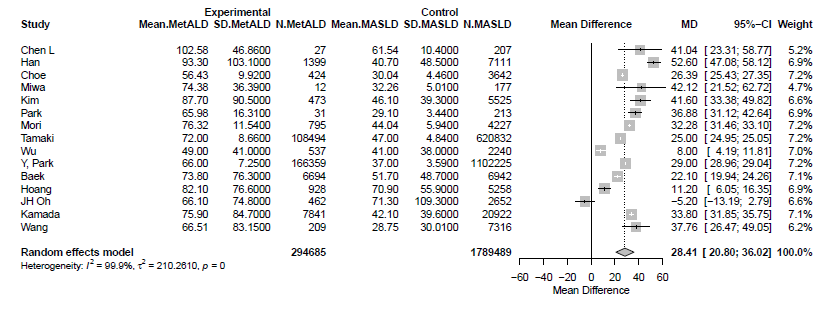


*MetALD: metabolic and alcohol related/associated liver disease; MASLD: metabolic dysfunction-associated steatotic liver disease.*

**Supplementary Figure S18**. Forest plot of studies comparing the pooled mean platelet count (PLT) of Αsian MetALD patients compared to those with MASLD.


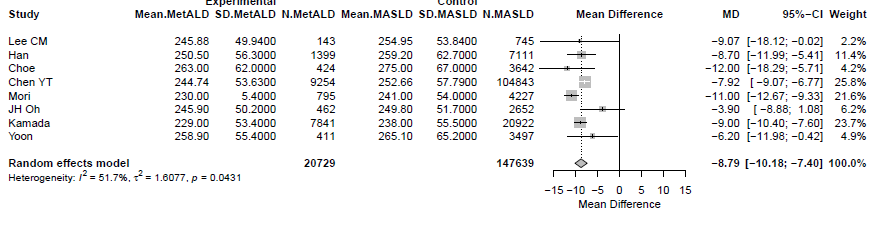


*MetALD: metabolic and alcohol related/associated liver disease; MASLD: metabolic dysfunction-associated steatotic liver disease.*

**Supplementary Figure S19**. Forest plot of studies comparing the estimated glomerular filtration rate (eGFR) of Αsian MetALD patients compared to those with MASLD.


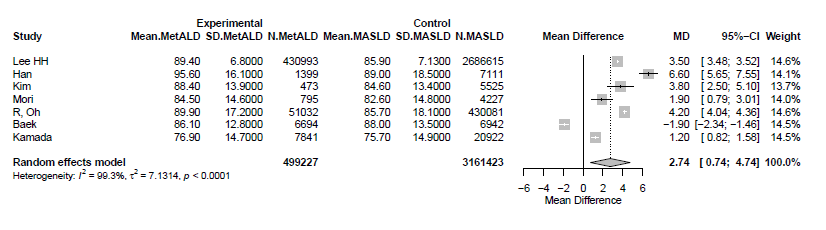


*MetALD: metabolic and alcohol related/associated liver disease; MASLD: metabolic dysfunction-associated steatotic liver disease.*

**Supplementary Figure S20**. Forest plot of studies comparing the pooled mean systolic blood pressure (SBP) levels of Αsian MetALD patients compared to those with MASLD.


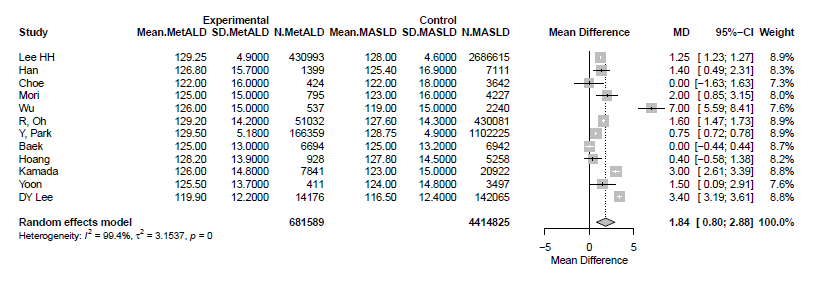


*MetALD: metabolic and alcohol related/associated liver disease; MASLD: metabolic dysfunction-associated steatotic liver disease.*

**Supplementary Figure S21**. Forest plot of studies comparing the pooled mean diastolic blood pressure (DBP) levels of Αsian MetALD patients compared to those with MASLD.


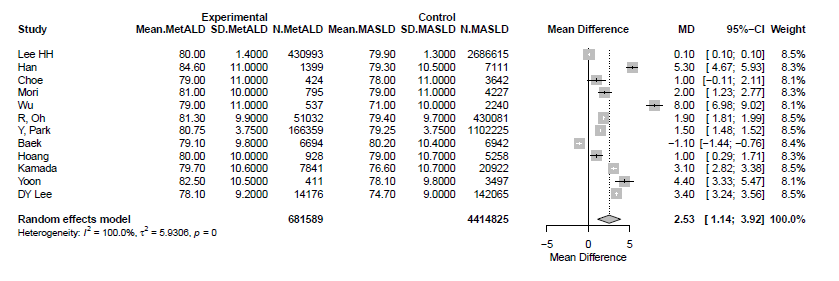


*MetALD: metabolic and alcohol related/associated liver disease; MASLD: metabolic dysfunction-associated steatotic liver disease.*

**Supplementary Figure S22**. Forest plot of studies comparing the pooled mean high-density cholesterol (HDL-C) levels of Αsian MetALD patients compared to those with MASLD.


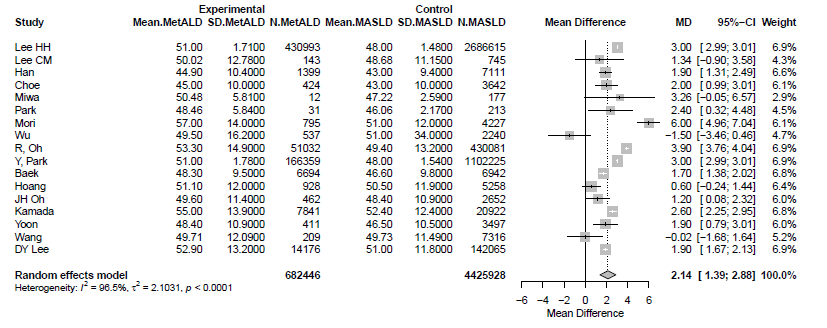


*MetALD: metabolic and alcohol related/associated liver disease; MASLD: metabolic dysfunction-associated steatotic liver disease.*

**Supplementary Figure S23**. Forest plot of studies comparing the pooled mean low-density cholesterol (LDL-C) levels of Αsian MetALD patients compared to those with MASLD.


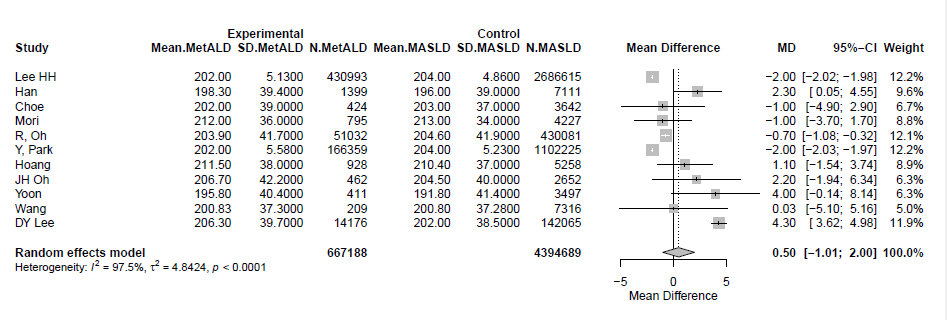


*MetALD: metabolic and alcohol related/associated liver disease; MASLD: metabolic dysfunction-associated steatotic liver disease.*

**Supplementary Figure S24**. Forest plot of studies comparing the pooled mean triglyceride (TG) levels of Αsian MetALD patients compared to those with MASLD.

**
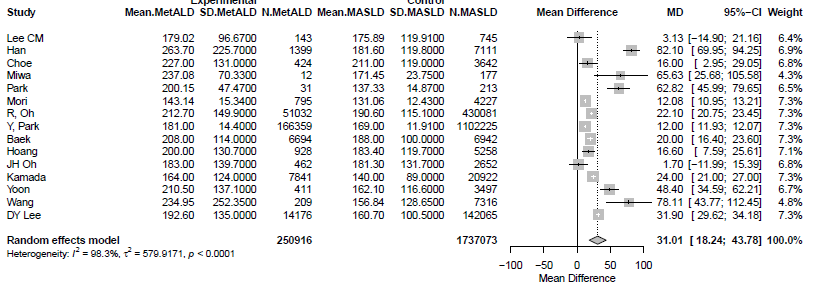
**

*MetALD: metabolic and alcohol related/associated liver disease; MASLD: metabolic dysfunction-associated steatotic liver disease.*

**Supplementary Figure S25**. Forest plot of studies comparing the pooled mean total cholesterol levels of Αsian MetALD patients compared to those with MASLD.

**
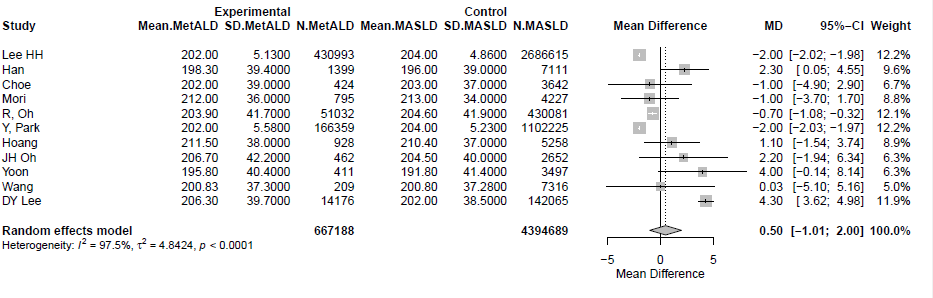
**

*MetALD: metabolic and alcohol related/associated liver disease; MASLD: metabolic dysfunction-associated steatotic liver disease.*

**Supplementary Figure S26**. Forest plot of studies comparing the pooled mean fasting glucose levels of Αsian MetALD patients compared to those with MASLD.


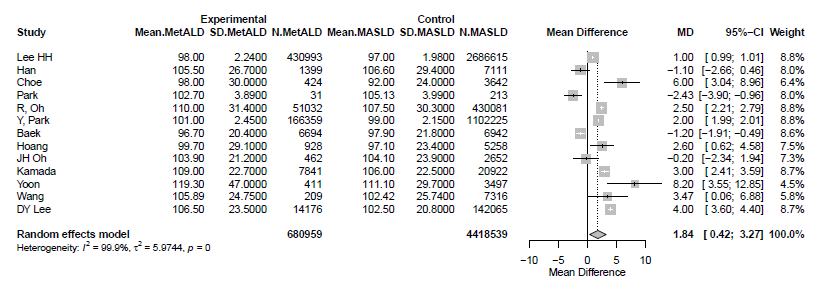


*MetALD: metabolic and alcohol related/associated liver disease; MASLD: metabolic dysfunction-associated steatotic liver disease.*

**Supplementary Figure S27**. Forest plot of studies comparing the pooled mean glycated hemoglobin A1C (Hba1c) levels of Αsian MetALD patients compared to those with MASLD.


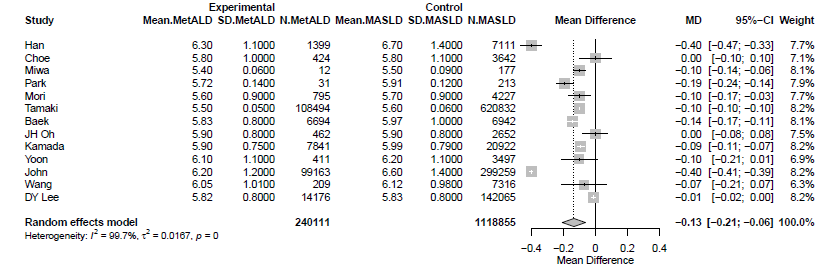


*MetALD: metabolic and alcohol related/associated liver disease; MASLD: metabolic dysfunction-associated steatotic liver disease.*

**Supplementary Figure S28**. Forest plot of studies comparing the body mass index (BMI) of Αsian MetALD patients compared to those with MASLD.

**
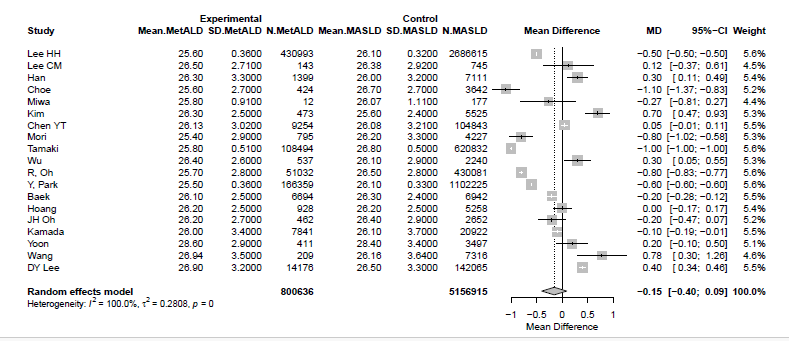
**

*MetALD: metabolic and alcohol related/associated liver disease; MASLD: metabolic dysfunction-associated steatotic liver disease.*

**Supplementary Figure S29**. Forest plot of studies comparing the waist circumference (WC) of Αsian MetALD patients compared to those with MASLD.


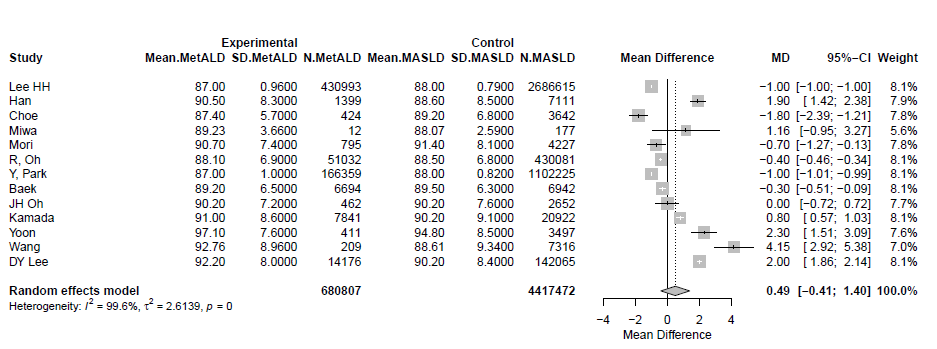


*MetALD: metabolic and alcohol related/associated liver disease; MASLD: metabolic dysfunction-associated steatotic liver disease.*

**Supplementary Figure S30**. Forest plot of studies comparing the Homeostatic Model Assessment for Insulin Resistance (HOMA-IR) of Αsian MetALD patients compared to those with MASLD.

**
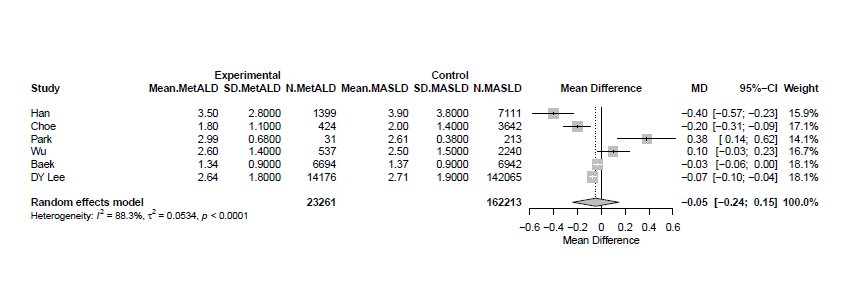
**

*MetALD: metabolic and alcohol related/associated liver disease; MASLD: metabolic dysfunction-associated steatotic liver disease.*

**Supplementary Figure S31**. Forest plot of studies comparing the pooled mean values of fibrosis-4 (FIB-4) score of Αsian MetALD patients compared to those with MASLD.

**
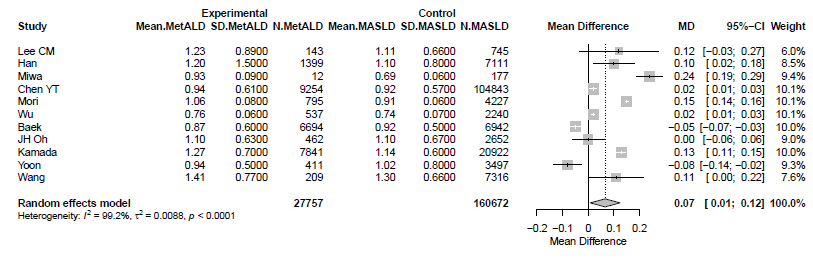
**

*MetALD: metabolic and alcohol related/associated liver disease; MASLD: metabolic dysfunction-associated steatotic liver disease.*

**Supplementary Figure S32**. Forest plot of studies comparing the age of non Αsian MetALD patients compared to those with MASLD.


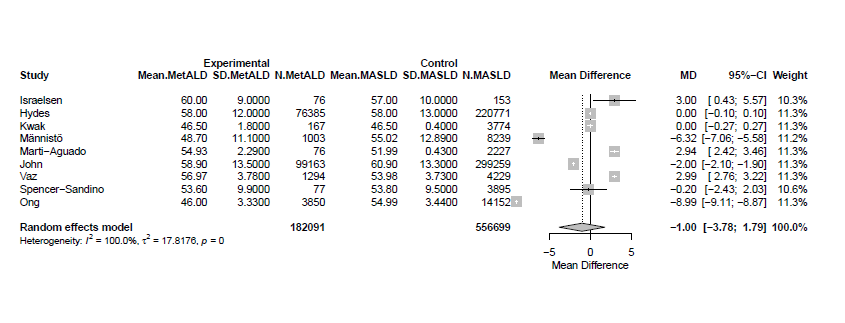


*MetALD: metabolic and alcohol related/associated liver disease; MASLD: metabolic dysfunction-associated steatotic liver disease.*

**Supplementary Figure S33**. Forest plot of studies comparing the pooled mean aspartate aminotransferase (AST) levels of non Αsian MetALD patients compared to those with MASLD.


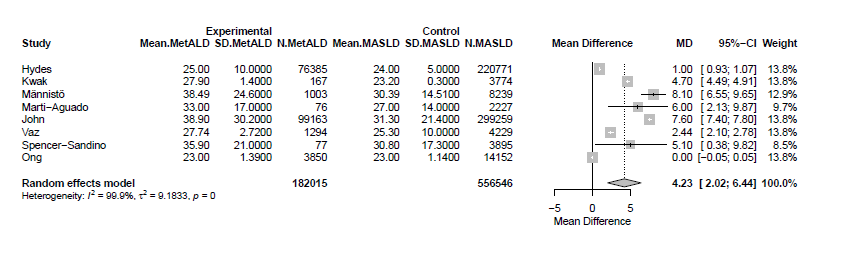


*MetALD: metabolic and alcohol related/associated liver disease; MASLD: metabolic dysfunction-associated steatotic liver disease.*

**Supplementary Figure S34**. Forest plot of studies comparing the pooled mean alanine aminotransferase (ALT) levels of non Αsian MetALD patients compared to those with MASLD.


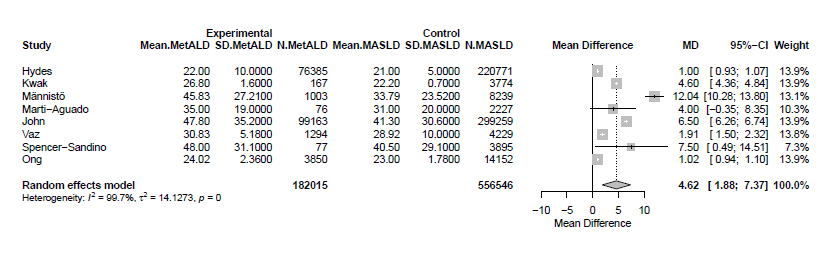


*MetALD: metabolic and alcohol related/associated liver disease; MASLD: metabolic dysfunction-associated steatotic liver disease.*

**Supplementary Figure S35**. Forest plot of studies comparing the pooled mean Gamma-Glutamyl Transferase (GGT) levels of non Αsian MetALD patients compared to those with MASLD.


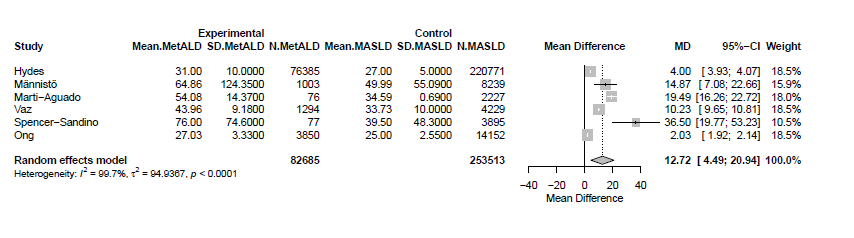


*MetALD: metabolic and alcohol related/associated liver disease; MASLD: metabolic dysfunction-associated steatotic liver disease.*

**Supplementary Figure S36**. Forest plot of studies comparing the pooled mean platelet count (PLT) of non Αsian MetALD patients compared to those with MASLD.


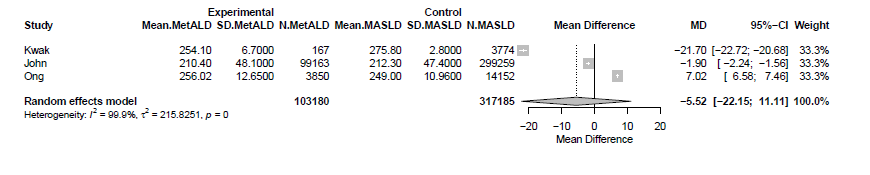


*MetALD: metabolic and alcohol related/associated liver disease; MASLD: metabolic dysfunction-associated steatotic liver disease.*

**Supplementary Figure S37**. Forest plot of studies comparing the pooled mean high-density cholesterol (HDL-C) levels of non Αsian MetALD patients compared to those with MASLD.


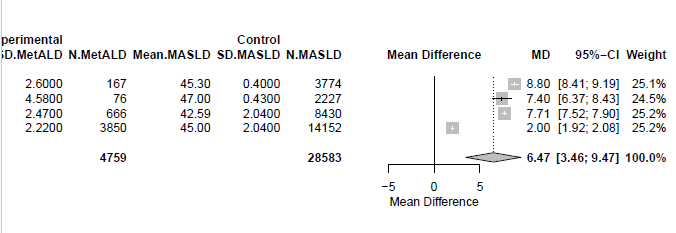


*MetALD: metabolic and alcohol related/associated liver disease; MASLD: metabolic dysfunction-associated steatotic liver disease.*

**Supplementary Figure S38**. Forest plot of studies comparing the pooled mean triglyceride (TG) levels of non Αsian MetALD patients compared to those with MASLD.

**
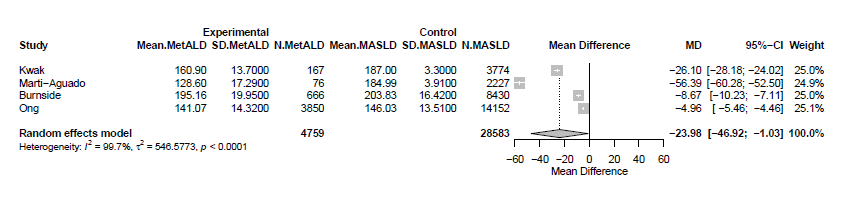
**

*MetALD: metabolic and alcohol related/associated liver disease; MASLD: metabolic dysfunction-associated steatotic liver disease.*

**Supplementary Figure S39**. Forest plot of studies comparing the pooled mean total cholesterol levels of non Αsian MetALD patients compared to those with MASLD.

**
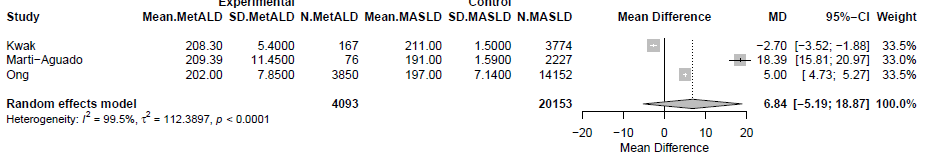
**

*MetALD: metabolic and alcohol related/associated liver disease; MASLD: metabolic dysfunction-associated steatotic liver disease.*

**Supplementary Figure S40**. Forest plot of studies comparing the pooled mean fasting glucose levels of non Αsian MetALD patients compared to those with MASLD.


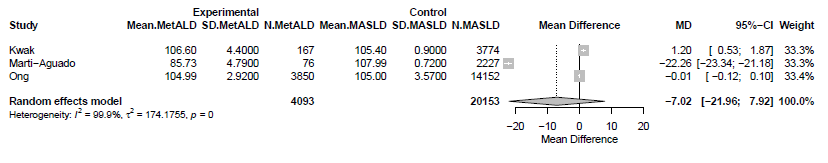


*MetALD: metabolic and alcohol related/associated liver disease; MASLD: metabolic dysfunction-associated steatotic liver disease.*

**Supplementary Figure S41**. Forest plot of studies comparing the pooled mean glycated hemoglobin A1C (Hba1c) levels of non Αsian MetALD patients compared to those with MASLD.


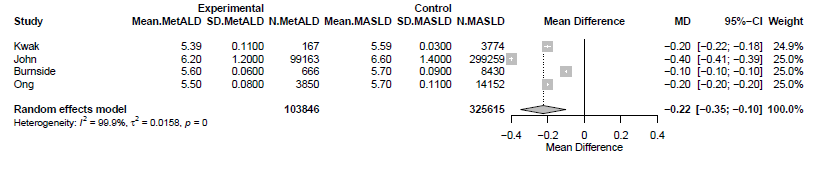


*MetALD: metabolic and alcohol related/associated liver disease; MASLD: metabolic dysfunction-associated steatotic liver disease.*

**Supplementary Figure S42**. Forest plot of studies comparing the body mass index (BMI) of non Αsian MetALD patients compared to those with MASLD.

**
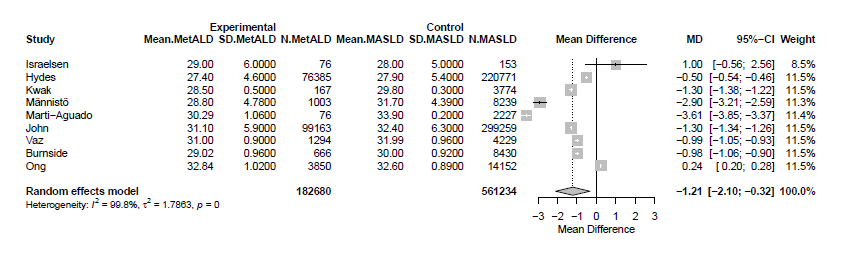
**

*MetALD: metabolic and alcohol related/associated liver disease; MASLD: metabolic dysfunction-associated steatotic liver disease.*

**Supplementary Figure S43**. Forest plot of studies comparing the waist circumference (WC) of non Αsian MetALD patients compared to those with MASLD.


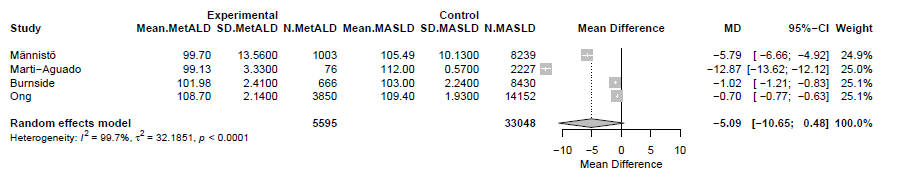


*MetALD: metabolic and alcohol related/associated liver disease; MASLD: metabolic dysfunction-associated steatotic liver disease.*

**Supplementary Figure S44**. Forest plot of studies comparing the pooled mean values of fibrosis-4 (FIB-4) score of non Αsian MetALD patients compared to those with MASLD.


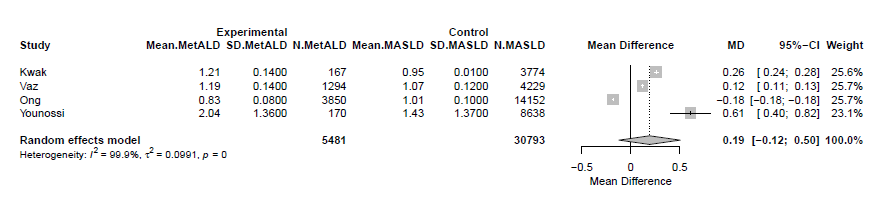


*MetALD: metabolic and alcohol related/associated liver disease; MASLD: metabolic dysfunction-associated steatotic liver disease.*

**Supplementary Figure S45**. Forest plot of studies comparing the pooled mean values of liver stiffness measurements (LSM) of non Αsian MetALD patients compared to those with MASLD.

**
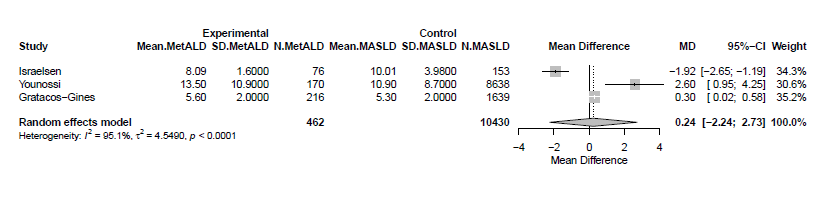
**

**Supplementary Figure S46**. Forest plot of studies comparing the age of MetALD patients compared to those with MASLD in studies using serum-based non-invasive tests for diagnosis of SLD.


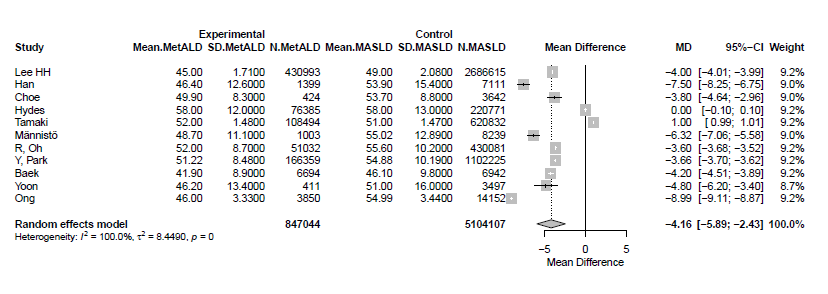


*MetALD: metabolic and alcohol related/associated liver disease; MASLD: metabolic dysfunction-associated steatotic liver disease; SLD: steatotic liver disease.*

**Supplementary Figure S47**. Forest plot of studies comparing the pooled mean aspartate aminotransferase (AST) levels of MetALD patients compared to those with MASLD in studies using serum-based non-invasive tests for diagnosis of SLD.


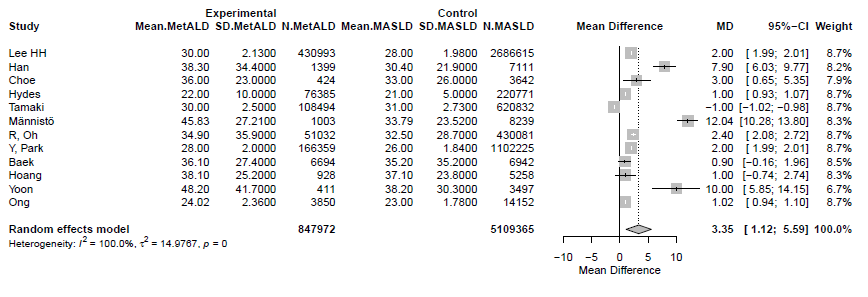


*MetALD: metabolic and alcohol related/associated liver disease; MASLD: metabolic dysfunction-associated steatotic liver disease; SLD: steatotic liver disease.*

**Supplementary Figure S48**. Forest plot of studies comparing the pooled mean alanine aminotransferase (ALT) levels of MetALD patients compared to those with MASLD in studies using serum-based non-invasive tests for diagnosis of SLD.


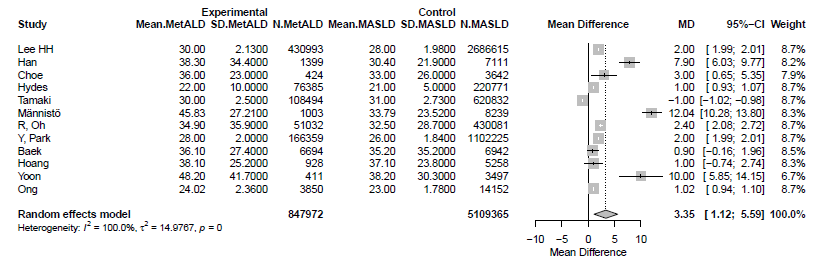


*MetALD: metabolic and alcohol related/associated liver disease; MASLD: metabolic dysfunction-associated steatotic liver disease; SLD: steatotic liver disease.*

**Supplementary Figure S49**. Forest plot of studies comparing the pooled mean Gamma-Glutamyl Transferase (GGT) levels of MetALD patients compared to those with MASLD in studies using serum-based non-invasive tests for diagnosis of SLD.


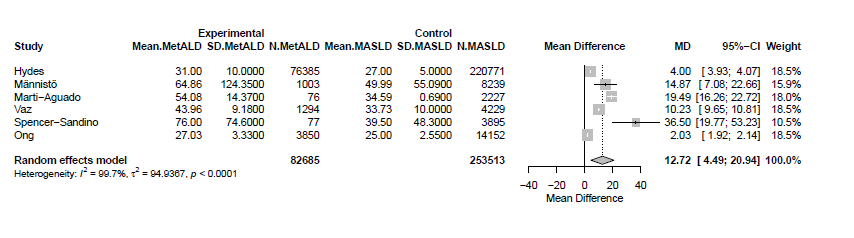


*MetALD: metabolic and alcohol related/associated liver disease; MASLD: metabolic dysfunction-associated steatotic liver disease; SLD:steatotic liver disease.*

**Supplementary Figure S50**. Forest plot of studies comparing the pooled mean platelet count (PLT) of MetALD patients compared to those with MASLD in studies using serum-based non-invasive tests for diagnosis of SLD.


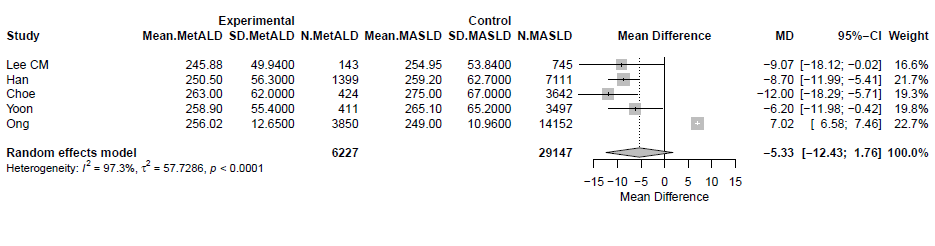


*MetALD: metabolic and alcohol related/associated liver disease; MASLD: metabolic dysfunction-associated steatotic liver disease; SLD: steatotic liver disease.*

**Supplementary Figure S51**. Forest plot of studies comparing the pooled mean systolic blood pressure (SBP) levels of MetALD patients compared to those with MASLD in studies using serum-based non-invasive tests for diagnosis of SLD.


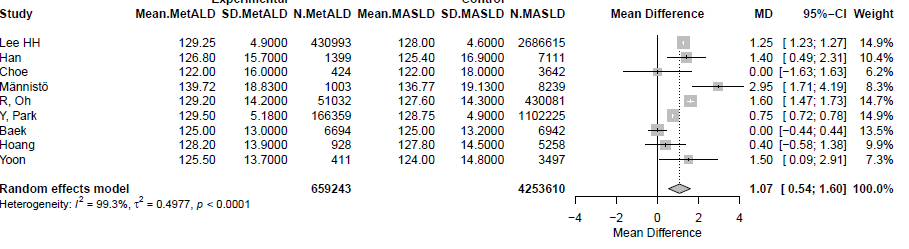


*MetALD: metabolic and alcohol related/associated liver disease; MASLD: metabolic dysfunction-associated steatotic liver disease; SLD: steatotic liver disease.*

**Supplementary Figure S52**. Forest plot of studies comparing the pooled mean diastolic blood pressure (DBP) levels of MetALD patients compared to those with MASLD in studies using serum-based non-invasive tests for diagnosis of SLD.


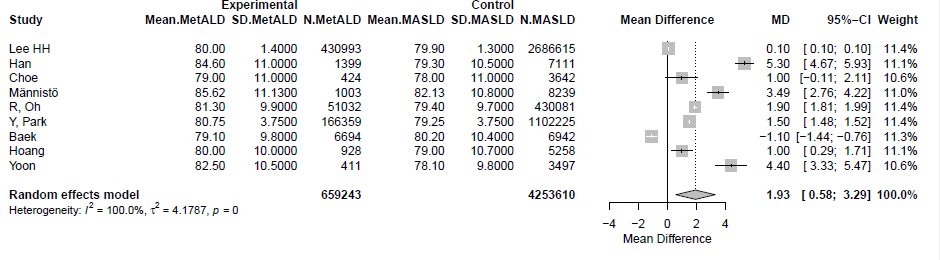


*MetALD: metabolic and alcohol related/associated liver disease; MASLD: metabolic dysfunction-associated steatotic liver disease; SLD: steatotic liver disease.*

**Supplementary Figure S53**. Forest plot of studies comparing the pooled mean high-density cholesterol (HDL-C) levels of MetALD patients compared to those with MASLD in studies using serum-based non-invasive tests for diagnosis of SLD.


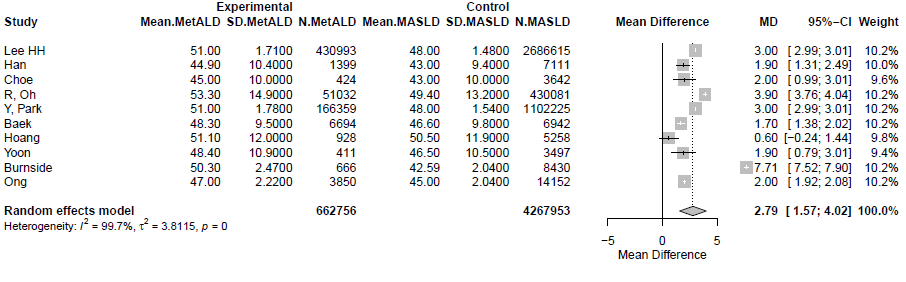


*MetALD: metabolic and alcohol related/associated liver disease; MASLD: metabolic dysfunction-associated steatotic liver disease; SLD:steatotic liver disease.*

**Supplementary Figure S54**. Forest plot of studies comparing the pooled mean low-density lipoprotein cholesterol (LDL-C) levels of MetALD patients compared to those with MASLD in studies using serum-based non-invasive tests for diagnosis of SLD.


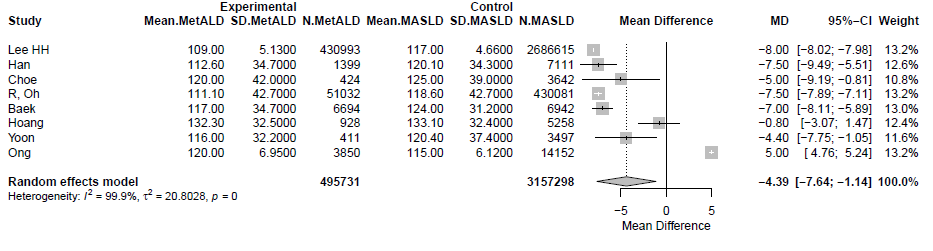


*MetALD: metabolic and alcohol related/associated liver disease; MASLD: metabolic dysfunction-associated steatotic liver disease; SLD:steatotic liver disease.*

**Supplementary Figure S55**. Forest plot of studies comparing the pooled mean triglyceride (TG) levels MetALD patients compared to those with MASLD in studies using serum-based non-invasive tests for diagnosis of SLD.

**
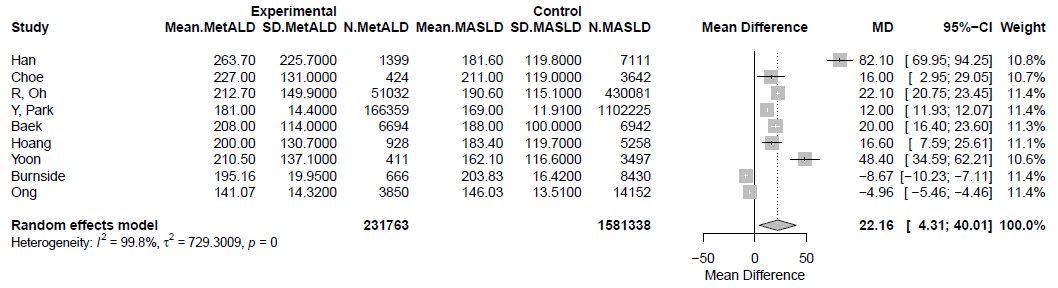
**

*MetALD: metabolic and alcohol related/associated liver disease; MASLD: metabolic dysfunction-associated steatotic liver disease; SLD:steatotic liver disease.*

**Supplementary Figure S56**. Forest plot of studies comparing the pooled mean total cholesterol levels of MetALD patients compared to those with MASLD in studies using serum-based non-invasive tests for diagnosis of SLD.


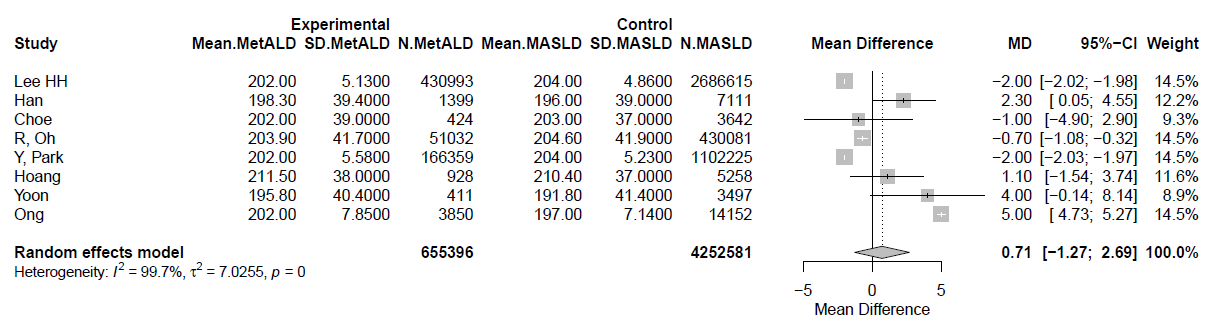


*MetALD: metabolic and alcohol related/associated liver disease; MASLD: metabolic dysfunction-associated steatotic liver disease; SLD: steatotic liver disease.*

**Supplementary Figure S57**. Forest plot of studies comparing the pooled mean fasting glucose levels of MetALD patients compared to those with MASLD using serum-based non-invasive tests for diagnosis of SLD.


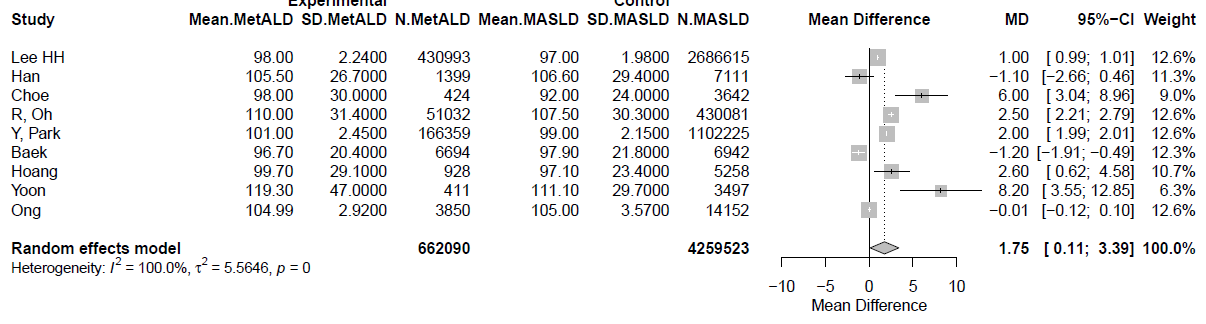


*MetALD: metabolic and alcohol related/associated liver disease; MASLD: metabolic dysfunction-associated steatotic liver disease; SLD: steatotic liver disease.*

**Supplementary Figure S58**. Forest plot of studies comparing the pooled mean glycated hemoglobin A1C (Hba1c) levels of MetALD patients compared to those with MASLD in studies using serum-based non-invasive tests for diagnosis of SLD.


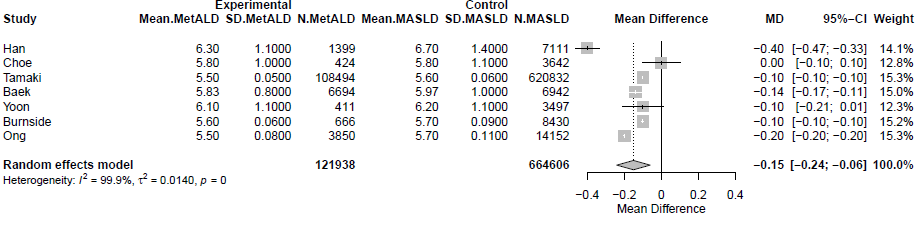


*MetALD: metabolic and alcohol related/associated liver disease; MASLD: metabolic dysfunction-associated steatotic liver disease; SLD: steatotic liver disease.*

**Supplementary Figure S59**. Forest plot of studies comparing the body mass index (BMI) of MetALD patients compared to those with MASLD in studies using serum-based non-invasive tests for diagnosis of SLD.

**
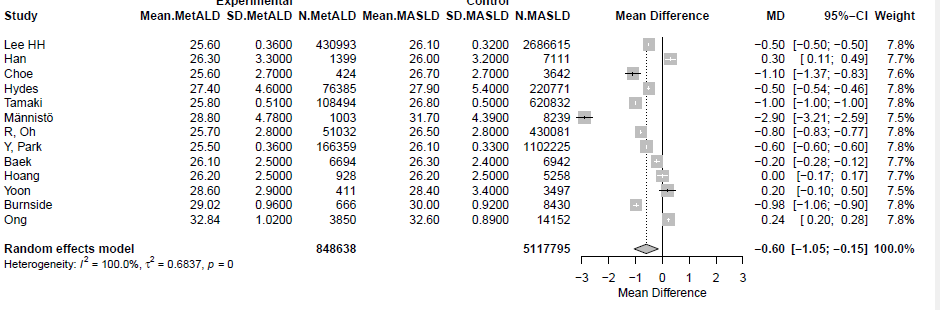
**

*MetALD: metabolic and alcohol related/associated liver disease; MASLD: metabolic dysfunction-associated steatotic liver disease; SLD: steatotic liver disease.*

**Supplementary Figure S60**. Forest plot of studies comparing the waist circumference (WC) of MetALD patients compared to those with MASLD in studies using serum-based non-invasive tests for diagnosis of SLD.


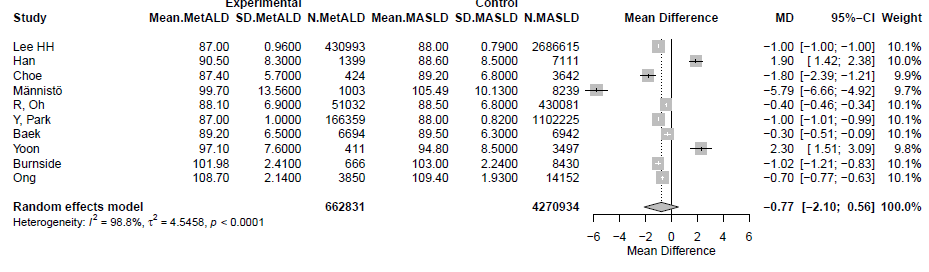


*MetALD: metabolic and alcohol related/associated liver disease; MASLD: metabolic dysfunction-associated steatotic liver disease; SLD: steatotic liver disease.*

**Supplementary Figure S61**. Forest plot of studies comparing the Homeostatic Model Assessment for Insulin Resistance (HOMA-IR) of MetALD patients compared to those with MASLD in studies using serum-based non-invasive tests for diagnosis of SLD.

**
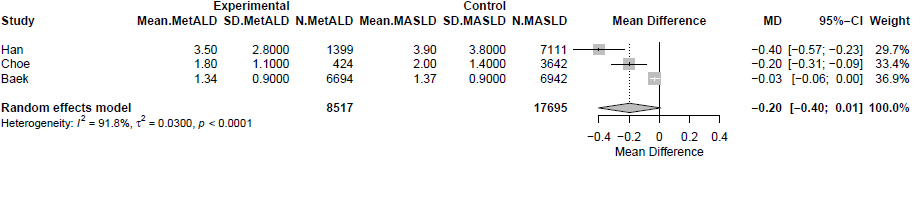
**

*MetALD: metabolic and alcohol related/associated liver disease; MASLD: metabolic dysfunction-associated steatotic liver disease; SLD: steatotic liver disease.*

**Supplementary Figure S62**. Forest plot of studies comparing the pooled mean values of estimated glomerular filtration rate (eGFR) of MetALD patients compared to those with MASLD in studies using serum-based non-invasive tests for diagnosis of SLD.


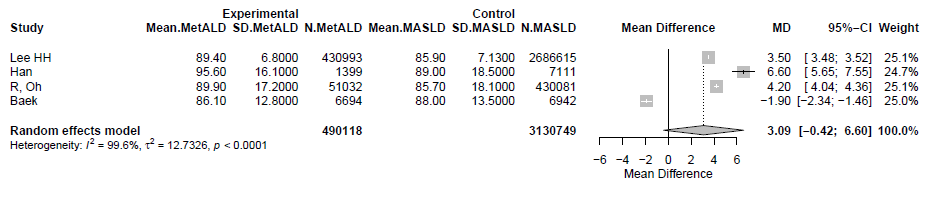


*MetALD: metabolic and alcohol related/associated liver disease; MASLD: metabolic dysfunction-associated steatotic liver disease; SLD: steatotic liver disease.*

**Supplementary Figure S63**. Forest plot of studies comparing the pooled mean values of fibrosis-4 (FIB-4) score of MetALD patients compared to those with MASLD in studies using serum-based non-invasive tests for diagnosis of SLD.

**
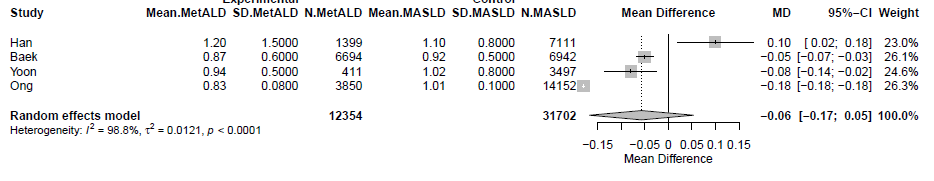
**

*MetALD: metabolic and alcohol related/associated liver disease; MASLD: metabolic dysfunction-associated steatotic liver disease; SLD: steatotic liver disease.*

**Supplementary Figure S64**. Forest plot of studies comparing the age of MetALD patients compared to those with MASLD in studies using ultrasound for diagnosis of SLD.


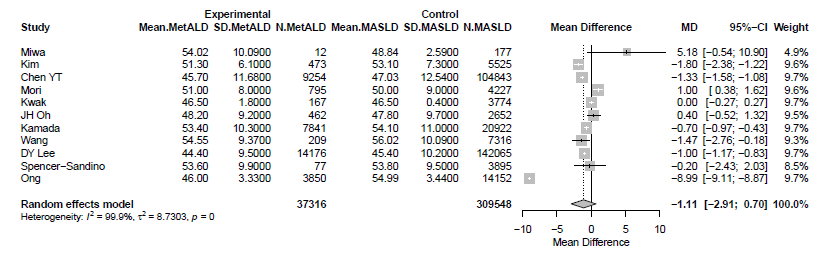


*MetALD: metabolic and alcohol related/associated liver disease; MASLD: metabolic dysfunction-associated steatotic liver disease; SLD: steatotic liver disease.*

**Supplementary Figure S65**. Forest plot of studies comparing the pooled mean aspartate aminotransferase (AST) levels of MetALD patients compared to those with MASLD in studies using ultrasound for diagnosis of SLD.


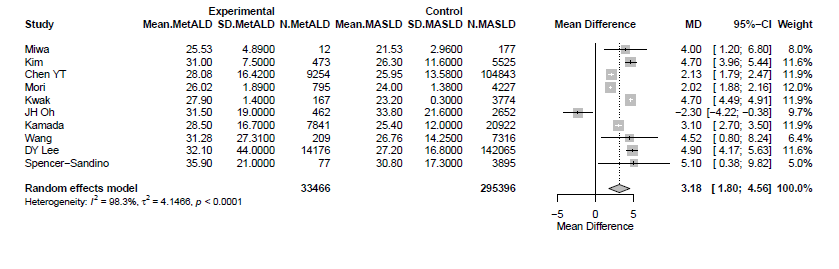


*MetALD: metabolic and alcohol related/associated liver disease; MASLD: metabolic dysfunction-associated steatotic liver disease; SLD: steatotic liver disease.*

**Supplementary Figure S66**. Forest plot of studies comparing the pooled mean alanine aminotransferase (ALT) levels of MetALD patients compared to those with MASLD in studies using serum-based non-invasive tests for diagnosis of SLD.


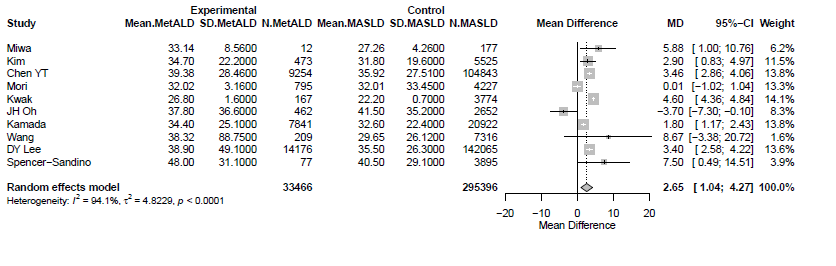


*MetALD: metabolic and alcohol related/associated liver disease; MASLD: metabolic dysfunction-associated steatotic liver disease; SLD: steatotic liver disease.*

**Supplementary Figure S67**. Forest plot of studies comparing the pooled mean Gamma-Glutamyl Transferase (GGT) levels of MetALD patients compared to those with MASLD in studies using ultrasound for diagnosis of SLD.


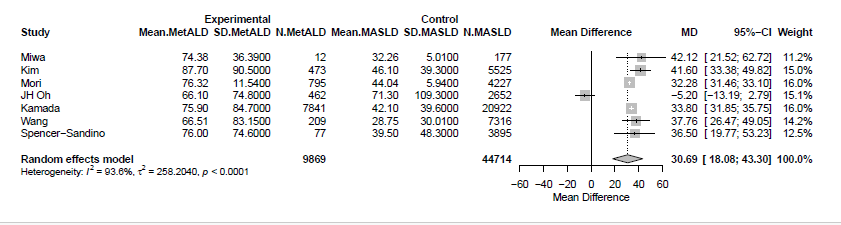


*MetALD: metabolic and alcohol related/associated liver disease; MASLD: metabolic dysfunction-associated steatotic liver disease; SLD:steatotic liver disease.*

**Supplementary Figure S68**. Forest plot of studies comparing the pooled mean platelet count (PLT) of MetALD patients compared to those with MASLD in studies using ultrasound for diagnosis of SLD.


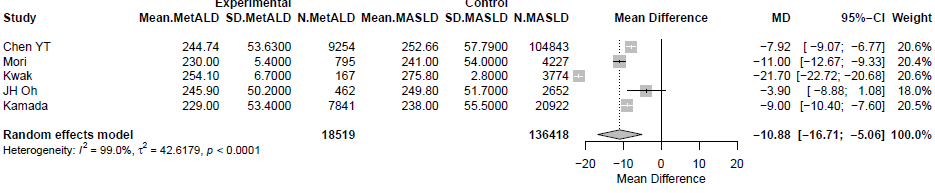


*MetALD: metabolic and alcohol related/associated liver disease; MASLD: metabolic dysfunction-associated steatotic liver disease; SLD: steatotic liver disease.*

**Supplementary Figure S69**. Forest plot of studies comparing the pooled mean systolic blood pressure (SBP) levels of MetALD patients compared to those with MASLD in studies using ultrasound for diagnosis of SLD.


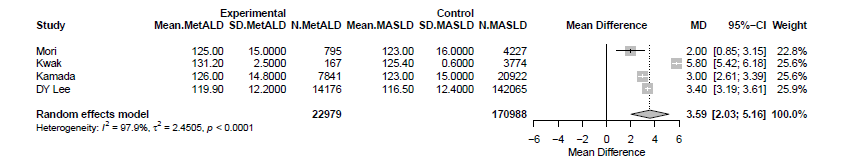


*MetALD: metabolic and alcohol related/associated liver disease; MASLD: metabolic dysfunction-associated steatotic liver disease; SLD: steatotic liver disease.*

**Supplementary Figure S70**. Forest plot of studies comparing the pooled mean diastolic blood pressure (DBP) levels of MetALD patients compared to those with MASLD in studies using ultrasound for diagnosis of SLD.


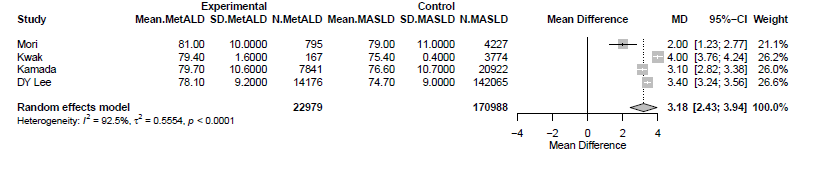


*MetALD: metabolic and alcohol related/associated liver disease; MASLD: metabolic dysfunction-associated steatotic liver disease; SLD: steatotic liver disease.*

**Supplementary Figure S71**. Forest plot of studies comparing the pooled mean high-density cholesterol (HDL-C) levels of MetALD patients compared to those with MASLD in studies using ultrasound tests for diagnosis of SLD.


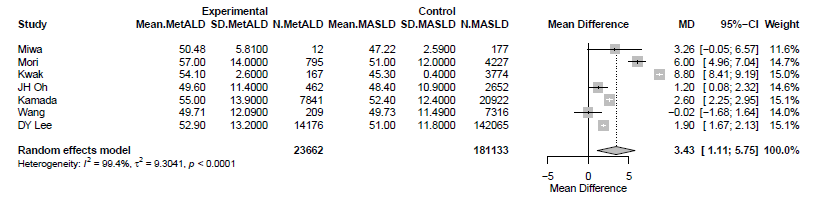


*MetALD: metabolic and alcohol related/associated liver disease; MASLD: metabolic dysfunction-associated steatotic liver disease; SLD:steatotic liver disease.*

**Supplementary Figure S72**. Forest plot of studies comparing the pooled mean low-density lipoprotein cholesterol (LDL-C) levels of MetALD patients compared to those with MASLD in studies using ultrasound tests for diagnosis of SLD.


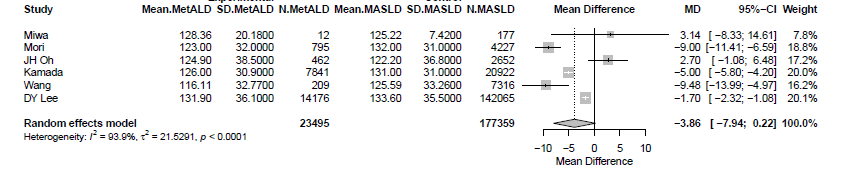


*MetALD: metabolic and alcohol related/associated liver disease; MASLD: metabolic dysfunction-associated steatotic liver disease; SLD:steatotic liver disease.*

**Supplementary Figure S73**. Forest plot of studies comparing the pooled mean triglyceride (TG) levels MetALD patients compared to those with MASLD in studies using ultrasound for diagnosis of SLD.

**
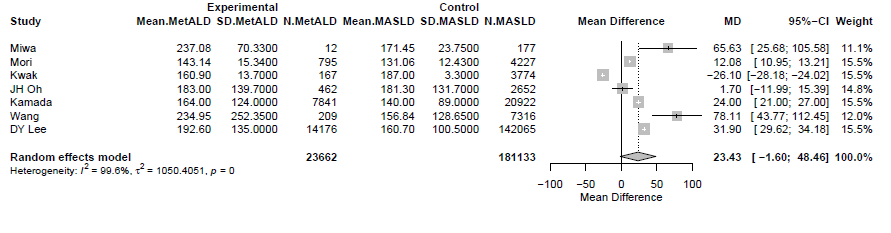
**

*MetALD: metabolic and alcohol related/associated liver disease; MASLD: metabolic dysfunction-associated steatotic liver disease; SLD:steatotic liver disease.*

**Supplementary Figure S74**. Forest plot of studies comparing the pooled mean total cholesterol levels of MetALD patients compared to those with MASLD in studies using ultrasound for diagnosis of SLD.


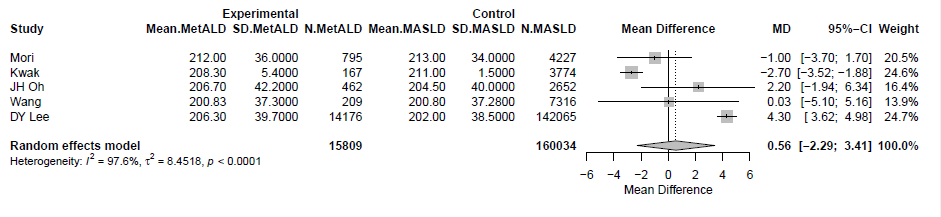


*MetALD: metabolic and alcohol related/associated liver disease; MASLD: metabolic dysfunction-associated steatotic liver disease; SLD: steatotic liver disease.*

**Supplementary Figure S75**. Forest plot of studies comparing the pooled mean fasting glucose levels of MetALD patients compared to those with MASLD using ultrasound for diagnosis of SLD.


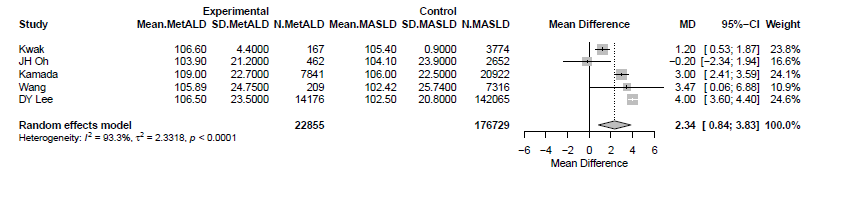


*MetALD: metabolic and alcohol related/associated liver disease; MASLD: metabolic dysfunction-associated steatotic liver disease; SLD: steatotic liver disease.*

**Supplementary Figure S76**. Forest plot of studies comparing the pooled mean glycated hemoglobin A1C (Hba1c) levels of MetALD patients compared to those with MASLD in studies using serum-based non-invasive tests for diagnosis of SLD.


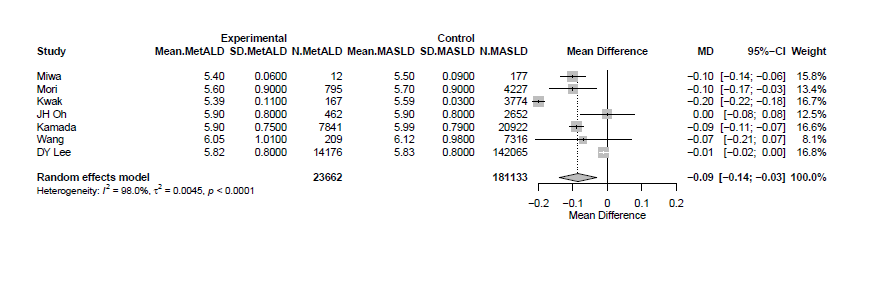


*MetALD: metabolic and alcohol related/associated liver disease; MASLD: metabolic dysfunction-associated steatotic liver disease; SLD: steatotic liver disease.*

**Supplementary Figure S77**. Forest plot of studies comparing the body mass index (BMI) of MetALD patients compared to those with MASLD in studies using ultrasound for diagnosis of SLD.

**
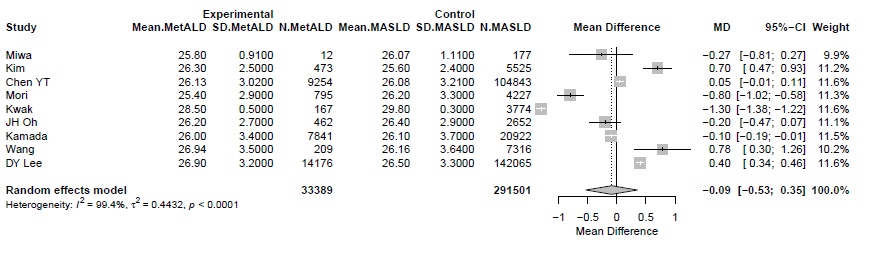
**

*MetALD: metabolic and alcohol related/associated liver disease; MASLD: metabolic dysfunction-associated steatotic liver disease; SLD: steatotic liver disease.*

**Supplementary Figure S78**. Forest plot of studies comparing the waist circumference (WC) of MetALD patients compared to those with MASLD in studies using ultrasound for diagnosis of SLD.


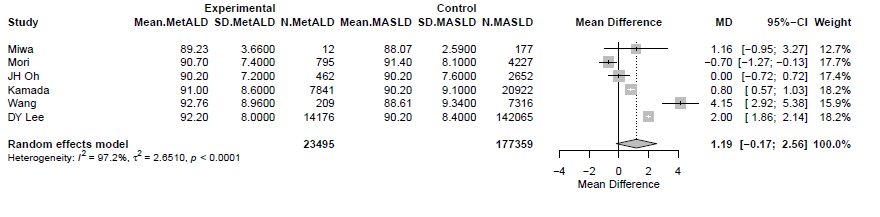


*MetALD: metabolic and alcohol related/associated liver disease; MASLD: metabolic dysfunction-associated steatotic liver disease; SLD: steatotic liver disease.*

**Supplementary Figure S79**. Forest plot of studies comparing the Homeostatic Model Assessment for Insulin Resistance (HOMA-IR) of MetALD patients compared to those with MASLD in studies using ultrasound for diagnosis of SLD.

**
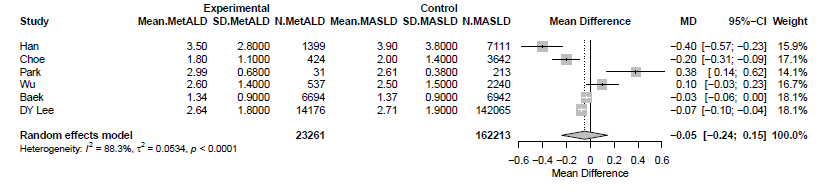
**

*MetALD: metabolic and alcohol related/associated liver disease; MASLD: metabolic dysfunction-associated steatotic liver disease; SLD: steatotic liver disease.*

**Supplementary Figure S80**. Forest plot of studies comparing the pooled mean values of estimated glomerular filtration rate (eGFR) of MetALD patients compared to those with MASLD in studies using ultrasound for diagnosis of SLD.


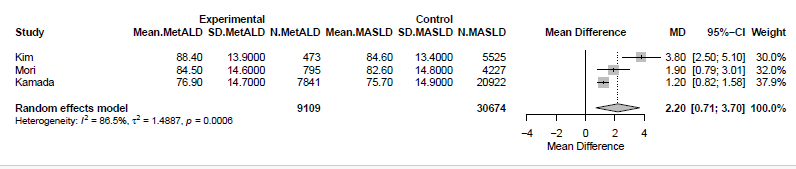


*MetALD: metabolic and alcohol related/associated liver disease; MASLD: metabolic dysfunction-associated steatotic liver disease; SLD: steatotic liver disease.*

**Supplementary Figure S81**. Forest plot of studies comparing the pooled mean values of fibrosis-4 (FIB-4) score of MetALD patients compared to those with MASLD in studies using ultrasound for diagnosis of SLD.

**
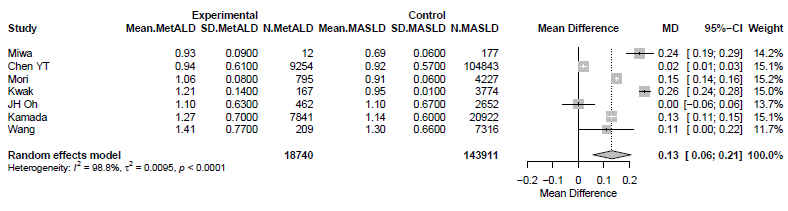
**

*MetALD: metabolic and alcohol related/associated liver disease; MASLD: metabolic dysfunction-associated steatotic liver disease; SLD: steatotic liver disease.*

**Supplementary Figure S82**. Forest plot of studies comparing the pooled mean values of liver stiffness measurements (LSM) of MetALD patients compared to those with MASLD in studies using ultrasound for diagnosis of SLD.

**
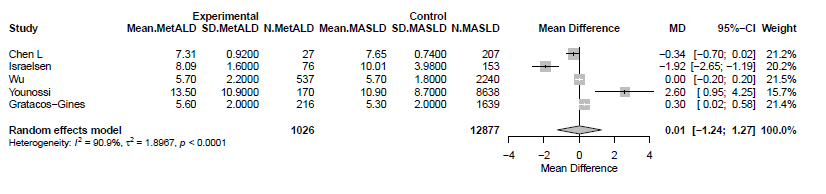
**

*MetALD: metabolic and alcohol related/associated liver disease; MASLD: metabolic dysfunction-associated steatotic liver disease; SLD: steatotic liver disease.*

**Supplementary Figure S83**. Forest plot of studies comparing the pooled mean values of Nonalcoholic Fatty Liver Disease Fibrosis Score (NFS) of MetALD patients compared to those with MASLD in studies using ultrasound for diagnosis of SLD.

*
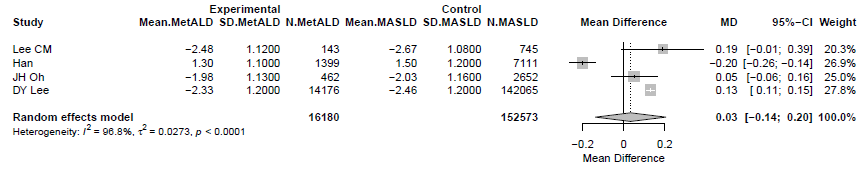
*

*MetALD: metabolic and alcohol related/associated liver disease; MASLD: metabolic dysfunction-associated steatotic liver disease; SLD: steatotic liver disease.*

**Supplementary Figure S84**. Forest plot of studies comparing the pooled mean values of Magnetic Resonance elastography (MRE) of MetALD patients compared to those with MASLD in studies using ultrasound for diagnosis of SLD.

*
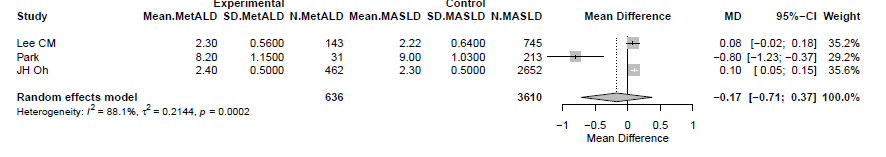
*

*MetALD: metabolic and alcohol related/associated liver disease; MASLD: metabolic dysfunction-associated steatotic liver disease; SLD: steatotic liver disease.*

**Supplementary Figure S85**. Forest plot of studies comparing the age of Αsian MetALD patients compared to those with ALD.


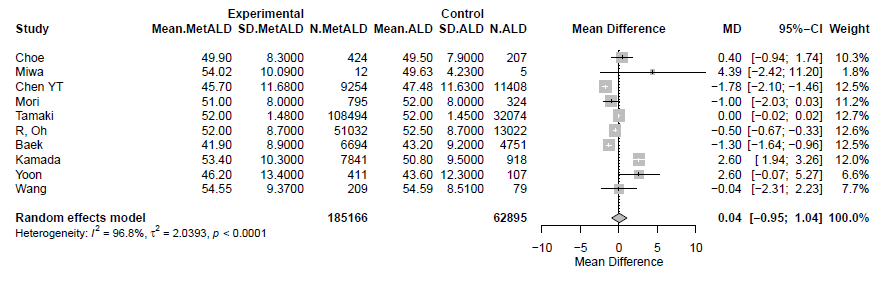


*MetALD: metabolic and alcohol related/associated liver disease; ALD: alcohol-related liver disease; SLD: steatotic liver disease.*

**Supplementary Figure S86**. Forest plot of studies comparing the pooled mean aspartate aminotransferase (AST) levels of Αsian MetALD patients compared to those with ALD.


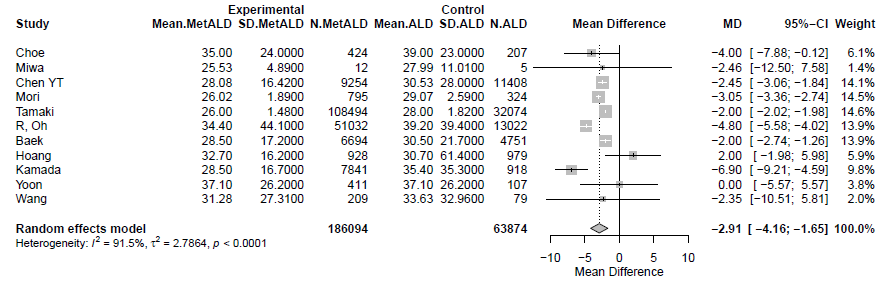


*MetALD: metabolic and alcohol related/associated liver disease; ALD: alcohol-related liver disease.*

**Supplementary Figure S87**. Forest plot of studies comparing the pooled mean alanine aminotransferase (ALT) levels of Αsian MetALD patients compared to those with ALD.


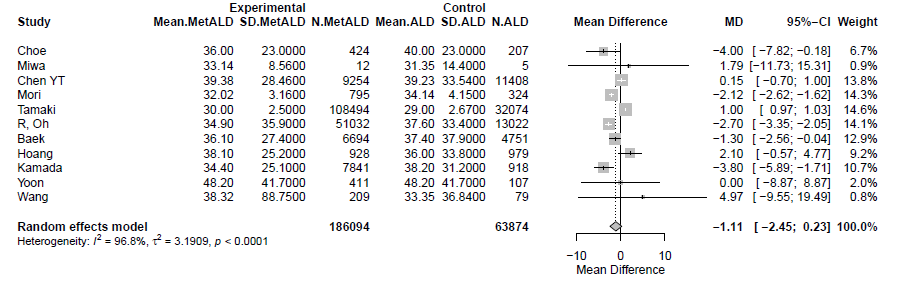


*MetALD: metabolic and alcohol related/associated liver disease; ALD: alcohol-related liver disease.*

**Supplementary Figure S88**. Forest plot of studies comparing the pooled mean Gamma-Glutamyl Transferase (GGT) levels of Αsian MetALD patients compared to those with ALD.


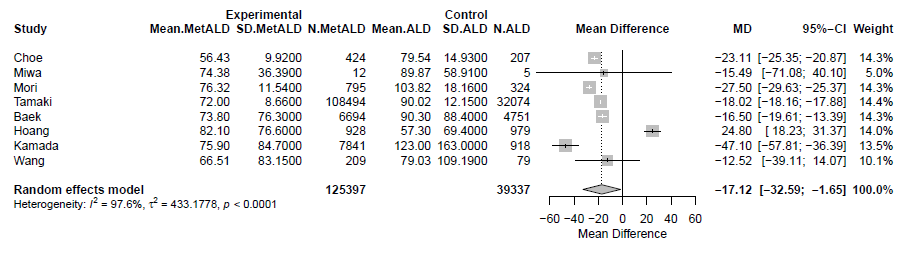


*MetALD: metabolic and alcohol related/associated liver disease; ALD: alcohol-related liver disease.*

**Supplementary Figure S89**. Forest plot of studies comparing the pooled mean platelet count (PLT) of MetALD patients compared to those with ALD.


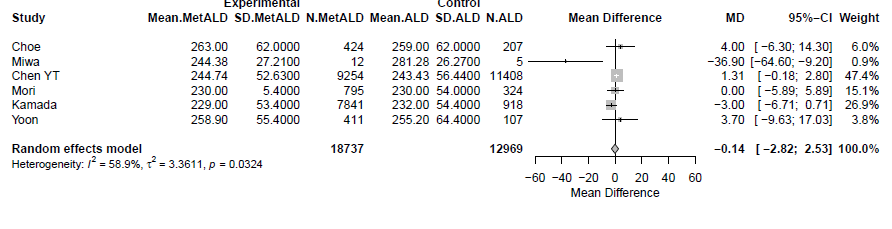


*MetALD: metabolic and alcohol related/associated liver disease; ALD: alcohol-related liver disease.*

**Supplementary Figure S90**. Forest plot of studies comparing the estimated glomerular filtration rate (eGFR) of Αsian MetALD patients compared to those with ALD.


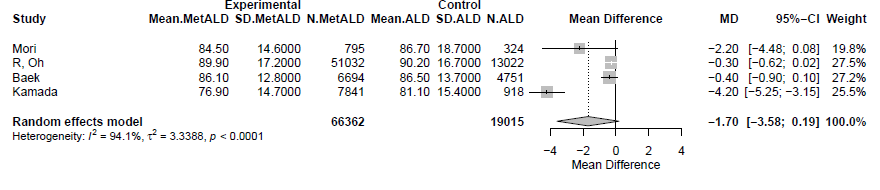


*MetALD: metabolic and alcohol related/associated liver disease; ALD: alcohol-related liver disease.*

**Supplementary Figure S91**. Forest plot of studies comparing the pooled mean systolic blood pressure (SBP) levels of Αsian MetALD patients compared to those with ALD.


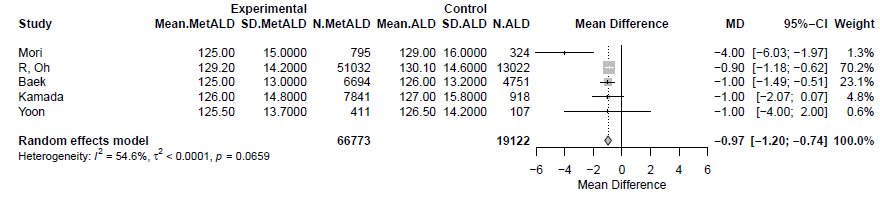


*MetALD: metabolic and alcohol related/associated liver disease; ALD: alcohol-related liver disease.*

**Supplementary Figure S92**. Forest plot of studies comparing the pooled mean diastolic blood pressure (DBP) levels of Αsian MetALD patients compared to those with ALD.


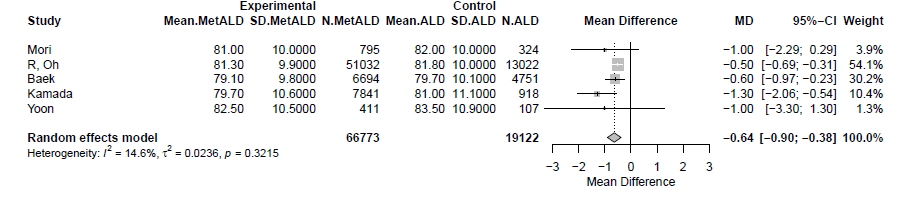


*MetALD: metabolic and alcohol related/associated liver disease; ALD: alcohol-related liver disease.*

**Supplementary Figure S93**. Forest plot of studies comparing the pooled mean high-density cholesterol (HDL-C) levels of Αsian MetALD patients compared to those with ALD.


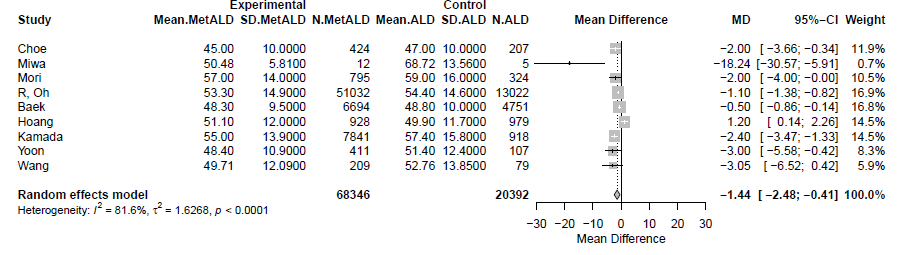


*MetALD: metabolic and alcohol related/associated liver disease; ALD: alcohol-related liver disease.*

**Supplementary Figure S94**. Forest plot of studies comparing the pooled mean low-density lipoprotein cholesterol (LDL-C) levels of Αsian MetALD patients compared to those with ALD.


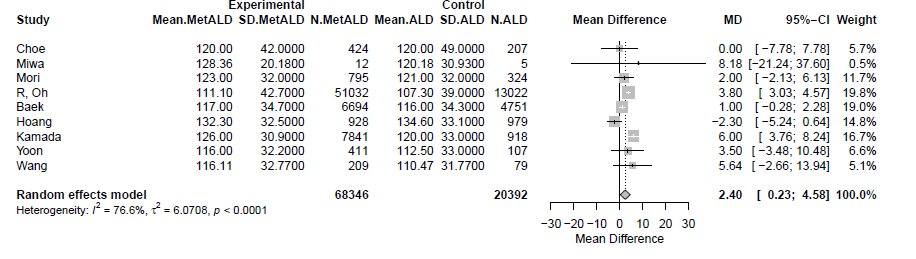


*MetALD: metabolic and alcohol related/associated liver disease; ALD: alcohol-related liver disease.*

**Supplementary Figure S95**. Forest plot of studies comparing the pooled mean triglyceride (TG) levels of Αsian MetALD patients compared to those with ALD.

**
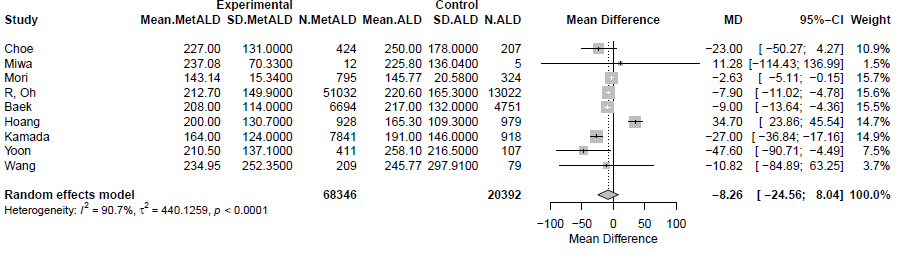
**

*MetALD: metabolic and alcohol related/associated liver disease; ALD: alcohol-related liver disease.*

**Supplementary Figure S96**. Forest plot of studies comparing the pooled mean total cholesterol levels of Αsian MetALD patients compared to those with ALD.

**
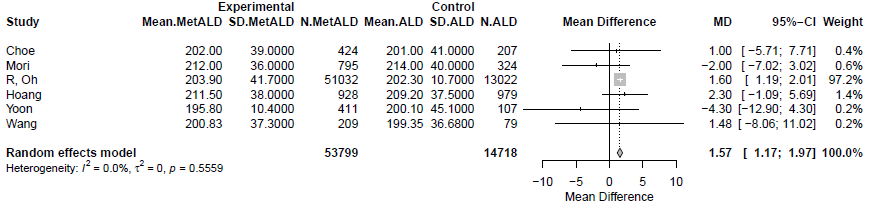
**

*MetALD: metabolic and alcohol related/associated liver disease; ALD: alcohol-related liver disease.*

**Supplementary Figure S97**. Forest plot of studies comparing the pooled mean fasting glucose levels of Αsian MetALD patients compared to those with ALD.


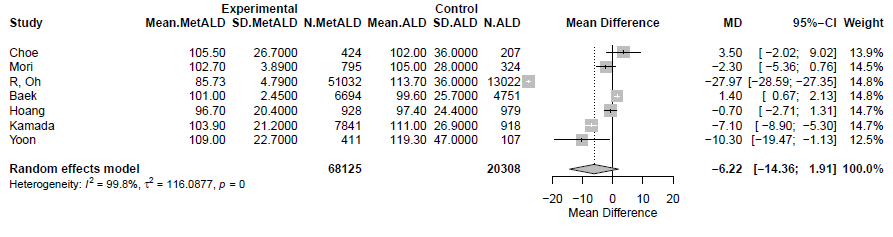


*MetALD: metabolic and alcohol related/associated liver disease; ALD: alcohol-related liver disease.*

**Supplementary Figure S98**. Forest plot of studies comparing the pooled mean glycated hemoglobin A1C (Hba1c) levels of Αsian MetALD patients compared to those with ALD.


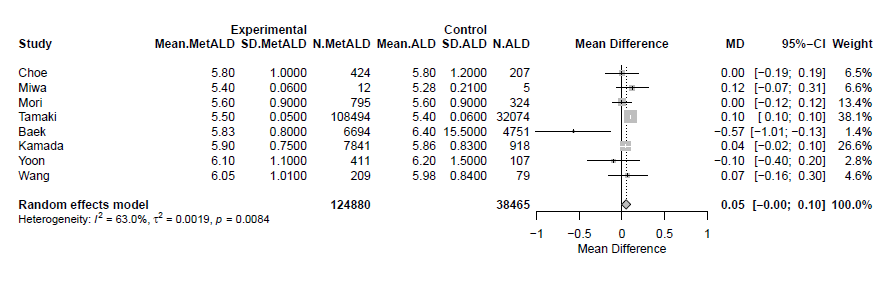


*MetALD: metabolic and alcohol related/associated liver disease; ALD: alcohol-related liver disease.*

**Supplementary Figure S99**. Forest plot of studies comparing the body mass index (BMI) of Αsian MetALD patients compared to those with ALD.

**
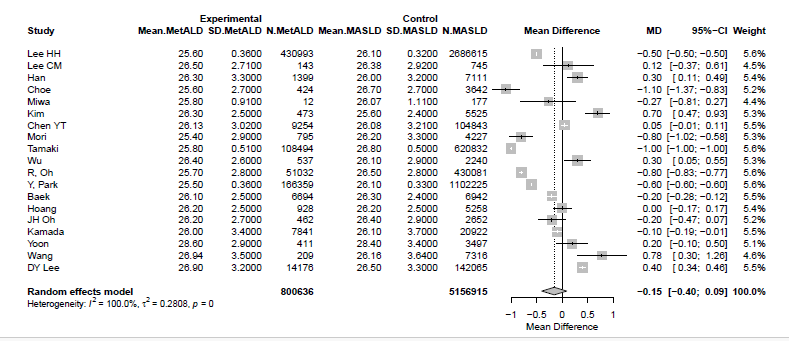
**

*MetALD: metabolic and alcohol related/associated liver disease; ALD: alcohol-related liver disease.*

**Supplementary Figure S100**. Forest plot of studies comparing the waist circumference (WC) of Αsian MetALD patients compared to those with ALD.


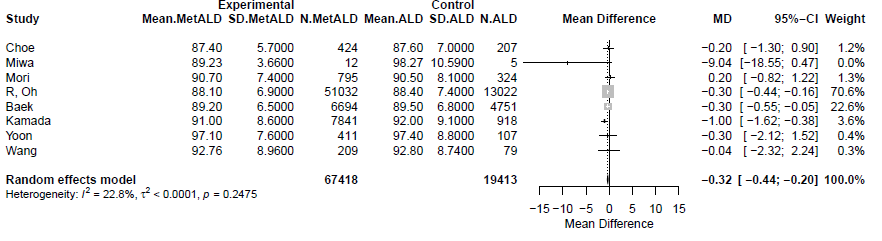


*MetALD: metabolic and alcohol related/associated liver disease; ALD: alcohol-related liver disease.*

**Supplementary Figure S101**. Forest plot of studies comparing the age of non Αsian MetALD patients compared to those with ALD.


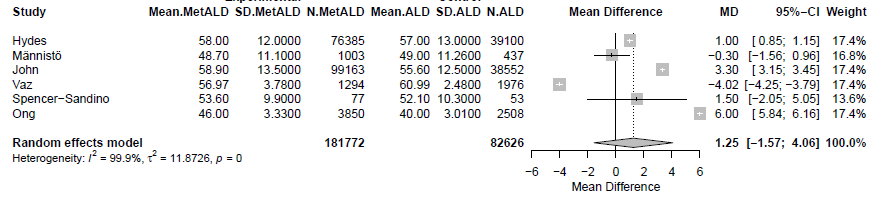


*MetALD: metabolic and alcohol related/associated liver disease; ALD: alcohol-related liver disease.*

**Supplementary Figure S102**. Forest plot of studies comparing the pooled mean aspartate aminotransferase (AST) levels of non Αsian MetALD patients compared to those with ALD.


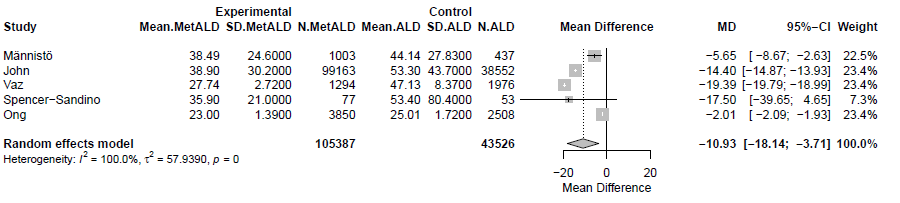


*MetALD: metabolic and alcohol related/associated liver disease; ALD: alcohol-related liver disease.*

**Supplementary Figure S103.** Forest plot of studies comparing the pooled mean alanine aminotransferase (ALT) levels of non Αsian MetALD patients compared to those with ALD.

*MetALD: metabolic and alcohol related/associated liver disease; ALD: alcohol-related liver disease.*

**Supplementary Figure S104**. Forest plot of studies comparing the pooled mean Gamma-Glutamyl Transferase (GGT) levels of non Αsian MetALD patients compared to those with ALD.

*MetALD: metabolic and alcohol related/associated liver disease; ALD: alcohol-related liver disease.*

**Supplementary Figure S105**. Forest plot of studies comparing the age of MetALD patients compared to those with ALD in studies using serum-based non-invasive tests for the diagnosis of SLD.

*MetALD: metabolic and alcohol related/associated liver disease; ALD: alcohol-related liver disease; SLD: steatotic liver disease.*

**Supplementary Figure S106**. Forest plot of studies comparing the pooled mean aspartate aminotransferase (AST) levels of MetALD patients compared to those with ALD in studies using serum-based non-invasive tests for the diagnosis of SLD.

*MetALD: metabolic and alcohol related/associated liver disease; ALD: alcohol-related liver disease; SLD: steatotic liver disease.*

**Supplementary Figure S107**. Forest plot of studies comparing the pooled mean alanine aminotransferase (ALT) levels of MetALD patients compared to those with ALD in studies using serum-based non-invasive tests for the diagnosis of SLD.

*MetALD: metabolic and alcohol related/associated liver disease; ALD: alcohol-related liver disease; SLD: steatotic liver disease; SLD: steatotic liver disease.*

*.*

**Supplementary Figure S108**. Forest plot of studies comparing the pooled mean Gamma-Glutamyl Transferase (GGT) levels of MetALD patients compared to those with ALD in studies using serum-based non-invasive tests for the diagnosis of SLD.

*MetALD: metabolic and alcohol related/associated liver disease; ALD: alcohol-related liver disease; SLD: steatotic liver disease; SLD: steatotic liver disease.*

**Supplementary Figure S109**. Forest plot of studies comparing the pooled mean platelet count (PLT) of MetALD patients compared to those with ALD in studies using serum-based non-invasive tests for the diagnosis of SLD.

*MetALD: metabolic and alcohol related/associated liver disease; ALD: alcohol-related liver disease; SLD: steatotic liver disease; SLD: steatotic liver disease.*

**Supplementary Figure S110**. Forest plot of studies comparing the pooled mean systolic blood pressure (SBP) levels of MetALD patients compared to those with ALD in studies using serum-based non-invasive tests for the diagnosis of SLD.

*MetALD: metabolic and alcohol related/associated liver disease; ALD: alcohol-related liver disease; SLD: steatotic liver disease; SLD: steatotic liver disease.*

**Supplementary Figure S111**. Forest plot of studies comparing the pooled mean diastolic blood pressure (DBP) levels of MetALD patients compared to those with ALD in studies using serum-based non-invasive tests for the diagnosis of SLD.

*MetALD: metabolic and alcohol related/associated liver disease; ALD: alcohol-related liver disease; SLD: steatotic liver disease; SLD: steatotic liver disease.*

**Supplementary Figure S112**. Forest plot of studies comparing the pooled mean high-density cholesterol (HDL-C) levels of MetALD patients compared to those with ALD in studies using serum-based non-invasive tests for the diagnosis of SLD.

*MetALD: metabolic and alcohol related/associated liver disease; ALD: alcohol-related liver disease; SLD: steatotic liver disease; SLD: steatotic liver disease.*

**Supplementary Figure S113**. Forest plot of studies comparing the pooled mean low-density lipoprotein cholesterol (LDL-C) levels of MetALD patients compared to those with ALD in studies using serum-based non-invasive tests for the diagnosis of SLD.

*MetALD: metabolic and alcohol related/associated liver disease; ALD: alcohol-related liver disease; SLD: steatotic liver disease; SLD: steatotic liver disease.*

**Supplementary Figure S114**. Forest plot of studies comparing the pooled mean triglyceride (TG) levels of MetALD patients compared to those with ALD in studies using serum-based non-invasive tests for the diagnosis of SLD.

*MetALD: metabolic and alcohol related/associated liver disease; ALD: alcohol-related liver disease; SLD: steatotic liver disease; SLD: steatotic liver disease.*

**Supplementary Figure S115**. Forest plot of studies comparing the pooled mean total cholesterol levels of MetALD patients compared to those with ALD in studies using serum-based non-invasive tests for the diagnosis of SLD.

*MetALD: metabolic and alcohol related/associated liver disease; ALD: alcohol-related liver disease; SLD: steatotic liver disease; SLD: steatotic liver disease.*

**Supplementary Figure S116**. Forest plot of studies comparing the pooled mean fasting glucose levels of MetALD patients compared to those with ALD in studies using serum-based non-invasive tests for the diagnosis of SLD.

*MetALD: metabolic and alcohol related/associated liver disease; ALD: alcohol-related liver disease; SLD: steatotic liver disease; SLD: steatotic liver disease.*

**Supplementary Figure S117**. Forest plot of studies comparing the pooled mean glycated hemoglobin A1C (Hba1c) levels of MetALD patients compared to those with ALD in studies using serum-based non-invasive tests for the diagnosis of SLD.

*MetALD: metabolic and alcohol related/associated liver disease; ALD: alcohol-related liver disease; SLD: steatotic liver disease; SLD: steatotic liver disease*

**Supplementary Figure S118**. Forest plot of studies comparing the body mass index (BMI) of MetALD patients compared to those with ALD in studies using serum-based non-invasive tests for the diagnosis of SLD.

*MetALD: metabolic and alcohol related/associated liver disease; ALD: alcohol-related liver disease; SLD: steatotic liver disease; SLD: steatotic liver disease.*

**Supplementary Figure S119**. Forest plot of studies comparing the waist circumference (WC) of MetALD patients compared to those with ALD in studies using serum-based non-invasive tests for the diagnosis of SLD.

*MetALD: metabolic and alcohol related/associated liver disease; ALD: alcohol-related liver disease; SLD: steatotic liver disease; SLD: steatotic liver disease.*

**Supplementary Figure S120**. Forest plot of studies comparing the age of MetALD patients compared to those with ALD in studies using ultrasound for the diagnosis of SLD.

*MetALD: metabolic and alcohol related/associated liver disease; ALD: alcohol-related liver disease; SLD: steatotic liver disease.*

**Supplementary Figure S121**. Forest plot of studies comparing the pooled mean aspartate aminotransferase (AST) levels of MetALD patients compared to those with ALD in studies using ultrasound for the diagnosis of SLD.

*MetALD: metabolic and alcohol related/associated liver disease; ALD: alcohol-related liver disease; SLD: steatotic liver disease.*

**Supplementary Figure S122**. Forest plot of studies comparing the pooled mean alanine aminotransferase (ALT) levels of MetALD patients compared to those with ALD in studies using ultrasound for the diagnosis of SLD.

*MetALD: metabolic and alcohol related/associated liver disease; ALD: alcohol-related liver disease; SLD: steatotic liver disease; SLD: steatotic liver disease.*

*.*

**Supplementary Figure S123**. Forest plot of studies comparing the pooled mean Gamma-Glutamyl Transferase (GGT) levels of MetALD patients compared to those with ALD in studies using ultrasound for the diagnosis of SLD.

*MetALD: metabolic and alcohol related/associated liver disease; ALD: alcohol-related liver disease; SLD: steatotic liver disease; SLD: steatotic liver disease.*

**Supplementary Figure S124**. Forest plot of studies comparing the pooled mean platelet count (PLT) of MetALD patients compared to those with ALD in studies using ultrasound for the diagnosis of SLD.

*MetALD: metabolic and alcohol related/associated liver disease; ALD: alcohol-related liver disease; SLD: steatotic liver disease; SLD: steatotic liver disease.*

**Supplementary Figure S125**. Forest plot of studies comparing the pooled mean high-density cholesterol (HDL-C) levels of MetALD patients compared to those with ALD in studies using ultrasound for the diagnosis of SLD.

*MetALD: metabolic and alcohol related/associated liver disease; ALD: alcohol-related liver disease; SLD: steatotic liver disease; SLD: steatotic liver disease.*

**Supplementary Figure S126**. Forest plot of studies comparing the pooled mean low-density lipoprotein cholesterol (LDL-C) levels of MetALD patients compared to those with ALD in studies using serum-based non-invasive tests for the diagnosis of SLD.

*MetALD: metabolic and alcohol related/associated liver disease; ALD: alcohol-related liver disease; SLD: steatotic liver disease; SLD: steatotic liver disease.*

**Supplementary Figure S127**. Forest plot of studies comparing the pooled mean triglyceride (TG) levels of MetALD patients compared to those with ALD in studies using ultrasound for the diagnosis of SLD.

*MetALD: metabolic and alcohol related/associated liver disease; ALD: alcohol-related liver disease; SLD: steatotic liver disease; SLD: steatotic liver disease.*

**Supplementary Figure S128**. Forest plot of studies comparing the pooled mean glycated hemoglobin A1C (Hba1c) levels of MetALD patients compared to those with ALD in studies using ultrasound for the diagnosis of SLD.

*MetALD: metabolic and alcohol related/associated liver disease; ALD: alcohol-related liver disease; SLD: steatotic liver disease; SLD: steatotic liver disease*

**Supplementary Figure S129**. Forest plot of studies comparing the body mass index (BMI) of MetALD patients compared to those with ALD in studies using ultrasound for the diagnosis of SLD.

*MetALD: metabolic and alcohol related/associated liver disease; ALD: alcohol-related liver disease; SLD: steatotic liver disease; SLD: steatotic liver disease.*

**Supplementary Figure S130**. Forest plot of studies comparing the waist circumference (WC) of MetALD patients compared to those with ALD in studies using ultrasound for the diagnosis of SLD.

*MetALD: metabolic and alcohol related/associated liver disease; ALD: alcohol-related liver disease; SLD: steatotic liver disease; SLD: steatotic liver disease.*

**Supplementary Figure S131**. Sensitivity analysis excluding low-quality studies (NOS ≤5) and conference abstracts comparing the age between patients with MetALD, compared to those with MASLD.

*MetALD: metabolic and alcohol related/associated liver disease; MASLD: metabolic dysfunction-associated steatotic liver disease.*

**Supplementary Figure S132**. Sensitivity analysis excluding low-quality studies (NOS ≤5) and conference abstracts comparing the pooled mean levels of aspartate aminotransferase (AST) between patients with MetALD, compared to those with MASLD.

*MetALD: metabolic and alcohol related/associated liver disease; MASLD: metabolic dysfunction-associated steatotic liver disease.*

**Supplementary Figure S133**. Sensitivity analysis excluding low-quality studies (NOS ≤5) and conference abstracts comparing the pooled mean levels of alanine aminotransferase (ALT) between patients with MetALD, compared to those with MASLD.

*MetALD: metabolic and alcohol related/associated liver disease; MASLD: metabolic dysfunction-associated steatotic liver disease.*

**Supplementary Figure S134**. Sensitivity analysis excluding low-quality studies (NOS ≤5) and conference abstracts comparing the pooled mean levels of GGT between patients with MetALD, compared to those with MASLD.

*MetALD: metabolic and alcohol related/associated liver disease; MASLD: metabolic dysfunction-associated steatotic liver disease.*

**Supplementary Figure S135**. Sensitivity analysis excluding low-quality studies (NOS ≤5) and conference abstracts comparing the pooled mean levels of platelet count (PLT) between patients with MetALD, compared to those with MASLD.

*MetALD: metabolic and alcohol related/associated liver disease; MASLD: metabolic dysfunction-associated steatotic liver disease.*

**Supplementary Figure S136**. Sensitivity analysis excluding low-quality studies (NOS ≤5) and conference abstracts comparing the pooled mean levels of estimated glomerular filtration rate (Egfr) between patients with MetALD, compared to those with MASLD.

*MetALD: metabolic and alcohol related/associated liver disease; MASLD: metabolic dysfunction-associated steatotic liver disease.*

**Supplementary Figure S137**. Sensitivity analysis excluding low-quality studies (NOS ≤5) and conference abstracts comparing the pooled mean levels of systolic blood pressure (SBP) between patients with MetALD, compared to those with MASLD.

*MetALD: metabolic and alcohol related/associated liver disease; MASLD: metabolic dysfunction-associated steatotic liver disease.*

**Supplementary Figure S138**. Sensitivity analysis excluding low-quality studies (NOS ≤5) and conference abstracts comparing the pooled mean levels of diastolic blood pressure (DBP) between patients with MetALD, compared to those with MASLD.

*MetALD: metabolic and alcohol related/associated liver disease; MASLD: metabolic dysfunction-associated steatotic liver disease.*

**Supplementary Figure S139**. Sensitivity analysis excluding low-quality studies (NOS ≤5) and conference abstracts comparing the pooled mean levels of high-density lipoprotein (HDL-C) between patients with MetALD, compared to those with MASLD.

*MetALD: metabolic and alcohol related/associated liver disease; MASLD: metabolic dysfunction-associated steatotic liver disease.*

**Supplementary Figure S140**. Sensitivity analysis excluding low-quality studies (NOS ≤5) and conference abstracts comparing the pooled mean levels of low-density lipoprotein cholesterol (LDL-C) between patients with MetALD, compared to those with MASLD.

*MetALD: metabolic and alcohol related/associated liver disease; MASLD: metabolic dysfunction-associated steatotic liver disease.*

**Supplementary Figure S141**. Sensitivity analysis excluding low-quality studies (NOS ≤5) and conference abstracts comparing the pooled mean levels of triglycerides (TG) between patients with MetALD, compared to those with MASLD.

*MetALD: metabolic and alcohol related/associated liver disease; MASLD: metabolic dysfunction-associated steatotic liver disease.*

**Supplementary Figure S142**. Sensitivity analysis excluding low-quality studies (NOS ≤5) and conference abstracts comparing the pooled mean levels of fasting glucose between patients with MetALD, compared to those with MASLD.

*MetALD: metabolic and alcohol related/associated liver disease; MASLD: metabolic dysfunction-associated steatotic liver disease.*

**Supplementary Figure S143**. Sensitivity analysis excluding low-quality studies (NOS ≤5) and conference abstracts comparing the pooled mean levels of HbA1c between patients with MetALD, compared to those with MASLD.

*MetALD: metabolic and alcohol related/associated liver disease; MASLD: metabolic dysfunction-associated steatotic liver disease.*

**Supplementary Figure S144**. Sensitivity analysis excluding low-quality studies (NOS ≤5) and conference abstracts comparing the pooled mean levels of body mass index (BMI) between patients with MetALD, compared to those with MASLD.

*MetALD: metabolic and alcohol related/associated liver disease; MASLD: metabolic dysfunction-associated steatotic liver disease.*

**Supplementary Figure S145**. Sensitivity analysis excluding low-quality studies (NOS ≤5) and conference abstracts comparing the pooled mean levels of Homeostasis Model Assessment of Insulin Resistance (HOMA-IR) between patients with MetALD, compared to those with MASLD.

*MetALD: metabolic and alcohol related/associated liver disease; MASLD: metabolic dysfunction-associated steatotic liver disease.*

**Supplementary Figure S146**. Sensitivity analysis excluding low-quality studies (NOS ≤5) and conference abstracts comparing the pooled mean levels of waist circumference (WC) between patients with MetALD, compared to those with MASLD.

*MetALD: metabolic and alcohol related/associated liver disease; MASLD: metabolic dysfunction-associated steatotic liver disease.*

**Supplementary Figure S147**. Sensitivity analysis excluding low-quality studies (NOS ≤5) and conference abstracts comparing the pooled mean levels of fibrosis-4 (FIB-4) score between patients with MetALD, compared to those with MASLD.

*MetALD: metabolic and alcohol related/associated liver disease; MASLD: metabolic dysfunction-associated steatotic liver disease.*

**Supplementary Figure S148**. Sensitivity analysis excluding low-quality studies (NOS ≤5) and conference abstracts comparing the pooled mean levels of fatty liver index (FLI) between patients with MetALD, compared to those with MASLD.

*MetALD: metabolic and alcohol related/associated liver disease; MASLD: metabolic dysfunction-associated steatotic liver disease.*

**Supplementary Figure S149**. Sensitivity analysis excluding low-quality studies (NOS ≤5) and conference abstracts comparing the age between patients with MetALD, compared to those with ALD.

*MetALD: metabolic and alcohol related/associated liver disease; ALD: alcohol-related liver disease.*

**Supplementary Figure S150**. Sensitivity analysis excluding low-quality studies (NOS ≤5) and conference abstracts comparing the pooled mean levels of aspartate aminotransferase (AST) between patients with MetALD, compared to those ALD.

*MetALD: metabolic and alcohol related/associated liver disease; ALD: alcohol-related liver disease.*

**Supplementary Figure S151**. Sensitivity analysis excluding low-quality studies (NOS ≤5) and conference abstracts comparing the pooled mean levels of alanine aminotransferase (ALT) between patients with MetALD, compared to those with ALD.

*MetALD: metabolic and alcohol related/associated liver disease; ALD: alcohol-related liver disease.*

**Supplementary Figure S152**. Sensitivity analysis excluding low-quality studies (NOS ≤5) and conference abstracts comparing the pooled mean levels of GGT between patients with MetALD, compared to those with ALD.

*MetALD: metabolic and alcohol related/associated liver disease; ALD: alcohol-related liver disease.*

**Supplementary Figure S153**. Sensitivity analysis excluding low-quality studies (NOS ≤5) and conference abstracts comparing the pooled mean levels of platelet count (PLT) between patients with MetALD, compared to those with ALD.

*MetALD: metabolic and alcohol related/associated liver disease; ALD: alcohol-related liver disease.*

**Supplementary Figure S154**. Sensitivity analysis excluding low-quality studies (NOS ≤5) and conference abstracts comparing the pooled mean levels of estimated glomerular filtration rate (eGFR) between patients with MetALD, compared to those with MASLD.

*MetALD: metabolic and alcohol related/associated liver disease; ALD: alcohol-related liver disease.*

**Supplementary Figure S155**. Sensitivity analysis excluding low-quality studies (NOS ≤5) and conference abstracts comparing the pooled mean levels of systolic blood pressure (SBP) between patients with MetALD, compared to those with ALD.

*MetALD: metabolic and alcohol related/associated liver disease; ALD: alcohol-related liver disease.*

**Supplementary Figure S156**. Sensitivity analysis excluding low-quality studies (NOS ≤5) and conference abstracts comparing the pooled mean levels of diastolic blood pressure (DBP) between patients with MetALD, compared to those with ALD.

*MetALD: metabolic and alcohol related/associated liver disease; ALD: alcohol-related liver disease***.**

**Supplementary Figure S157**. Sensitivity analysis excluding low-quality studies (NOS ≤5) and conference abstracts comparing the pooled mean levels of high-density lipoprotein (HDL-C) between patients with MetALD, compared to those with ALD.

*MetALD: metabolic and alcohol related/associated liver disease; ALD: alcohol-related liver disease***.**

**Supplementary Figure S158**. Sensitivity analysis excluding low-quality studies (NOS ≤5) and conference abstracts comparing the pooled mean levels of low-density lipoprotein cholesterol (LDL-C) between patients with MetALD, compared to those with ALD.

*MetALD: metabolic and alcohol related/associated liver disease; ALD: alcohol-related liver disease***.**

**Supplementary Figure S159**. Sensitivity analysis excluding low-quality studies (NOS ≤5) and conference abstracts comparing the pooled mean levels of triglycerides (TG) between patients with MetALD, compared to those with ALD.

*MetALD: metabolic and alcohol related/associated liver disease; ALD: alcohol-related liver disease***.**

**Supplementary Figure S160**. Sensitivity analysis excluding low-quality studies (NOS ≤5) and conference abstracts comparing the pooled mean levels of total cholesterol between patients with MetALD, compared to those with ALD.

*MetALD: metabolic and alcohol related/associated liver disease; ALD: alcohol-related liver disease***.**

**Supplementary Figure S161**. Sensitivity analysis excluding low-quality studies (NOS ≤5) and conference abstracts comparing the pooled mean levels of fasting glucose between patients with MetALD, compared to those with ALD.

*MetALD: metabolic and alcohol related/associated liver disease; ALD: alcohol-related liver disease***.**

**Supplementary Figure S162**. Sensitivity analysis excluding low-quality studies (NOS ≤5) and conference abstracts comparing the pooled mean levels of HbA1c between patients with MetALD, compared to those with ALD.

*MetALD: metabolic and alcohol related/associated liver disease; ALD: alcohol-related liver disease***.**

**Supplementary Figure S163**. Sensitivity analysis excluding low-quality studies (NOS ≤5) and conference abstracts comparing the pooled mean levels of body mass index (BMI) between patients with MetALD, compared to those with ALD.

*MetALD: metabolic and alcohol related/associated liver disease; ALD: alcohol-related liver disease***.**

**Supplementary Figure S164**. Sensitivity analysis excluding low-quality studies (NOS ≤5) and conference abstracts comparing the pooled mean levels of waist circumference (WC) between patients with MetALD, compared to those with ALD.

*MetALD: metabolic and alcohol related/associated liver disease; ALD: alcohol-related liver disease***.**

**Supplementary Figure S165**. Sensitivity analysis excluding low-quality studies (NOS ≤5) and conference abstracts comparing the pooled mean levels of fibrosis-4 (FIB-4) score between patients with MetALD, compared to those with ALD.

*MetALD: metabolic and alcohol related/associated liver disease; ALD: alcohol-related liver disease***.**

**Supplementary Figure S166.** Funnel plot for studies comparing the age between the patients with metabolic and alcohol related/associated liver disease (MetALD) and those with metabolic dysfunction-associated steatotic liver disease (MASLD). Egger's test p-value = 0.7049.

**Supplementary Figure S167.** Funnel plot for studies comparing the aspartate aminotransferase (AST) levels between the patients with metabolic and alcohol related/associated liver disease (MetALD) and those with metabolic dysfunction-associated steatotic liver disease (MASLD). Egger's test p-value=0.6655.

**Supplementary Figure S168.** Funnel plot for studies comparing the alanine aminotransferase (ALT) levels between the patients with metabolic and alcohol related/associated liver disease (MetALD) and those with metabolic dysfunction-associated steatotic liver disease (MASLD). Egger's test p-value=0.7695.

**Supplementary Figure S169.** Funnel plot for studies comparing the Gamma-Glutamyl Transferase (GGT) levels between the patients with metabolic and alcohol related/associated liver disease (MetALD) and those with metabolic dysfunction-associated steatotic liver disease (MASLD). Egger's test p-value = 0.4599.

**Supplementary Figure S170.** Funnel plot for studies comparing the fasting glucose levels between the patients with metabolic and alcohol related/associated liver disease (MetALD) and those with metabolic dysfunction-associated steatotic liver disease (MASLD). Egger's test p-value = 0.9566.

**Supplementary Figure S171.** Funnel plot for studies comparing the Hemoglobin A1c, glycated hemoglobin (HbA1c) levels between the patients with metabolic and alcohol related/associated liver disease (MetALD) and those with metabolic dysfunction-associated steatotic liver disease (MASLD). Egger's test p-value = 0.1761

**Supplementary Figure S172.** Funnel plot for studies comparing the High density lipoprotein (HDL-C) levels between the patients with metabolic and alcohol related/associated liver disease (MetALD) and those with metabolic dysfunction-associated steatotic liver disease (MASLD). Egger's test p-value = 0.6167.

**Supplementary Figure S173.** Funnel plot for studies comparing the Low density lipoprotein cholesterol (LDL-C) levels between the patients with metabolic and alcohol related/associated liver disease (MetALD) and those with metabolic dysfunction-associated steatotic liver disease (MASLD). Egger's test p-value = 0.1809.

**Supplementary Figure S174.** Funnel plot for studies comparing the triglycerides (TG) levels between the patients with metabolic and alcohol related/associated liver disease (MetALD) and those with metabolic dysfunction-associated steatotic liver disease (MASLD). Egger's test p-value = 0.4097.

**Supplementary Figure S175.** Funnel plot for studies comparing the total cholesterol (CHOL) levels between the patients with metabolic and alcohol related/associated liver disease (MetALD) and those with metabolic dysfunction-associated steatotic liver disease (MASLD). Egger's test p-value = 0.0663.

**Supplementary Figure S176.** Funnel plot for studies comparing the body mass index (BMI) between the patients with metabolic and alcohol related/associated liver disease (MetALD) and those with metabolic dysfunction-associated steatotic liver disease (MASLD). Egger's test p-value = 0.5978.

**Supplementary Figure S177.** Funnel plot for studies comparing the waist circumference (WC) between the patients with metabolic and alcohol related/associated liver disease (MetALD) and those with metabolic dysfunction-associated steatotic liver disease (MASLD). Egger's test p-value = 0.2052.

**Supplementary Figure S178.** Funnel plot for studies comparing the platelet count (PLT) between the patients with metabolic and alcohol related/associated liver disease (MetALD) and those with metabolic dysfunction-associated steatotic liver disease (MASLD). Egger's test p-value = 0.2134.

**Supplementary Figure S179.** Funnel plot for studies comparing the fibrosis-4 (FIB-4) score between the patients with metabolic and alcohol related/associated liver disease (MetALD) and those with metabolic dysfunction-associated s teatotic liver disease (MASLD). Egger's test p-value = 0.1136.

**Supplementary Figure S180.** Funnel plot for studies comparing the age between the patients with metabolic and alcohol related/associated liver disease (MetALD) and those with alcohol related liver disease (ALD). Egger's test p-value = 0.4883.

**Supplementary Figure S181.** Funnel plot for studies comparing the aspartate aminotransferase (AST) levels between the patients with metabolic and alcohol related/associated liver disease (MetALD) and those with alcohol related liver disease (ALD). Egger's test p-value = 0.1281.

**Supplementary Figure S182.** Funnel plot for studies comparing the alanine aminotransferace (ALT) levels between the patients with metabolic and alcohol related/associated liver disease (MetALD) and those with alcohol related liver disease (ALD). Egger's test p-value = 0.1204.

**Supplementary Figure S183.** Funnel plot for studies comparing the Gamma-Glutamyl Transferase (GGT) levels between the patients with metabolic and alcohol related/associated liver disease (MetALD) and those with alcohol related liver disease (ALD). Egger's test p-value = 0.5784.

**Supplementary Figure S184.** Funnel plot for studies comparing the Hemoglobin A1c, glycated hemoglobin (HbA1c) levels between the patients with metabolic and alcohol related/associated liver disease (MetALD) and those with alcohol related liver disease (ALD). Egger's test p-value = 0.5161.

**Supplementary Figure S185.** Funnel plot for studies comparing the High density lipoprotein (HDL-C) levels between the patients with metabolic and alcohol related/associated liver disease (MetALD) and those with alcohol related liver disease (ALD). Egger's test p-value = 0.0728.

**Supplementary Figure S186.** Funnel plot for studies comparing the Low density lipoprotein cholesterol (LDL-C) levels between the patients with metabolic and alcohol related/associated liver disease (MetALD) and those with alcohol related liver disease (ALD). Egger's test p-value = 0.2927.

**Supplementary Figure S187.** Funnel plot for studies comparing the triglycerides (TG) levels between the patients with metabolic and alcohol related/associated liver disease (MetALD) and those with alcohol related liver disease. (ALD). Egger's test p-value = 0.3627.

**Supplementary Figure S188.** Funnel plot for studies comparing the body mass index (BMI) between the patients with metabolic and alcohol related/associated liver disease (MetALD) and those with alcohol related liver disease. (ALD). Egger's test p-value = 0.9440.

**Supplementary Figure S189.** Funnel plot for studies comparing the waist circumference (WC) between the patients with metabolic and alcohol related/associated liver disease (MetALD) and those with alcohol related liver disease. (ALD). Egger's test p-value = 0.2824.

**Suppl. Table 1a.** Published studies regarding the characteristics and laboratory findings of patients with subtypes of SLD (MASLD, MetALD and ALD).

| **First author,** **Publication year, Country, Study design (Ref.)** | **Newcastle-Ottawa Scale*** | **Number of patients**  **n/n/n^#^ (%),**  **type of cohort** | **Diagnosis of SLD, Evaluation of alcohol consumption** | **Male sex,**  **n/n/n**  **(%)** | **AST, mean (IU/L)** | **ALT,** **mean (IU/L)** | **GGT,** **mean (IU/L)** | **PLTs,** **mean** (**10⁹/L)** | **eGFR,** **mean (mL/min/1.73 m²)** |
| --- | --- | --- | --- | --- | --- | --- | --- | --- | --- |
| Chen L et al, 2024, China, retrospective (20) | (3/1/0) | 207/27/NA (57/7/NA)  hospital-based | LB, NA | 97/25/NA (47/93/NA) | NA/NA/NA | NA/NA/NA | 61.54/102.58/NA | NA/NA/NA | NA/NA/NA |
| Lee HH et al, 2024, Korea, retrospective (21) | (4/2/3) | 2686615/430993/NA (82/13/NA)  population-based | Fatty liver index, ΝΑ | 1914704/403243/NA (71/94/NA) | 25/27/NA | 28/30/NA | NA/NA/NA | NA/NA/NA | 85.9/89.4/NA |
| Lee CM et al, 2023, Korea, retrospective (22) | (4/1/0) | 745/143/68 (75/14/7), hospital-based | MRI, questionnaire | 642/131/NA (86/92/NA) | 33.85/34.52/NA | 41.34/39.41/NA | NA/NA/NA | 254.95/245.88/NA | NA/NA/NA |
| Israelsen et al, 2024, Denmark, retrospective (23) | (4/2/3) | 153/76/92 (48/24/29)  hospital-based | LB, standardised interview | 122/57/78 (80/75/85) | NA/NA/NA | NA/NA/NA | NA/NA/NA | NA/NA/NA | NA/NA/NA |
| Han et al, 2024, Korea, retrospective (24) | (4/1/3) | 7111/1399/NA (79/15/NA)  population-based | Liver fat score/ Fatty liver index, standardised interview | 3051/1129/NA (43/81/NA) | 26.1/34.3/NA | 30.40/38.30/NA | 40.70/93.3/NA | 259.2/250.5/NA | 89/95.6/NA |
| Choe et al, 2024, Korea, retrospective (25) | (4/1/3) | 3642/424/207 (85/10/5)  population-based | Fatty liver index, NA | 1871/400/199 (51/94/96) | 30/35/39 | 33/36/40 | 30.04/56.43/79.55 | 275/263/259 | NA/NA/NA |
| Miwa et al, 2024, Japan, retrospective (26) | (4/1/0) | 177/12/5 (86/6/2)  population-based | US, questionnaire | 124/10/4 (70/83/80) | 21.53/25.53/27.99 | 27.26/33.14/31.35 | 32.26/74.38/89.87 | NA/NA/NA | NA/NA/NA |
| Hydes et al, 2024, UK biobank, retrospective (27) | (4/1/3) | 220771/76385/39100 (66/23/12)  population-based | Hepatic steatosis index, NA | 94931/39720/26979 (43/52/69) | 24/25/NA | 21/22/NA | 27/31/NA | NA/NA/NA | NA/NA/NA |
| Kim et al, 2024, Korea, retrospective (28) | (3/1/0) | 5525/473/NA (90/8/NA)  population-based | US, standardised interview | 4788/468/NA (87/99/NA) | 26.3/31/NA | 31.8/34.7/NA | 46.1/87.7/NA | NA/NA/NA | 84.6/88.4/NA |
| Chen YT et al, 2024, Taiwan, retrospective (29) | (4/1/3) | 104843/9254/11408 (81/7/9)  population-based | US, questionnaire | 59842/8318/9879 (57/90/87) | 25.95/28.08/30.53 | 35.92/39.38/39.23 | NA/NA/NA | 252.66/244.74/243.43 | NA/NA/NA |
| Park et al, 2024, Korea, retrospective (30) | (3/1/0) | 213/31/NA (25/4/NA)  hospital-based | MRI, NA | 142/29/NA (67/94/NA) | 29.10/35.10/NA | 29.26/35.11/NA | 29.1/65.98/NA | NA/NA/NA | NA/NA/NA |
| Mori et al, 2024, Japan, retrospective (31) | (4/1/0) | 4227/795/324 (75/14/6)  hospital-based | US, self-administered questionnaire | 3488/720/313 (83/91/97) | 24/26.02/29.07 | 32.01/32.02/34.14 | 44.04/76.32/103.82 | 241/230/230 | 82.6/84.5/86.7 |
| Tamaki et al, 2024, Japan, retrospective (32) | (3/1/3) | 620832/108494/32074 (82/14/4)  population-based | Fatty liver index, questionnaire data | 517232/97183/27733 (83/90/86) | 24/26/28 | 31/30/29 | 47/72/90.02 | NA/NA/NA | NA/NA/NA |
| Wu et al, 2024, China, retrospective (33) | (4/1/0) | 2240/537/NA (77/18/NA)  population-based | CAP, questionnaire data | 1581/483/NA (71/90/NA) | 25/25/NA | 34/35/NA | 41/49/NA | NA/NA/NA | NA/NA/NA |
| Kwak et al, 2024, USA, retrospective (34) | (4/1/3) | 3774/167/NA (88/4/NA) population-based | US, standardized questionnaire | 1876/132/NA (50/79/NA) | 23.2/27.9/NA | 22.2/26.8/NA | NA/NA/NA | 275.8/254.1/NA | NA/NA/NA |
| Männistö et al, 2024, Finland, retrospective (35) | (3/1/3) | 8239/1003/437 (79/10/4)  population-based | Fatty liver index, NA | 3681/683/349 (45/68/80) | 30.39/38.49/44.14 | 33.79/45.83/50.09 | 49.99/64.86/87.92 | NA/NA/NA | NA/NA/NA |
| Marti-Aguado et al, 2024, Spain, retrospective (36) | (3/2/3) | 2227/76/NA (93/3/NA)  population-based | CAP, questionnaire | 1343/34/NA (60/45/NA) | 27/33/NA | 31/35/NA | 34.59/54.08/NA | NA/NA/NA | NA/NA/NA |
| Oh R et al, 2025, Korea, retrospective (37) | (3/1/3) | 430081/51032/13022 (87/10/3)  population-based | Fatty liver index, questionnaire | 280319/47452/12376 (65/93/95) | 29/34.4/39.20 | 32.50/34.9/37.60 | NA/NA/NA | NA/NA/NA | 85.7/89.9/90.2 |
| Park Y et al, 2024, Korea, retrospective (38) | (4/1/3) | 1102225/166359/NA (78/12/NA), population-based | Fatty liver index, ΝΑ | 736659/154064/NA (67/93/NA) | 25/27/NA | 26/28/NA | 37/66/NA | NA/NA/NA | NA/NA/NA |
| Baek et al, 2024, Korea, retrospective (39) | (3/1/2) | 6942/6694/4751 (37/36/25), population-based | Fatty liver index, questionnaire | 5981/6379/4625 (86/95/97) | 26.4/28.5/30.50 | 35.2/36.1/37.40 | 51.7/73.8/90.30 | NA/NA/NA | 88/86.1/86.5 |
| Hoang et al, 2025, Korea, retrospective (40) | (3/1/2) | 5258/928/979 (19/3/3), population-based | Fatty liver index, questionnaire | NA/NA/NA (NA/NA/NA) | 30.6/32.7/30.70 | 37.1/38.1/36 | 70.9/82.1/57.30 | NA/NA/NA | NA/NA/NA |
| Oh JH et al, 2024, Korea, retrospective (41) | (4/1/3) | 2652/462/NA (73/13/NA), population-based | US, ΝΑ | 2383/425/NA (90/92/NA) | 33.8/31.5/NA | 41.5/37.8/NA | 71.3/66.1/NA | 249.8/245.9/NA | NA/NA/NA |
| Kamada et al, 2024, Japan, retrospective (42) | (3/1/2) | 20922/7841/918 (68/25/3), population-based | US, ΝΑ | 13007/7005/802 (62/89/87) | 25.4/28.5/35.40 | 32.6/34.4/38.20 | 42.1/75.9/123 | 238/229/232 | 75.7/76.9/81.1 |
| Yoon et al, 2024, Korea, retrospective (43) | (3/1/2) | 3497/411/107  (87/10/3), population-based | Hepatic steatosis index, ΝΑ | 1677/351/68 (48/85/64) | 28.1/37.1/37.10 | 38.2/48.2/48.20 | NA/NA/ΝΑ | 265.10/258.9/255.20 | NA/NA/NA |
| John et al, 2025, USA, retrospective (44) | (4/1/3) | 299259/99163/38552 (68/23/9), population-based | Radiology, AUDIT-C score | 270944/91669/35903 (91/92/93) | 31.3/38.9/53.30 | 41.3/47.8/47.80 | NA/NA/NA | 212.3/210.4/202.40 | NA/NA/NA |
| Wang et al, 2025, Taiwan, retrospective (45) | (3/1/3) | 7316/209/79 (94/3/1), population-based | US, questionnaire | 2898/173/65 (40/83/82) | 26.76/31.28/33.63 | 29.65/38.32/33.35 | 28.75/66.51/79.03 | NA/NA/NA | NA/NA/NA |
| Lee DY et al, 2025, Korea, retrospective (46) | (2/1/2) | 142065/14176/NA (30/3/NA), population-based | US, NA | 105370/12916/NA (74/91/NA) | 27.2/32.1/NA | 35.5/38.9/NA | NA/NA/NA | NA/NA/NA | NA/NA/NA |
| Vaz et al, 2025, Sweden, retrospective (47) | (4/1/3) | 4229/1294/1976 (56/17/26), hospital-based | ICD-10/ phosphatidylethanol, NA | 1927/771/1277 (46/60/65) | 25.3/27.74/47.13 | 28.92/30.83/38.64 | 33.73/43.96/150.81 | NA/NA/NA | NA/NA/NA |
| Spencer-Sandino et al, 2025, Chile, retrospective (48) | (3/1/3) | 3895/77/53 (95/2/1), populayion-based | US, NA | 1760/51/45 (45/66/85) | 30.8/35.9/53.40 | 40.5/48/53.40 | 39.5/76/55.40 | NA/NA/NA | NA/NA/NA |
| Burnside et al, 2025, Canada, retrospective (49) | (2/1/3) | 8430/666/144 (91/7/2), population-based | Serology, questionnaire | 5455/500/NA (65/75/NA) | NA/NA/NA | 26.01/NA/NA | NA/NA/NA | NA/NA/NA | NA/NA/NA |
| Ong et al, 2025, USA, retrospective (50) | (3/1/2) | 14152/3850/2508 (69/19/12), population-based | Fatty liver index, NA | NA/NA/NA (NA/NA/NA) | 23/23/25.01 | 23/24.02/28.03 | 25/27.03/34.05 | 249/256.02/254.01 | NA/NA/NA |
| Younossi et al, 2025, Multicenter, retrospective (51) | (2/1/2) | 8638/170/0 (98/2/0)  hospital-based | LB, NA | NA/NA/NA (NA/NA/NA) | NA/NA/NA | NA/NA/NA | NA/NA/NA | NA/NA/NA | NA/NA/NA |
| Gratacos-Gines et al, 2025, Spain, retrospective (52) | (2/1/2) | 1639/216/64 (85/11/3), population-based | Fatty liver index, NA | NA/NA/NA (NA/NA/NA) | NA/NA/NA | NA/NA/NA | NA/NA/NA | NA/NA/NA | NA/NA/NA |

*# All n/n/n results: number of patients with MASLD/MetALD/ALD*

**In parenthesis for Newcastle-Ottawa Scale (selection [max 4]/comparability [max 2]/outcome [max 3]).*

***Abbreviations****: SLD: steatotic liver disease; MetALD: metabolic and alcohol related/associated liver disease; MASLD: metabolic dysfunction-associated steatotic liver disease; ALD: alcohol-related liver disease; NA: not applicable; AST: aspartate transaminase; ALT: alanine aminotransferase; GGT: gamma-glutamyl transferase; PLT: platelet count; eGFR: estimated glomerular filtration rate; US: ultrasound, LB: liver biopsy; MRI:* *Magnetic resonance imaging; CAP:* *controlled attenuation parameter.*

**Suppl. Table 1b.** Published studies regarding the metabolic parameters of patients with subtypes of SLD (MASLD, MetALD and ALD).

| **First author, Publication year, Country, Study design (Ref.)** | **Number of patients**  **n/n/n^#^** | **BMI, mean (kg/m²)** | **WC, mean (cm)** | **Glucose, mean (mg/dL)** | **Insulin, mean**  **(mIU/L)** | **HbA1c, mean (%)** | **Total Cholesterol, mean (mg/dL)** | **LDL-C** **mean (mg/dL)** | **HDL-C** **mean (mg/dL)** | **TG** **mean (mg/dL)** | **HOMA-IR, mean** |
| --- | --- | --- | --- | --- | --- | --- | --- | --- | --- | --- | --- |
| Chen et al, 2024, China, retrospective (20) | 207/27/NA | NA/NA/NA | NA/NA/NA | NA/NA/NA | NA/NA/NA | NA/NA/NA | NA/NA/NA | NA/NA/NA | NA/NA/NA | NA/NA/NA | NA/NA/NA |
| Lee HH et al, 2024, Korea, retrospective (21) | 2686615/430993/NA | 26.10/25.60/NA | 88/87/NA | 97/98/NA | NA/NA/NA | NA/NA/NA | 204/202/NA | 117/109/NA | 48/51/NA | NA/181/NA | NA/NA/NA |
| Lee CM et al, 2023, Korea, retrospective (22) | 745/143/68 | 26.38/26.50/NA | NA/NA/NA | NA/NA/NA | NA/NA/NA | NA/NA/NA | NA/NA/NA | 128.95/133.94/NA | 48.68/50.02/NA | 175.89/179.02/NA | NA/NA/NA |
| Israelsen et al, 2024, Denmark, retrospective (23) | 153/76/92 | 28/29/NA | NA/NA/NA | NA/NA/NA | NA/NA/NA | NA/NA/NA | NA/NA/NA | NA/NA/NA | NA/NA/NA | NA/NA/NA | NA/NA/NA |
| Han et al, 2024, Korea, retrospective (24) | 7111/1399/NA | 26/26.30/NA | 88.60/90.50/NA | 106.60/105.50/NA | 14.30/13.1/NA | 6.70/6.3/NA | 196/198.30/NA | 120.10/112.60/NA | 43/44.90/NA | 181.60/263.70/NA | 3.90/3.5/NA |
| Choe et al, 2024, Korea, retrospective (25) | 3642/424/207 | 26.70/25.60/25.60 | 89.20/87.40/87.60 | 92/98/102 | NA/NA/NA | 5.8/5.8/5.8 | 203/202/201 | 125/120/120 | 43/45/47 | 211/227/250 | 2/1.80/NA |
| Miwa et al, 2024, Japan, retrospective (26) | 177/12/5 | 26.07/25.8/25.72 | 88.07/89.23/98.27 | NA/NA/NA | NA/NA/NA | 5.50/5.4/5.28 | NA/NA/NA | 125.22/128.36/120.18 | 47.22/50.48/68.72 | 171.46/237.08/225.80 | NA/NA/NA |
| Hydes et al, 2024, UK biobank, retrospective (27) | 220771/76385/39100 | 27.90/27.40/27.20 | 92/NA/94 | NA/NA/NA | NA/NA/NA | NA/NA/NA | 216.60/NA/NA | 139/NA/NA | 50.30/NA/NA | 141.70/NA/NA | NA/NA/NA |
| Kim et al, 2024, Korea, retrospective (28) | 5525/473/NA | 25.60/26.30/NA | NA/NA/NA | NA/NA/NA | NA/NA/NA | NA/NA/NA | NA/NA/NA | NA/NA/NA | NA/NA/NA | NA/NA/NA | NA/NA/NA |
| Chen YT et al, 2024, Taiwan, retrospective (29) | 104843/9254/11408 | 26.08/26.13/25.99 | NA/NA/NA | NA/NA/NA | NA/NA/NA | NA/NA/NA | NA/NA/NA | NA/NA/NA | NA/NA/NA | NA/NA/NA | NA/NA/NA |
| Park et al, 2024, Korea, retrospective (30) | 213/31/NA | NA/NA/NA | NA/NA/NA | 105.13/102.70/NA | NA/NA/NA | 5.915.72/NA | NA/NA/NA | NA/NA/NA | 46.06/48.46/NA | 137.33/200.15/NA | 2.61/2.99/NA |
| Mori et al, 2024, Japan, retrospective (31) | 4227/795/324 | 26.20/25.40/25 | 91.40/90.70/90.50 | ΝΑ/102.7/105 | NA/NA/NA | 5.70/5.6/5.6 | 213/212/214 | 132/123/121 | 51/57/59 | 131.06/143.14/145.77 | NA/NA/NA |
| Tamaki et al, 2024, Japan, retrospective (32) | 620832/108494/32074 | 26.80/25.80/25.20 | NA/NA/NA | NA/NA/NA | NA/NA/NA | 5.60/5.5/5.4 | NA/NA/NA | NA/NA/118 | NA/NA/59 | NA/NA/NA | NA/NA/NA |
| Wu et al, 2024, China, retrospective (33) | 2240/537/NA | 26.10/26.40/NA | NA/NA/NA | NA/NA/NA | NA/NA/NA | NA/NA/NA | 208/NA/NA | 129/117.20/NA | 51/49.50/NA | NA/NA/NA | 2.50/2.6/NA |
| Kwak et al, 2024, USA, retrospective (34) | 3774/167/NA | 29.80/28.50/NA | NA/NA/NA | 105.40/106.60/NA | NA/NA/NA | 5.59/5.39/NA | 211/208.30/NA | NA/NA/NA | 45.30/54.10/NA | 187/160.90/NA | NA/NA/NA |
| Männistö et al, 2024, Finland, retrospective (35) | 8239/1003/437 | 31.70/28.80/28.68 | 105.49/99.70/100.82 | NA/NA/NA | NA/NA/NA | NA/NA/NA | NA/NA/NA | NA/NA/NA | NA/NA/NA | NA/NA/NA | NA/NA/NA |
| Marti-Aguado et al, 2024, Spain, retrospective (36) | 2227/76/NA | 33.90/30.29/NA | 112/99.13/NA | 107.99/85.73/NA | NA/NA/NA | NA/NA/NA | 191.01/209.39/NA | NA/123.93/NA | 47/54.40/NA | 184.99/128.61/NA | NA/NA/NA |
| Oh R et al, 2025, Korea, retrospective (37) | 430081/51032/13022 | 26.50/25.70/25.70 | 88.50/88.10/88.40 | 107.50/110/113.70 | NA/NA/NA | NA/NA/NA | 204.60/203.90/202.30 | 118.60/111.10/107.30 | 49.40/53.30/54.40 | 190.60/212.70/220.60 | NA/NA/NA |
| Park Y et al, 2024, Korea, retrospective (38) | 1102225/166359/NA | 26.10/25.50/NA | 88/87/NA | 99/101/NA | NA/NA/NA | NA/NA/NA | 204/202/NA | NA/NA/NA | 48/51/NA | 169/181/NA | NA/NA/NA |
| Baek et al, 2024, Korea, retrospective (39) | 6942/6694/4751 | 26.30/26.10/26 | 89.50/89.20/89.50 | 97.90/96.70/99.60 | NA/NA/NA | 5.97/5.83/5.4 | NA/NA/NA | 124/117/116 | 46.60/48.30/48.80 | 188/208/217 | 1.37/1.34/NA |
| Hoang et al, 2025, Korea, retrospective (40) | 5258/928/979 | 26.20/26.20/25.90 | NA/NA/NA | 97.10/99.70/97.40 | NA/NA/NA | NA/NA/NA | 210.40/211.50/209.20 | 133.10/132.30/134.60 | 50.50/51.10/49.90 | 183.40/200/165.30 | NA/NA/NA |
| Oh JH et al, 2024, Korea, retrospective (41) | 2652/462/NA | 26.40/26.20/NA | 90.20/90.20/NA | 104.10/103.90/NA | NA/NA/NA | 5.90/5.9/NA | 204.50/206.70/NA | 122.20/124.90/NA | 48.40/49.60/NA | 181.30/183/NA | NA/NA/NA |
| Kamada et al, 2024, Japan, retrospective (42) | 20922/7841/918 | 26.10/26/26.10 | 90.20/91/92 | 106/109/111 | NA/NA/NA | 5.99/5.9/5.86 | NA/NA/NA | 131/126/120 | 52.40/55/57.40 | 140/164/191 | NA/NA/NA |
| Yoon et al, 2024, Korea, retrospective (43) | 3497/411/107 | 28.40/28.60/28.80 | 94.80/97.10/97.40 | 111.10/119.30/119.30 | NA/NA/NA | 6.20/6.1/6.2 | 191.80/195.80/200.10 | 120.40/116/112.50 | 46.50/48.40/51.40 | 162.10/210.50/258.10 | NA/NA/NA |
| John et al, 2025, USA, retrospective (44) | 299259/99163/38552 | 32.40/31.10/29.60 | NA/NA/NA | NA/NA/NA | NA/NA/NA | 6.60/6.2/6 | NA/NA/NA | NA/NA/NA | NA/NA/NA | NA/NA/NA | NA/NA/NA |
| Wang et al, 2025, Taiwan, retrospective (45) | 7316/209/79 | 26.16/26.94/26.63 | 88.61/92.76/92.80 | 102.42/105.89/NA | NA/NA/NA | 6.12/6.05/5.98 | 200.80/200.83/199.35 | 125.59/116.11/110.47 | 49.73/49.71/52.76 | 156.84/234.95/245.77 | NA/NA/NA |
| Lee DY et al, 2025, Korea, retrospective (46) | 142065/14176/NA | 26.50/26.90/NA | 90.20/92.20/NA | 102.50/106.50/NA | 10.40/9.8/NA | 5.83/5.82/NA | 202/206.30/NA | 133.60/131.90/NA | 51/52.90/NA | 160.70/192.60/NA | 2.71/2.64/NA |
| Vaz et al, 2025, Sweden, retrospective (47) | 4229/1294/1976 | 31.99/31/29 | NA/NA/NA | NA/NA/NA | NA/NA/NA | NA/NA/NA | NA/NA/NA | NA/NA/NA | NA/NA/NA | NA/NA/NA | NA/NA/NA |
| Spencer-Sandino et al, 2025, Chile, retrospective (48) | 3895/77/53 | NA/NA/NA | NA/NA/NA | NA/NA/NA | NA/NA/NA | NA/NA/NA | NA/NA/NA | NA/NA/NA | NA/NA/NA | NA/NA/NA | NA/NA/NA |
| Burnside et al, 2025, Canada, retrospective (49) | 8430/666/144 | 30/29.02/NA | 103/101.98/NA | NA/NA/NA | NA/NA/NA | 5.70/5.6/NA | NA/NA/NA | NA/NA/NA | 42.59/50.30/NA | 203.83/195.16/NA | NA/NA/NA |
| Ong et al, 2025, USA, retrospective (50) | 14152/3850/2508 | 32.60/32.84/32.09 | 109.40/108.7/108.01 | 105/104.99/101.03 | NA/NA/NA | 5.70/5.5/5.5 | 197/202/204 | 115/120/120.01 | 45/47/43.02 | 146.03/141.07/154.13 | NA/NA/NA |
| Younossi et al, 2025, Multicenter, retrospective (51) | 8638/170/0 | NA/NA/NA | NA/NA/NA | NA/NA/NA | NA/NA/NA | NA/NA/NA | NA/NA/NA | NA/NA/NA | NA/NA/NA | NA/NA/NA | NA/NA/NA |
| Gratacos-Gines et al, 2025, Spain, retrospective (52) | 1639/216/64 | NA/NA/NA | NA/NA/NA | NA/NA/NA | NA/NA/NA | NA/NA/NA | NA/NA/NA | NA/NA/NA | NA/NA/NA | NA/NA/NA | NA/NA/NA |

*# All n/n/n results: number of patients with MASLD/MetALD/ALD*

***Abbreviations****:* *SLD: steatotic liver disease; MetALD: metabolic and alcohol related/associated liver disease; MASLD: metabolic dysfunction-associated steatotic liver disease; ALD: alcohol-related liver disease; NA: not applicable; BMI: body mass index, WC: waist circumference; HbA1c: glycated hemoglobin A1C;* *BMI: body mass index, WC: waist circumference; HbA1c: glycated hemoglobin A1C; LDL-C: low density lipoprotein cholesterol; HDL-C: high density lipoprotein cholesterol; TG: triglycerides; HOMA-IR: Homeostasis Model Assessment of Insulin Resistance.*

**Suppl. Table 1c.** **Published studies regarding the non-invasive tests of patients with subtypes of SLD (MASLD, MetALD and ALD).**

| **First author, Publication year, Country, Study design (Ref.)** | **Number of patients**  **n/n/n^#^** | **FLI, mean** | **FIB-4, mean** | **NFS, mean** | **LSM (MRE), mean** **(kPa)** |
| --- | --- | --- | --- | --- | --- |
| Chen et al, 2024, China, retrospective (20) | 207/27/NA | NA/NA/NA | 0.96/NA/NA | NA/NA/NA | 7.65/7.31/NA |
| Lee HH et al, 2024, Korea, retrospective (21) | 2686615/430993/NA | NA/NA/NA | NA/NA/NA | NA/NA/NA | NA/NA/NA |
| Lee CM et al, 2023, Korea, retrospective (22) | 745/143/68 | NA/NA/NA | 1.11/1.23/NA | NA/2.48/NA | NA (2.2)/NA (2.3)/NA |
| Israelsen et al, 2024, Denmark, retrospective (23) | 153/76/92 | NA/NA/NA | NA/NA/NA | NA/NA/NA | 10.01/8.09/NA |
| Han et al, 2024, Korea, retrospective (24) | 7111/1399/NA | 45.5/64.3/NA | 1.10/1.20/NA | 1.50/1.30/NA | NA/NA/NA |
| Choe et al, 2024, Korea, retrospective (25) | 3642/424/207 | NA/NA/NA | NA/NA/NA | NA/NA/NA | NA/NA/NA |
| Miwa et al, 2024, Japan, retrospective (26) | 177/12/5 | NA/NA/NA | 0.69/0.93/1.01 | NA/NA/NA | NA/NA/NA |
| Hydes et al, 2024, UK biobank, retrospective (27) | 220771/76385/39100 | NA/NA/NA | NA/NA/NA | NA/NA/NA | NA/NA/NA |
| Kim et al, 2024, Korea, retrospective (28) | 5525/473/NA | NA/NA/NA | NA/NA/NA | NA/NA/NA | NA/NA/NA |
| Chen YT et al, 2024, Taiwan, retrospective (29) | 104843/9254/11408 | NA/NA/NA | 0.92/0.94/1.06 | NA/NA/NA | NA/NA/NA |
| Park et al, 2024, Korea, retrospective (30) | 213/31/NA | NA/NA/NA | NA/NA/NA | NA/NA/NA | NA (9)/NA (8.2)/NA |
| Mori et al, 2024, Japan, retrospective (31) | 4227/795/324 | 52.5/60.7/NA | 0.91/1.06/1.18 | NA/NA/NA | NA/NA/NA |
| Tamaki et al, 2024, Japan, retrospective (32) | 620832/108494/32074 | NA/NA/NA | NA/NA/NA | NA/NA/NA | NA/NA/NA |
| Wu et al, 2024, China, retrospective (33) | 2240/537/NA | NA/NA/NA | 0.74/0.76/NA | NA/NA/NA | 5.70/5.70/NA |
| Kwak et al, 2024, USA, retrospective (34) | 3774/167/NA | NA/NA/NA | 0.95/1.21/NA | NA/NA/NA | NA/NA/NA |
| Männistö et al, 2024, Finland, retrospective (35) | 8239/1003/437 | NA/NA/NA | NA/NA/NA | NA/NA/NA | NA/NA/NA |
| Marti-Aguado et al, 2024, Spain, retrospective (36) | 2227/76/NA | NA/NA/NA | NA/NA/NA | NA/NA/NA | NA/NA/NA |
| Oh R et al, 2025, Korea, retrospective (37) | 430081/51032/13022 | 53.2/60.7/NA | NA/NA/NA | NA/NA/NA | NA/NA/NA |
| Park Y et al, 2024, Korea, retrospective (38) | 1102225/166359/NA | NA/NA/NA | NA/NA/NA | NA/NA/NA | NA/NA/NA |
| Baek et al, 2024, Korea, retrospective (39) | 6942/6694/4751 | NA/NA/NA | 0.92/0.87/0.95 | NA/NA/NA | NA/NA/NA |
| Hoang et al, 2025, Korea, retrospective (40) | 5258/928/979 | NA/NA/NA | NA/NA/NA | NA/NA/NA | NA/NA/NA |
| Oh JH et al, 2024, Korea, retrospective (41) | 2652/462/NA | NA/NA/NA | 1.10/1.10/NA | 2.03/1.98/NA | NA (2.3)/NA (2.4)/NA |
| Kamada et al, 2024, Japan, retrospective (42) | 20922/7841/918 | NA/NA/NA | 1.14/1.27/1.38 | NA/NA/NA | NA/NA/NA |
| Yoon et al, 2024, Korea, retrospective (43) | 3497/411/107 | NA/NA/NA | 1.02/0.94/1.03 | NA/NA/NA | NA/NA/NA |
| John et al, 2025, USA, retrospective (44) | 299259/99163/38552 | NA/NA/NA | NA/NA/NA | NA/NA/NA | NA/NA/NA |
| Wang et al, 2025, Taiwan, retrospective (45) | 7316/209/79 | 40.66/58.84/NA | 1.30/1.41/1.48 | NA/NA/1.52 | NA/NA/NA |
| Lee DY et al, 2025, Korea, retrospective (46) | 142065/14176/NA | NA/NA/NA | NA/NA/NA | 2.46/2.33/NA | NA/NA/NA |
| Vaz et al, 2025, Sweden, retrospective (47) | 4229/1294/1976 | NA/NA/NA | 1.07/1.19/2.49 | NA/NA/NA | NA/NA/NA |
| Spencer-Sandino et al, 2025, Chile, retrospective (48) | 3895/77/53 | NA/NA/NA | NA/NA/NA | NA/NA/NA | NA/NA/NA |
| Burnside et al, 2025, Canada, retrospective (49) | 8430/666/144 | NA/NA/NA | NA/NA/NA | NA/NA/NA | NA/NA/NA |
| Ong et al, 2025, USA, retrospective (50) | 14152/3850/2508 | NA/NA/NA | 1.01/0.83/0.73 | NA/NA/NA | NA/NA/NA |
| Younossi et al, 2025, Multicenter, retrospective (51) | 8638/170/0 | NA/NA/NA | 1.43/2.04/NA | NA/NA/NA | 10.90/13.50/NA |
| Gratacos-Gines et al, 2025, Spain, retrospective (52) | 1639/216/64 | NA/NA/NA | NA/NA/NA | NA/NA/NA | 5.30/5.60/7.10 |

*# All n/n/n results: number of patients with MASLD/MetALD/ALD*

***Abbreviations****: SLD: steatotic liver disease; MetALD: metabolic and alcohol related/associated liver disease; MASLD: metabolic dysfunction-associated steatotic liver disease; ALD: alcohol-related liver disease; NA: not applicable; FLI: Fatty Liver Index; FIB-4: Fibrosis-4 score; NFS: NAFLD fibrosis score; LSM: liver stiffness measurement*; *MRE:* *magnetic resonance elastography.*

**Suppl. Table 2.** Comparison of pooled mean levels of clinical and laboratory findings between patients with MetALD, compared to those with MASLD.

| **Variable (Units)** | **MetALD [pooled mean±SD]** | **MASLD [pooled mean±SD]** | **p value** |
| --- | --- | --- | --- |
| Age (years) | 49.7 ± 12 | 51.8 ± 15 | 0.005 |
| AST (IU/L) | 28.45 ± 15.5 | 25.59 ± 8.56 | < 0.0001 |
| ALT (IU/L) | 31.42 ± 16.21 | 29.1 ± 12.51 | < 0.0001 |
| GGT (IU/L) | 60.67 ± 20.92 | 39.17 ± 9.93 | < 0.0001 |
| eGFR (mL/min/1.73 m²) | 89.22 ± 8.77 | 85.81 ± 9.54 | 0.0072 |
| PLT count (10⁹/L) | 216.71 ± 48.2 | 226.34 ± 50.16 | 0.0008 |
| SBP (mmHg) | 129.03 ± 6.78 | 127.74 ± 6.87 | < 0.0001 |
| DBP (mmHg) | 80.25 ± 4.11 | 79.5 ± 4.22 | < 0.0001 |
| BMI (kg/m²) | 26.38 ± 2.47 | 26.66 ± 2.12 | 0.0093 |
| HbA1c (%) | 5.83 ± 0.82 | 5.91 ± 0.8 | < 0.0001 |
| Insulin (mIU/L) | 10.10 ± 6.79 | 10.59 ± 6.79 | 0.0039 |
| LDL-C (mg/dL) | 110.39 ± 16.69 | 118.1 ± 18.1 | 0.0010 |
| HDL-C (mg/dL) | 51.19 ± 5.19 | 48.22 ± 5.06 | < 0.0001 |
| TG (mg/dL) | 188.14 ± 83.3 | 173.41 ± 67.24 | 0.0127 |
| Total cholesterol (mg/dL) | 202.2± 14.7 | 203.9± 15.84 | 0.1710 |
| GLU (mg/dL) | 98.81 ± 10.68 | 99.99 ± 10.22 | 0.8347 |
| HOMA-IR (-) | 2.3 ± 1.66 | 2.69 ± 1.98 | 0.6427 |
| WC (cm) | 87.43 ± 2.75 | 88.28 ± 2.89 | 0.3879 |
|  |  |  |  |

*Abbreviations: MetALD: metabolic and alcohol related/associated liver disease; MASLD: metabolic dysfunction-associated steatotic liver disease; SD: standard deviation; AST: aspartate transaminase; ALT: alanine aminotransferase; GGT:* *gamma-glutamyl transferase; eGFR: estimated glomerular filtration rate; PLT: platelet; SPB: systolic blood pressure; DBP: diastolic blood pressure; BMI: body mass index; HbA1c:* *hemoglobin A1C; LDL-C: low density lipoprotein cholesterol; HDL-C: high density lipoprotein cholesterol; TG: triglycerides; GLU: fasting glucose; HOMA-IR:* *Homeostatic Model Assessment for Insulin Resistance; WC: waist circumference*

**Suppl. Table 3.** Comparison of pooled mean values of non-invasive tests (NITs) between patients with MetALD, compared to those with MASLD.

| **NITs (Units)** | **MetALD [pooled mean±SD]** | **MASLD [pooled mean±SD]** | **p value** |
| --- | --- | --- | --- |
| FLI (–) | 60.79 ± 18.64 | 52.87 ± 17.3 | < 0.0001 |
| FIB-4 (–) | 1.03 ± 0.64 | 1.0 ± 0.61 | 0.0235 |
| NFS (–) | -2.01 ± 1.19 | -2.27 ± 1.2 | 0.7022 |
| LSM (kPa) | 7.19 ± 4.82 | 9.22 ± 7.21 | 0.9816 |
| MRE (kPa) | 2.66 ± 0.56 | 2.68 ± 0.57 | 0.5393 |

*Abbreviations: MetALD: metabolic and alcohol related/associated liver disease; MASLD: metabolic dysfunction-associated steatotic liver disease; SD: standard deviation; FLI: fatty liver index; FIB-4: fibrosis-4 score; NFS: non-alcoholic fatty liver disease fibrosis score; LSM: liver stiffness measurement; MRE: magnetic resonance elastography.*

**Suppl. Table 4.** Comparison of pooled mean levels of clinical and laboratory findings between patients with MetALD, compared to those with ALD.

| **Variable (Units)** | **MetALD [pooled mean±SD]** | **ALD [pooled mean±SD]** | **p value** |
| --- | --- | --- | --- |
| Age (years) | 54.7 ± 14 | 53.6 ± 16 | 0.3429 |
| AST (IU/L) | 32.12 ± 26.07 | 39.3 ± 32.16 | 0.0007 |
| ALT (IU/L) | 37.53 ± 26.89 | 38.7 ± 28.15 | 0.0072 |
| GGT (IU/L) | 70.72 ± 31.03 | 89.52 ± 44.16 | 0.0323 |
| GFR (mL/min/1.73 m²) | 87.92 ± 16.5 | 88.78 ± 17.5 | 0.0775 |
| PLT count (10⁹/L) | 216.13 ± 48.04 | 214.46 ± 53.03 | 0.3255 |
| SBP (mmHg) | 128.5 ± 14.24 | 129.17 ± 14.47 | < 0.0001 |
| DBP (mmHg) | 80.97 ± 10 | 81.37 ± 10.12 | < 0.0001 |
| BMI (kg/m²) | 27.67 ± 3.96 | 27.22 ± 4.45 | 0.0084 |
| HbA1c (%) | 5.83 ± 0.82 | 5.76 ± 3.88 | 0.1140 |
| LDL-C (mg/dL) | 114.24 ± 39.38 | 112.52 ± 35.34 | 0.0452 |
| HDL-C (mg/dL) | 52.61 ± 13.87 | 51.9 ± 12.82 | 0.1983 |
| TG (mg/dL) | 202.37 ± 139.23 | 208.55 ± 129.13 | 0.2245 |
| Total cholesterol (mg/dL) | 203.92 ± 40.02 | 203.12 ± 15.63 | 0.9790 |
| GLU (mg/dL) | 89.93 ± 9.1 | 109.26 ± 32.93 | 0.1338 |
| HOMA-IR (–) | 1.37 ± 0.91 | 1.34 ± 1.49 | 0.4258 |
| WC (cm) | 89.86 ± 7.05 | 91.31 ± 7.15 | 0.3350 |
|  |  |  |  |

*Abbreviations: MetALD: metabolic and alcohol related/associated liver disease; ALD:* *alcohol-related liver disease; SD: standard deviation, AST: aspartate transaminase; ALT: alanine aminotransferase; GGT:* *gamma-glutamyl transferase; GFR: glomerular filtration rate; PLT: platelet; SPB: systolic blood pressure; DBP: diastolic blood pressure; BMI: body mass index; HbA1c:* *hemoglobin A1C; LDL-C: low density lipoprotein cholesterol; HDL-C: high density lipoprotein cholesterol; TG: triglycerides; GLU: fasting glucose; HOMA-IR:* *Homeostatic Model Assessment for Insulin Resistance; WC: waist circumference.*

**Suppl. Table 5.** Comparison of pooled mean levels of clinical and laboratory findings between Asian patients with MetALD, compared to those with MASLD.

| **Variable (Units)** | **MetALD [pooled mean±SD]** | **MASLD [pooled mean±SD]** | **p value** |
| --- | --- | --- | --- |
| Age (years) | 47.76±5.22 | 50.9±6.41 | 0.0024 |
| AST (IU/L) | 27.5±13.1 | 25.32±7.27 | <0.0001 |
| ALT (IU/L) | 30.31±12.59 | 28.75±10.78 | 0.0001 |
| GGT (IU/L) | 60.7±21.91 | 39.2±9.8 | <0.0001 |
| PLT count (10⁹/L) | 239.3±52.83 | 251.37±57.92 | <0.0001 |
| eGFR | 89.22±8.77 | 85.81±9.54 | 0.0072 |
| SBP (mmHg) | 129.01±6.75 | 127.73±6.83 | 0.0005 |
| DBP (mmHg) | 80.24±4.09 | 79.5±4.2 | 0.0004 |
| BMI (kg/m²) | 25.66±1.07 | 26.23±1.19 | 0.2165 |
| HbA1c (%) | 5.84±0.83 | 5.92±0.81 | 0.0003 |
| Insulin (mIU/L) | 10.1±6 | 10.59±6.79 | 0.0039 |
| LDL-C mg/dL) | 110.32±16.74 | 118.12±18.13 | <0.0001 |
| HDL-C (mg/dL) | 51.22±5.21 | 48.25±5.07 | <0.0001 |
| TG (mg/dL) | 188.88±84.06 | 173.44±67.77 | <0.0001 |
| Total cholesterol (mg/dL) | 202.25±14.2 | 203.98±15.87 | 0.5176 |
| GLU (mg/dL) | 99.96±10.25 | 98.78±10.71 | 0.011 |
| HOMA-IR(-) | 2.3±1.66 | 2.69±1.98 | 0.6427 |
| WC (cm) | 87.28±2.7 | 88.15±2.87 | 0.2825 |

*Abbreviations: MetALD: metabolic and alcohol related/associated liver disease; MASLD: metabolic dysfunction-associated steatotic liver disease; SD: standard deviation, AST: aspartate transaminase; ALT: alanine aminotransferase; GGT:* *gamma-glutamyl transferase; eGFR: estimated glomerular filtration rate; PLT: platelet; SPB: systolic blood pressure; DBP: diastolic blood pressure; BMI: body mass index; HbA1c:* *hemoglobin A1C; LDL-C: low density lipoprotein cholesterol; HDL-C: high density lipoprotein cholesterol; TG: triglycerides; GLU: fasting glucose; HOMA-IR:* *Homeostatic Model Assessment for Insulin Resistance; WC: waist circumference.*

**Suppl. Table 6.** Comparison of pooled mean values of non-invasive tests (NITs) between Asian patients with MetALD compared to those with MASLD.

| **NITs (Units)** | **MetALD [pooled mean±SD]** | **MASLD [pooled mean±SD]** | **p-value** |
| --- | --- | --- | --- |
| FIB-4 (–) | 1.04 ± 0.69 | 0.98 ± 0.58 | 0.0262 |

*Abbreviations: MetALD: metabolic and alcohol related/associated liver disease; MASLD: metabolic dysfunction-associated steatotic liver disease; FIB-4: fibrosis-4 score.*

**Suppl. Table 7.** Comparison of pooled mean levels of clinical and laboratory findings between non-Asian patients with MetALD, compared to those with MASLD.

| **Variable (Units)** | **MetALD [pooled mean±SD]** | **MASLD [pooled mean±SD]** | **p value** |
| --- | --- | --- | --- |
| Age (years) | 58.17±12.68 | 59.28±12.87 | 0.4834 |
| AST (IU/L) | 32.63±23.29 | 28.06±16.22 | 0.0002 |
| ALT (IU/L) | 36.31±26.87 | 32.4±23.02 | 0.0010 |
| GGT (IU/L) | 31.49±16.94 | 28.01±12.58 | 0.0024 |
| PLT count (10⁹/L) | 212.17±47.22 | 214.69±46.1 | 0.5152 |
| BMI (kg/m²) | 29.57±5.28 | 30.57±5.74 | 0.0076 |
| HbA1c (%) | 6.17±1.17 | 6.53±1.34 | 0.0004 |
| HDL (mg/dL) | 47.83±2.33 | 44.48±1.82 | <0.0001 |
| TG (mg/dL) | 149.14±15.26 | 171.52±13.13 | 0.0405 |
| Total cholesterol (mg/dL) | 202.39±7.85 | 198.96±6.04 | 0.2649 |
| GLU (mg/dL) | 104.7±3.04 | 105.41±3.03 | 0.3573 |
| WC (cm) | 106.16±6.08 | 106.97±5.34 | 0.0734 |

*Abbreviations: MetALD: metabolic and alcohol related/associated liver disease; MASLD: metabolic dysfunction-associated steatotic liver disease; SD: standard deviation; AST: aspartate transaminase; ALT: alanine aminotransferase; GGT:* *gamma-glutamyl transferase; eGFR: estimated glomerular filtration rate; PLT: platelet; SPB: systolic blood pressure; DBP: diastolic blood pressure; BMI: body mass index; HbA1c:* *hemoglobin A1C; LDL-C: low density lipoprotein cholesterol; HDL-C: high density lipoprotein cholesterol; TG: triglycerides; GLU: fasting glucose; HOMA-IR:* *Homeostatic Model Assessment for Insulin Resistance; WC: waist circumference.*

**Suppl. Table 8.** Comparison of pooled mean values of non-invasive tests (NITs) between non- Asian patients with MetALD, and those with MASLD.

| **NITs (Units)** | **MetALD [pooled mean±SD]** | **MASLD [pooled mean±SD]** | **p-value** |
| --- | --- | --- | --- |
| FIB-4 (–) | 0.96 ± 0.26 | 1.13 ± 0.73 | 0.2286 |
| LSM (kPa) | 8.92 ± 6.79 | 10.01 ± 7.97 | 0.8487 |

*Abbreviations: MetALD: metabolic and alcohol related/associated liver disease; MASLD: metabolic dysfunction-associated steatotic liver disease; FIB-4: fibrosis-4 score; LSM: liver stiffness measurement.*

**Suppl. Table 9.** Comparison of pooled mean levels of clinical and laboratory findings between patients with MetALD, compared to those with MASLD in studies using serum-based non-invasive tests for diagnosis of SLD.

| **Variable (Units)** | **MetALD [pooled mean±SD]** | **MASLD [pooled mean±SD]** | **p value** |
| --- | --- | --- | --- |
| Age (years) | 48.7±5.89 | 51.49±6.48 | <0.0001 |
| AST (IU/L) | 27.17±11.54 | 25.19±6.46 | <0.0001 |
| ALT (IU/L) | 29.26±10.05 | 28.04±8.89 | 0.0033 |
| GGT (IU/L) | 60.33±16.49 | 39.12±7.4 | <0.0001 |
| eGFR (mL/min/1.73 m²) | 89.42±8.63 | 85.88±9.48 | 0.0843 |
| PLT count (10⁹/L) | 255.21±36.49 | 256.82±46.5 | 0.1408 |
| SBP (mmHg) | 129.27±6.43 | 128.15±6.52 | <0.0001 |
| DBP (mmHg) | 80.3±3.76 | 79.68±3.89 | 0.0051 |
| BMI (kg/m²) | 25.82±1.62 | 26.33±1.45 | 0.0093 |
| HbA1c (%) | 5.53±0.24 | 5.62±0.22 | 0.0011 |
| LDL-C (mg/dL) | 109.48±15.33 | 117.27±16.64 | 0.0081 |
| HDL-C (mg/dL) | 51.11±4.62 | 48.11±4.5 | <0.0001 |
| TG (mg/dL) | 188.85±76.92 | 175.12±62.64 | 0.0150 |
| Total cholesterol (mg/dL) | 202.15±12.97 | 204.02±14.37 | 0.4803 |
| GLU (mg/dL) | 99.74±9.47 | 98.63±10.03 | 0.0370 |
| HOMA-IR (-) | 1.72±1.41 | 2.52±2.55 | 0.063 |
| WC (cm) | 87.28±2.34 | 88.19±2.39 | 0.2547 |

*Abbreviations: SLD: steatotic liver disease; MetALD: metabolic and alcohol related/associated liver disease; MASLD: metabolic dysfunction-associated steatotic liver disease; SD: standard deviation, AST: aspartate transaminase; ALT: alanine aminotransferase; GGT:* *gamma-glutamyl transferase; eGFR: estimated glomerular filtration rate; PLT: platelet; SPB: systolic blood pressure; DBP: diastolic blood pressure; BMI: body mass index; HbA1c:* *hemoglobin A1C; LDL-C: low density lipoprotein cholesterol; HDL-C: high density lipoprotein cholesterol; TG: triglycerides; GLU: fasting glucose; HOMA-IR:* *Homeostatic Model Assessment for Insulin Resistance; WC: waist circumference.*

**Supp. Table 10.** Comparison of pooled mean values of non-invasive tests (NITs) between patients with MetALD, and those with MASLD in studies using serum-based non-invasive tests for diagnosis of SLD.

| **NITs (Units)** | **MetALD [pooled mean±SD]** | **MASLD [pooled mean±SD]** | **p-value** |
| --- | --- | --- | --- |
| FIB-4 (–) | 0.9 ± 0.68 | 1.01 ± 0.52 | 0.3126 |

*Abbreviations: SLD: steatotic liver disease; MetALD: metabolic and alcohol related/associated liver disease; MASLD: metabolic dysfunction-associated steatotic liver disease; SD: standard deviation; FLI: fatty liver index; FIB-4: fibrosis-4 score; NFS: non-alcoholic fatty liver disease fibrosis score; LSM: liver stiffness measurement; MRE: magnetic resonance elastography.*

**Suppl. Table 11.** Comparison of pooled mean levels of clinical and laboratory findings between patients with MetALD, compared to those with MASLD in studies using ultrasound for diagnosis of SLD.

| **Variable (Units)** | **MetALD [pooled mean±SD]** | **MASLD [pooled mean±SD]** | **p value** |
| --- | --- | --- | --- |
| Age (years) | 47.14±9.76 | 47.57±10.78 | 0.2301 |
| AST (IU/L) | 29.96±31.17 | 26.61±15.07 | <0.0001 |
| ALT (IU/L) |  |  | 0.0013 |
| GGT (IU/L) | 75.84±80.98 | 42.06±44.58 | <0.0001 |
| eGFR (mL/min/1.73 m²) | 78.16±14.65 | 78.25±14.63 | 0.0038 |
| PLT count (10⁹/L) | 237.56±52.05 | 250.63±56.41 | 0.0003 |
| SBP (mmHg) | 122.24±17.47 | 117.65±15.85 | <0.0001 |
| DBP (mmHg) | 78.76±9.7 | 75.05±9.18 | <0.0001 |
| BMI (kg/m²) | 26.43±3.17 | 26.33±3.27 | 0.6897 |
| HbA1c (%) | 5.84±0.79 | 5.85±0.8 | 0.0022 |
| LDL-C (mg/dL) | 129.35±34.33 | 132.75±34.81 | 0.0638 |
| HDL-C (mg/dL) | 53.65±13.38 | 50.95±11.72 | 0.0038 |
| TG (mg/dL) | 181.45±130.27 | 158.32±98.81 | 0.0666 |
| Total cholesterol (mg/dL) | 206.55±39.36 | 202.49±37.09 | 0.7003 |
| GLU (mg/dL) | 107.3±23.11 | 103±21.06 | 0.0022 |
| HOMA-IR(-) | 1.72±1.41 | 2.69±1.98 | 0.0630 |
| WC (cm) | 91.71±8.18 | 90.16±8.5 | 0.0866 |

*Abbreviations: SLD: steatotic liver disease; MetALD: metabolic and alcohol related/associated liver disease; MASLD: metabolic dysfunction-associated steatotic liver disease; CI: confidence interval, AST: aspartate transaminase; ALT: alanine aminotransferase; GGT:* *gamma-glutamyl transferase; eGFR: estimated glomerular filtration rate; PLT: platelet; SPB: systolic blood pressure; DBP: diastolic blood pressure; BMI: body mass index; HbA1c:* *hemoglobin A1C; LDL-C: low density lipoprotein cholesterol; HDL-C: high density lipoprotein cholesterol; TG: triglycerides; GLU: fasting glucose; HOMA-IR:* *Homeostatic Model Assessment for Insulin Resistance; WC: waist circumference; NA: not available.*

**Supp. Table 12.** Comparison of pooled mean values of non-invasive tests (NITs) between patients with MetALD, compared to those with MASLD in studies using ultrasound for diagnosis of SLD.

| **NITs (Units)** | **MetALD [pooled mean±SD]** | **MASLD [pooled mean±SD]** | **p-value** |
| --- | --- | --- | --- |
| FIB-4 (–) | 1.09 ± 0.64 | 0.97 ± 0.57 | 0.0005 |
| NFS (–) | -2.01±1.19 | -2.27±1.2 | 0.7022 |
| LSM (kPa) | 7.19 ± 4.82 | 9.22 ± 7.21 | 0.9816 |
| MRE (kPa) | 2.66±0.56 | 2.68±0.57 | 0.5393 |

*Abbreviations: SLD: steatotic liver disease; MetALD: metabolic and alcohol related/associated liver disease; MASLD: metabolic dysfunction-associated steatotic liver disease; FIB-4: fibrosis-4 score; NFS: non-alcoholic fatty liver disease fibrosis score; LSM: liver stiffness measurement; MRE: magnetic resonance elastography.*

**Suppl. Table 13.** Comparison of pooled mean levels of clinical and laboratory findings between Asian patients with MetALD, compared to those with ALD.

| **Variable (Units)** | **MetALD [pooled mean±SD]** | **ALD [pooled mean±SD]** | **p value** |
| --- | --- | --- | --- |
| Age (years) | 51.36 ± 6.1 | 50.58 ± 7.06 | 0.9309 |
| AST (IU/L) | 28.69 ± 23.99 | 31.13 ± 23.94 | <0.0001 |
| ALT (IU/L) | 32.33 ± 21.63 | 33.54 ± 24.01 | 0.1039 |
| GGT (IU/L) | 72.38 ± 29.67 | 90.05 ± 42.79 | 0.0301 |
| GFR (mL/min/1.73 m²) | 89.93 ± 9.1 | 109.26 ± 32.93 | 0.0775 |
| PLT count (10⁹/L) | 238.25 ± 52.13 | 242.65 ± 56.39 | 0.9159 |
| SBP (mmHg) | 128.33 ± 14.16 | 128.89 ± 14.35 | <0.0001 |
| DBP (mmHg) | 80.9 ± 9.98 | 81.25 ± 10.09 | <0.0001 |
| BMI (kg/m²) | 25.81 ± 1.89 | 25.53 ± 2.2 | 0.0249 |
| HbA1c (%) | 5.55 ± 0.29 | 5.54 ± 5.45 | 0.0678 |
| LDL-C (mg/dL) | 113.92 ± 40.44 | 111.6 ± 37.38 | 0.0304 |
| HDL-C (mg/dL) | 52.93 ± 14.24 | 52.99 ± 13.56 | 0.0063 |
| TG (mg/dL) | 204.82 ± 143.05 | 215.18 ± 154.8 | 0.3205 |
| Total cholesterol (mg/dL) | 204.06 ± 41.37 | 202.97 ± 16.59 | <0.0001 |
| GLU (mg/dL) | 89.93 ± 9.1 | 109.26 ± 32.93 | 0.1338 |
| WC (cm) | 88.64 ± 7.09 | 88.94 ± 7.37 | <0.0001 |

*Abbreviations: MetALD: metabolic and alcohol related/associated liver disease; ALD:* *alcohol-related liver disease; SD: standard deviation; AST: aspartate transaminase; ALT: alanine aminotransferase; GGT:* *gamma-glutamyl transferase; GFR: glomerular filtration rate; PLT: platelet; SPB: systolic blood pressure; DBP: diastolic blood pressure; BMI: body mass index; HbA1c:* *hemoglobin A1C; LDL-C: low density lipoprotein cholesterol; HDL-C: high density lipoprotein cholesterol; TG: triglycerides; GLU: fasting glucose; HOMA-IR:* *Homeostatic Model Assessment for Insulin Resistance; WC: waist circumference.*

**Suppl. Table 14.** Comparison of pooled mean levels of clinical and laboratory findings between non Asian patients with MetALD, compared to those with ALD.

| **Variable (Units)** | **MetALD [pooled mean±SD]** | **ALD [pooled mean±SD]** | **p value** |
| --- | --- | --- | --- |
| Age (years) | 58.18 ± 12.69 | 55.88 ± 12.41 | 0.3857 |
| AST (IU/L) | 38.18 ± 29.4 | 51.3 ± 41.36 | 0.0030 |
| ALT (IU/L) | 46.7 ± 34.27 | 46.27 ± 33.3 | 0.0087 |
| GGT (IU/L) | 37.25 ± 51.22 | 85.4 ± 53.77 | 0.2832 |
| BMI (kg/m²) | 31.14 ± 5.75 | 29.71 ± 5.87 | 0.0058 |

*Abbreviations: MetALD: metabolic and alcohol related/associated liver disease; ALD:* *alcohol-related liver disease; SD: standard deviation; AST: aspartate transaminase; ALT: alanine aminotransferase; GGT:* *gamma-glutamyl transferase; BMI: body mass index.*

**Suppl. Table 15.** Comparison of pooled mean levels of clinical and laboratory findings between patients with MetALD, compared to those with ALD in studies using serum-based non-invasive tests for diagnosis of SLD.

| **Variable (Units)** | **MetALD [pooled mean±SD]** | **ALD [pooled mean±SD]** | **p value** |
| --- | --- | --- | --- |
| Age (years) | 55.02 ± 9.87 | 54.14 ± 10.35 | 0.4372 |
| AST (IU/L) | 32.38 ± 26.6 | 40.43 ± 32.6 | 0.0117 |
| ALT (IU/L) | 37.56 ± 26.83 | 38.66 ± 27.44 | 0.0287 |
| GGT (IU/L) | 70.36 ± 23.63 | 88.76 ± 37.65 | 0.1102 |
| PLT count (10⁹/L) | 212.5 ± 47.36 | 205.95 ± 52.01 | 0.0238 |
| SBP (mmHg) | 128.88 ± 14.16 | 129.28 ± 14.38 | <0.0001 |
| DBP (mmHg) | 81.13 ± 9.92 | 81.37 ± 10.07 | <0.0001 |
| BMI (kg/m²) | 27.75 ± 3.99 | 27.34 ± 4.55 | 0.0151 |
| HbA1c (%) | 5.83 ± 0.82 | 5.76 ± 3.91 | 0.7272 |
| LDL-C (mg/dL) | 112.67 ± 40.41 | 112.08 ± 35.5 | 0.3374 |
| HDL-C (mg/dL) | 52.27 ± 13.87 | 51.55 ± 12.62 | 0.9208 |
| TG (mg/dL) | 207.74 ± 141.34 | 210.04 ± 146.43 | 0.4565 |
| Total cholesterol (mg/dL) | 203.82 ± 40.08 | 202.92 ± 14.6 | 0.8999 |
| GLU (mg/dL) | 87.92 ± 5.96 | 109.25± 33.27 | 0.2495 |
| WC (cm) | 89.7 ± 6.82 | 91.28 ± 7.03 | 0.6867 |

*Abbreviations: MetALD: metabolic and alcohol related/associated liver disease; ALD:* *alcohol-related liver disease; SD: standard deviation, AST: aspartate transaminase; ALT: alanine aminotransferase; GGT:* *gamma-glutamyl transferase; GFR: glomerular filtration rate; PLT: platelet; SPB: systolic blood pressure; DBP: diastolic blood pressure; BMI: body mass index; HbA1c:* *hemoglobin A1C; LDL-C: low density lipoprotein cholesterol; HDL-C: high density lipoprotein cholesterol; TG: triglycerides; GLU: fasting glucose; HOMA-IR:* *Homeostatic Model Assessment for Insulin Resistance; WC: waist circumference.*

**Suppl. Table 16.** Comparison of pooled mean levels of clinical and laboratory findings between patients with MetALD, compared to those with ALD in studies using ultrasound for diagnosis of SLD.

| **Variable (Units)** | **MetALD [pooled mean±SD]** | **ALD [pooled mean±SD]** | **p value** |
| --- | --- | --- | --- |
| Age (years) | 49.39 ± 10.93 | 47.9 ± 11.39 | 0.6904 |
| AST (IU/L) | 28.24 ± 16.37 | 30.96 ± 28.68 | 0.0003 |
| ALT (IU/L) | 36.93 ± 27.9 | 39.05 ± 32.96 | 0.0935 |
| GGT (IU/L) | 75.72 ± 80.75 | 113.26 ± 136.34 | 0.1490 |
| PLT count (10⁹/L) | 237.19 ± 51.8 | 242.27 ± 56.23 | 0.6229 |
| BMI (kg/m²) | 26.05 ± 3.19 | 25.98 ± 3.31 | 0.1923 |
| HbA1c (%) | 5.88 ± 0.77 | 5.8 ± 0.85 | 0.1030 |
| LDL-C (mg/dL) | 125.5 ± 31.03 | 119.68 ± 32.68 | 0.0016 |
| HDL-C (mg/dL) | 55.05 ± 13.86 | 57.56 ± 15.73 | <0.0001 |
| TG (mg/dL) | 163.9 ± 123.04 | 183.34 ± 142.06 | 0.1951 |
| WC (cm) | 91.01 ± 8.5 | 91.7 ± 8.85 | 0.2750 |

*Abbreviations: MetALD: metabolic and alcohol related/associated liver disease; ALD:* *alcohol-related liver disease; SD: standard deviation, AST: aspartate transaminase; ALT: alanine aminotransferase; GGT:* *gamma-glutamyl transferase; GFR: glomerular filtration rate; PLT: platelet; SPB: systolic blood pressure; DBP: diastolic blood pressure; BMI: body mass index; HbA1c:* *hemoglobin A1C; LDL-C: low density lipoprotein cholesterol; HDL-C: high density lipoprotein cholesterol; TG: triglycerides; WC: waist circumference.*

**Suppl. Table 17.** Sensitivity analysis excluding low-quality studies (NOS ≤5) and conference abstracts comparing pooled mean levels of clinical and laboratory findings between patients with MetALD, compared to those with MASLD.

| **Variable (Units)** | **MetALD [pooled mean±SD]** | **MASLD [pooled mean±SD]** | **p value** |
| --- | --- | --- | --- |
| Age (years) | 49.77±7.17 | 51.73±7.2 | 0.0051 |
| AST (IU/L) | 28.4±14.69 | 25.54±8.24 | <0.0001 |
| ALT (IU/L) | 31.31±15.2 | 28.93±11.9 | <0.0001 |
| GGT (IU/L) | 60.61±20.65 | 39.14±9.66 | <0.0001 |
| eGFR (mL/min/1.73 m²) | 89.23±8.76 | 85.82±9.52 | 0.0590 |
| PLT count (10⁹/L) | 216.59±48.35 | 226.16±50.12 | 0.0082 |
| SBP (mmHg) | 129.23±6.59 | 128.13±6.58 | 0.0010 |
| DBP (mmHg) | 80.29±3.91 | 79.66±3.95 | 0.0002 |
| BMI (kg/m²) | 26.37±2.46 | 26.66±2.08 | 0.0038 |
| HbA1c (%) | 5.83±0.82 | 5.93±0.8 | <0.0001 |
| LDL-C (mg/dL) | 109.75±15.74 | 117.38±16.86 | 0.0027 |
| HDL-C (mg/dL) | 51.15±4.84 | 48.13±4.59 | <0.0001 |
| TG (mg/dL) | 188.03±79.34 | 174.64±63.56 | 0.1180 |
| Total cholesterol (mg/dL) | 202.15±13.03 | 204.02±14.46 | 0.2250 |
| GLU (mg/dL) | 99.85±9.75 | 98.69±10.18 | 0.8776 |
| HOMA-IR (-) | 1.72±1.41 | 2.52±2.55 | 0.0630 |
| WC (cm) | 87.33±2.51 | 88.22±2.5 | 0.2984 |

*Abbreviations: MetALD: metabolic and alcohol related/associated liver disease; MASLD: metabolic dysfunction-associated steatotic liver disease; SD: standard deviation, AST: aspartate transaminase; ALT: alanine aminotransferase; GGT:* *gamma-glutamyl transferase; eGFR: estimated glomerular filtration rate; PLT: platelet; SPB: systolic blood pressure; DBP: diastolic blood pressure; BMI: body mass index; HbA1c:* *hemoglobin A1C; LDL-C: low density lipoprotein cholesterol; HDL-C: high density lipoprotein cholesterol; TG: triglycerides; GLU: fasting glucose; HOMA-IR:* *Homeostatic Model Assessment for Insulin Resistance; WC: waist circumference.*

**Suppl. Table 18.** Sensitivity analysis excluding low-quality studies (NOS ≤5) and conference abstracts comparing the pooled mean values of non-invasive tests (NITs) between patients with MetALD, and those with MASLD.

| **NITs (Units)** | **MetALD [pooled mean±SD]** | **MASLD [pooled mean±SD]** | **p-value** |
| --- | --- | --- | --- |
| FLI (–) | 60.79±18.75 | 52.87±17.37 | <0.0001 |
| FIB-4 (–) | 1.02 ± 0.65 | 0.99 ± 0.56 | 0.3042 |

*Abbreviations: MetALD: metabolic and alcohol related/associated liver disease; MASLD: metabolic dysfunction-associated steatotic liver disease; SD: standard deviation, FLI: fatty liver index; FIB-4: fibrosis-4 score.*

**Suppl. Table 19.** Sensitivity analysis excluding low-quality studies (NOS ≤5) and conference abstracts comparing pooled mean levels of clinical and laboratory findings between patients with MetALD, compared to those with ALD.

| **Variable (Units)** | **MetALD [pooled mean±SD]** | **ALD [pooled mean±SD]** | **p value** |
| --- | --- | --- | --- |
| Age (years) | 54.74±9.93 | 53.59±10.45 | 0.3586 |
| AST (IU/L) | 32.13±26.11 | 39.34±32.21 | 0.0014 |
| ALT (IU/L) | 37.54±26.93 | 38.72±28.19 | 0.0130 |
| GGT (IU/L) | 70.69±31.11 | 89.42±44.29 | 0.0711 |
| eGFR (mL/min/1.73 m²) | 87.96±16.52 | 88.81±15.93 | 0.2075 |
| PLT count (10⁹/L) | 216.03±48.2 | 214.36±53.02 | 0.1658 |
| SBP (mmHg) | 128.54±14.23 | 129.17±14.45 | <0.0001 |
| DBP (mmHg) | 80.96±10 | 81.36±10.12 | <0.0001 |
| BMI (kg/m²) | 27.67±3.96 | 27.22±4.46 | 0.0140 |
| HbA1c (%) | 5.83±0.82 | 5.76±3.89 | 0.2382 |
| LDL-C (mg/dL) | 114.14±39.46 | 112.4±35.39 | 0.4496 |
| HDL-C (mg/dL) | 52.56±13.86 | 51.8±12.77 | 0.2257 |
| TG (mg/dL) | 203.02±140 | 174.64±147.21 | 0.8535 |
| Total cholesterol (mg/dL) | 203.81±40.07 | 209.39±14.78 | 0.1552 |
| GLU (mg/dL) | 89.78±9.15 | 109.33±33 | 0.4258 |
| WC (cm) | 89.86±7.05 | 91.32±7.13 | 0.3586 |

*Abbreviations: MetALD: metabolic and alcohol related/associated liver disease; ALD: alcohol-related liver disease; SD: standard deviation, AST: aspartate transaminase; ALT: alanine aminotransferase; GGT:* *gamma-glutamyl transferase; eGFR: estimated glomerular filtration rate; PLT: platelet; SPB: systolic blood pressure; DBP: diastolic blood pressure; BMI: body mass index; HbA1c:* *hemoglobin A1C; LDL-C: low density lipoprotein cholesterol; HDL-C: high density lipoprotein cholesterol; TG: triglycerides; GLU: fasting glucose; WC: waist circumference.*

**Suppl. Table 20.** Sensitivity analysis excluding low-quality studies (NOS ≤5) and conference abstracts comparing the pooled mean values of non-invasive tests (NITs) between patients with MetALD, and those with ALD.

| **NITs (Units)** | **MetALD [pooled mean±SD]** | **ALD [pooled mean±SD]** | **p-value** |
| --- | --- | --- | --- |
| FIB-4 (–) | 1.01 ± 0.58 | 1.14 ± 0.7 | 0.1838 |

*Abbreviations: MetALD: metabolic and alcohol related/associated liver disease; ALD: metabolic dysfunction-associated steatotic liver disease; SD: standard deviation; FIB-4: fibrosis-4 score.*
