# Supplementary material for: Distinct Laboratory and Clinical Features of Metabolic and Alcohol-Related Liver Disease (MetALD): A Systematic Review and Meta-Analysis
Source: Curr Obes Rep. 2026 Mar 6;15(1):19. doi: 10.1007/s13679-026-00696-6 (PMC12966208; doi:10.1007/s13679-026-00696-6)
Supplement: Supplementary file 2 — Supplementary Material 2 [file 13679_2026_696_MOESM2_ESM.docx]

**APPENDIX**

**Database-specific search**

**Pubmed**

("Metabolic and Alcohol-Related Liver Disease"[Title/Abstract] OR "MetALD"[Title/Abstract] OR ("Steatotic Liver Disease"[ Title/Abstract] AND alcohol*[Title/Abstract] AND metabolic*[Title/Abstract]) OR ("Fatty Liver"[MeSH] AND alcohol*[Title/Abstract] AND metabolic*[Title/Abstract])) AND ("Liver Function Tests"[MeSH] OR "Transaminases"[MeSH] OR "Gamma-Glutamyltransferase"[MeSH] OR "Lipids"[MeSH] OR "Cholesterol"[MeSH] OR "Triglycerides"[MeSH] OR "Glucose"[MeSH] OR "Insulin Resistance"[MeSH] OR "Blood Pressure"[MeSH])

**Embase**

((('metabolic and alcohol-related liver disease':ti,ab,kw OR metald:ti,ab,kw) OR (('steatotic liver disease'/exp OR 'fatty liver'/exp) AND alcohol*:ti,ab,kw AND metabolic*:ti,ab,kw))) AND ('liver function test'/exp OR 'transaminase'/exp OR 'gamma glutamyltransferase'/exp OR 'lipid'/exp OR 'cholesterol'/exp OR 'triglyceride'/exp OR 'glucose'/exp OR 'insulin resistance'/exp OR 'blood pressure'/exp)

**Cochrane:**

((("Metabolic and Alcohol-Related Liver Disease"):ti,ab,kw OR (MetALD):ti,ab,kw) OR ((MeSH descriptor: [Steatotic Liver Disease] explode all trees OR MeSH descriptor: [Fatty Liver] explode all trees) AND (alcohol*):ti,ab,kw AND (metabolic*):ti,ab,kw)) AND (MeSH descriptor: [Liver Function Tests] explode all trees OR MeSH descriptor: [Transaminases] explode all trees OR MeSH descriptor: [Gamma-Glutamyltransferase] explode all trees OR MeSH descriptor: [Lipids] explode all trees OR MeSH descriptor: [Cholesterol] explode all trees OR MeSH descriptor: [Triglycerides] explode all trees OR MeSH descriptor: [Glucose] explode all trees OR MeSH descriptor: [Insulin Resistance] explode all trees OR MeSH descriptor: [Blood Pressure] explode all trees)

**Scopus**

(TITLE-ABS-KEY("metabolic and alcohol-related liver disease" OR metald) OR (TITLE-ABS-KEY("steatotic liver disease" OR "fatty liver") AND TITLE-ABS-KEY(alcohol*) AND TITLE-ABS-KEY(metabolic*))) AND TITLE-ABS-KEY( "liver function test*" OR transaminase* OR "gamma-glutamyltransferase" OR ggt OR lipid* OR cholesterol OR triglyceride* OR glucose OR insulin OR "insulin resistance" OR "blood pressure" OR hypertension)
